# Genome-wide identification of the NLR gene family in *Haynaldia villosa* by SMRT-RenSeq

**DOI:** 10.1186/s12864-022-08334-w

**Published:** 2022-02-10

**Authors:** Zhenpu Huang, Fangyuan Qiao, Boming Yang, Jiaqian Liu, Yangqi Liu, Brande B. H. Wulff, Ping Hu, Zengshuai Lv, Ruiqi Zhang, Peidu Chen, Liping Xing, Aizhong Cao

**Affiliations:** 1 National Key Laboratory of Crop Genetics and Germplasm Enhancement, Cytogenetics Institute, Nanjing Agricultural University/CIC-MCP, Nanjing, 210095 China; 2 John Innes Centre, Norwich Research Park, Norwich, NR4 7UH UK; 3 Center for Desert Agriculture, Biological and Environmental Science and Engineering Division (BESE), King Abdullah University of Science and Technology (KAUST), Thuwal, 23955-6900 Saudi Arabia

**Keywords:** NLR, *Haynaldia villosa*, SMRT-RenSeq, Disease resistance, Genomics

## Abstract

**Background:**

Nucleotide-binding and leucine-rich repeat (NLR) genes have attracted wide attention due to their crucial role in protecting plants from pathogens. SMRT-RenSeq, combining PacBio sequencing after resistance gene enrichment sequencing (RenSeq), is a powerful method for selectively capturing and sequencing full-length NLRs. *Haynaldia villosa*, a wild grass species with a proven potential for wheat improvement, confers resistance to multiple diseases. So, genome-wide identification of the NLR gene family in *Haynaldia villosa* by SMRT-RenSeq can facilitate disease resistance genes exploration.

**Results:**

In this study, SMRT-RenSeq was performed to identify the genome-wide *NLR* complement of *H. villosa*. In total, 1320 NLRs were annotated in 1169 contigs, including 772 complete *NLRs*. All the complete NLRs were phylogenetically analyzed and 11 main clades with special characteristics were derived. *NLRs* could be captured with high efficiency when aligned with cloned R genes, and cluster expansion in some specific gene loci was observed. The physical location of *NLRs* to individual chromosomes in *H. villosa* showed a perfect homoeologous relationship with group 1, 2, 3, 5 and 6 of other *Triticeae* species, however, *NLRs* physically located on 4VL were largely in silico predicted to be located on the homoeologous group 7. Fifteen types of integrated domains (IDs) were integrated in 52 NLRs, and Kelch and B3 NLR-IDs were found to have expanded in *H. villosa*, while DUF948, NAM-associated and PRT_C were detected as unique integrated domains implying the new emergence of NLR-IDs after *H. villosa* diverged from other species.

**Conclusion:**

SMRT-RenSeq is a powerful tool to identify *NLR* genes from wild species using the baits of the evolutionary related species with reference sequences. The availability of the *NLRs* from *H. villosa* provide a valuable library for R gene mining and transfer of disease resistance into wheat.

**Supplementary Information:**

The online version contains supplementary material available at 10.1186/s12864-022-08334-w.

## Background

Plants have evolved comprehensive mechanisms to protect themselves from attack by pests and pathogens [18]. The first level of protection is provided by the physical barriers imposed by the plant surface, a type of resistance often termed passive defense [70]. The second level of protection is induced by recognition of pathogen associated molecular patterns (PAMPs) by pattern-recognition receptors (PRRs), which are usually extracellular plasma membrane anchored; this active defense is often called PAMP-triggered immunity (PTI) [7]. The last level of protection is induced by recognition of pathogen effectors by the products of plant resistance genes usually located in the cytoplasm; this active defense is often called effector-triggered immunity (ETI) [28].

To date, more than 300 resistance (*R* genes) defined by genetics have been cloned from a wide range of plant species [36]. The majority of these (> 80%) encode intracellular immune receptors of the nucleotide-binding and leucine-rich repeat (NLR) class of genes. NLRs have also been found to induce defense responses in animals [29, 48]. Genomic reference quality assemblies now make it possible to characterize complete *NLR* repertoires in plants. Typically, several hundred *NLRs* are found in a plant genome. For example, 149 *NLRs* were identified in *Arabidopsis* [50], 459 in *Vitis vinifera*, 330 in *Populus trichocarpa* [81], 319 in *Glycine max* [35] and 327 in *Manihot esculenta* [46]. Genome-wide *NLR* complements have also been studied in five species of *Brassicaceae* [88], four species of *Gossypium* [78], seven species of *Leguminosae* [89], three species of *Solanaceae* [60], and in several species of grasses including *Sorghum bicolor*, *Zea mays*, *Brachypodium distachion*, cultivated and wild *Oryza* species, and several *Triticeae* species [4, 15, 41, 61, 69, 80]. In hexaploid bread wheat, 3400 *NLRs* were identified, the largest number reported thus far in a plant species [69]. The number of NLRs appears to correlate positively with the total number of genes in the genome [3], but less so with genome size. For example, more than 1000 NLRs were identified in apple (740 Mb genome [27];), while only 151 were detected in maize (2.1 Gb genome [66];) and 54 in *Carica papaya* (370 Mb genome [58];). However, higher ploidy levels does tend to correlate with a larger number of *NLRs*, such as observed in wheat and apple [63, 73].

With the recent rapid advances in bioinformatics and genomics, huge progress has been made in understanding *Triticeae* genomes, including more efficient and complete characterization of their *NLR* complements. The *NLR* contents have been identified in *T. urartu*, *Ae. tauschii* and *T. aestivum* by different researchers [22, 69]. However, due to different draft genome versions, distinct annotation pipelines with various parameters and the high sequence similarity among NLRs, the number of identified NLRs can vary widely between different studies. In those species which lack a reference genome, it is even more challenging to perform a genome-wide NLR survey. Exon capture enrichment allows the selected sequencing of an exome [54], or a specific gene family [32]. The highly conserved domains shared by different NLRs provides perfect targets for enrichment. The *R* gene enrichment and sequencing (RenSeq) method provides a powerful and attractive tool for the identification of NLRs from plants without finished reference genome assemblies, in particular those plants with large genomes and higher ploidy levels, for example hexaploid wheat and octaploid strawberry [6]. RenSeq was first applied to identify NLRs from *Solanum tuberosum*, and it indicated that ~ 80% sequence identity between NLR genes and the corresponding oligonucleotide baits was sufficient for enrichment. This pioneering use of RenSeq increased the number of annotated NLRs in potato from 438 to 772, and facilitated the genetic mapping of NLRs associated with disease resistance to poorly or previously unannotated regions of the genome [34]. MutRenSeq, combining RenSeq with mutant development, was used to clone the stem rust resistance genes *Sr22* and *Sr45* from hexaploid wheat [68]. More recently, association genetics combined with RenSeq (AgRenSeq) on a wild population of diploid wheat (*Aegilops tauschii*), AgRenSeq, facilitated the rapid identification four stem rust resistance genes [2]. The assemblies generated by RenSeq with short-read Illumina sequencing technology are, however, typically fragmented and incomplete. For example, RenSeq targeting *Sr22* and *Sr33* resulted in two and three contigs, respectively, with missing gaps corresponding largely to the introns. RenSeq combined with long read sequencing, such as PacBio single-molecule real-time (SMRT) or Oxford Nanopore Technology, mitigates these limitations by generating more complete assemblies including NLRs with novel integrated domains [19, 76]. SMRT-RenSeq facilitated the cloning of the *Phytophothora infestans* resistance gene *Rpi-amr3i* from the wild potato relative *Solanum americanum* [76], the *Potato Virus Y* resistance genes *RySto* from the wild potato relative *Solanum stoloniferum* [21], the powdery mildew resistance gene *Pm21* from the wild wheat relative *Haynaldia villosa* [79], and a species-wide inventory of NLR genes and alleles in *Arabidopsis thaliana* [71].

Two sequential polyploidization events followed by domestication and intensive breeding have narrowed the genetic diversity in cultivated wheat [57, 90]. This dearth of diversity can be offset by introducing natural variation from wild species through wide crosses. In disease resistance breeding, resistance has been introgressed into wheat from at least 52 species from 13 genera due to the remarkable plasticity of the wheat genome [77]. *Haynaldia villosa* (genome constitution VV, 2n = 14) is a wild diploid species of wheat, which has been introduced into hexaploid bread wheat by the development of individual chromosome arm translocation lines between *H. villosa* and wheat. We previously reported the introgression from *H. villosa* into wheat of the powdery mildew resistance genes *Pm21*, *Pm55* and *Pm62*, the yellow virus resistance gene *Wss1* and the cereal cyst nematode resistance gene *CreV* [14, 16, 83–85]. Moreover, resistance to leaf rust, stripe rust, take-all, and sharp eye-spot resistances conferred by *H. villosa* chromatin in wheat have also been reported [26, 43, 52, 59]. The broad-spectrum resistance gene *Pm21* has been widely used in wheat breeding and more than 40 new varieties have been released and cultivated in regions of China where powdery mildew is prevalent. *Pm55* and *Wss1* have also been used in breeding programs and new wheat lines are currently under evaluation. Cloning of these resistance genes will accelerate their use by genetic engineering to reduce linkage drag. However, the lack of a reference genome and a high level of outcrossing of *H. villosa* makes resistance gene cloning in this species challenging.

Previously, we used SMRT-RenSeq to de novo assemble the NLRs of the inbred *H. villosa* accession 91C43. Then, NLR-Parser [67] was used to identify NLRs from the 1509 contigs, and 485 full-length NLRs were annotated [79]. In 2018, NLR-Annotator, the improved version for NLR prediction, was released (https://github.com/steuernb/NLR-Annotator). The improved software differs from NLR-Parser in that it can distinguish the border between different NLRs located in long contigs [69]. Since many NLRs are tightly clustered in plant genomes [46], we hypothesized that NLR-Annotator would identify a larger number of NLRs than previously predicted by NLR-Parser. Here NLR-Annotator was used to identify NLRs from *H. villosa*, followed by determination of the NLR classes, assignment of NLRs to chromosomes, exploration of NLRs orthologous to cloned R genes, identification of NLR-IDs and comparison of NLRs among different *Triticeae* species. Our study provides a valuable resource to support *R* gene cloning in *H. villosa* and in *H. villosa*-wheat introgression lines.

## Results

### NLR annotation in the SMRT-RenSeq assembly

Previously, 406 Mb of SMRT-RenSeq data consisting of 107,153 reads with an average length of 4.5 kb were generated and de novo assembled with Geneious v9.1.4 (Fig. S1). This assembly generated 1509 contigs in which 80% of the contigs ranged from 5 kb to 11 kb, with the largest one spanning 24.6 kb (Fig. S2). The data was analyzed following the pipeline described in Fig. S3. In the present study, the 1509 contigs were re-analysed. Firstly, contigs sharing more than 95% identity were removed leaving 1456 non-redundant contigs. Secondly, sequences with low-complexity and with interspersed repeats were masked. Thirdly, and importantly, the contigs were annotated by NLR-Annotator, which, unlike our previous analysis, allows detection of multiple NLRs on the same contig. In total, 1320 NLRs were annotated in 1169 contigs, including 776 complete NLRs, 289 complete (pseudogene) NLRs, 188 partial NLRs and 67 partial (pseudogene) NLRs.

### Prediction and analysis of NLR domain composition

**Fig. 1:**
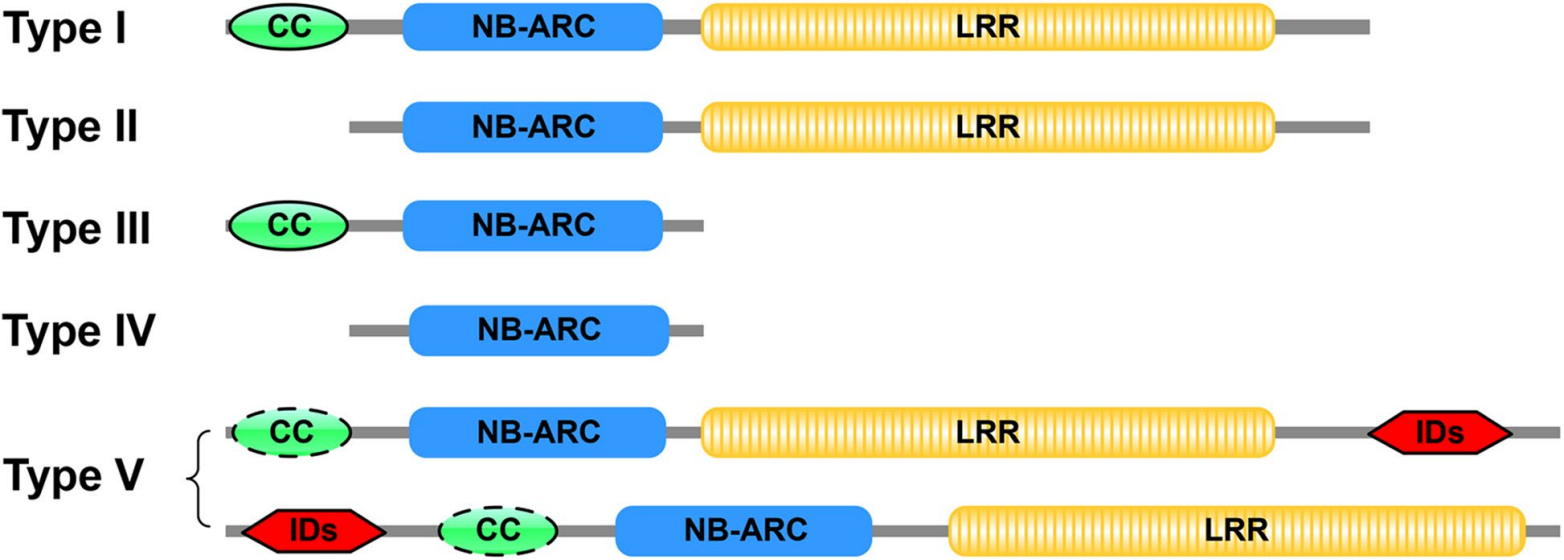
Classification of NLRs detected in *H. villosa*. Based on sub-domain analysis, the 772 annotated NLRs were divided into five subclasses as Type I to Type V, among which 618 NLRs belong to Type I (CC-NB-LRR), 98 belong to Type II (NB-LRR), three belong to Type III (CC-NB), one belongs to Type IV (NB-ARC) and 52 belong to Type V (NLR-ID). Type V contains two subtypes with integrated domains located in N-terminal or C-terminal respectively, and the CC domain with the dotted line shows that it may not be present

To obtain the corresponding protein sequences of the annotated full-length NLRs, the genomic sequences of the identified NLRs were used to search the protein database of barley, wheat and *Ae. tauschii* using BLASTx. Then, the predicted *H. villosa* NLR protein sequences were obtained using FGENESH+ based on the homologous proteins. The 776 complete NLR protein candidates were analyzed by Plant_rgene to search for coiled-coil (CC), NB-ARC and integrated domains, then re-analyzed by InterProScan to search for the conserved LRR domains deposited in the SUPERFAMILY database. The results indicated that 772 members carried an NB-ARC, so these 772 candidates were considered to be bona fide NLRs. Then the 772 NLR proteins were further divided into five subclasses based on sub-domain analysis; 618 NLRs were typed as CC-NB-LRR (CNL), 98 as NB-LRR (NL), three as CC-NB (CN), one as NB-ARC (N) and 52 as NLRs with integrated domains (NLR-ID) (Fig. 1).

The proteins were also downloaded from the newly released genomic database of several grass species, including *T. aestivum*, *T. urartu*, *Ae. tauschii*, *H. vulgare*, *O. sativa* and *B. distachyon*. Then, the NLRs in these species were identified using the same procedure as outlined above. The *Triticeae* B genome has the largest number of NLRs (834), followed by the V genome with 772 NLRs (Table 1). The *Triticeae* A, B, D, H and V genomes with an average of 632 ± 148 NLRs harbor more NLRs than *O. sativa* (347 NLRs) and *B. distachyon* (350 NLRs), suggesting that *Triticeae* species have experienced NLR expansion more rapidly after diverging from *O. sativa* and *B. distachyon*. An NLR expansion in the A and D genome was also observed after polyploidization of wheat (Table 1).

**Table 1:** NLRs identified in *H. villosa* and evolutionary related species

Species	Total NLR genes	CNL		NL		CN		N		NLR-ID	
		Num.	Pct.	Num.	Pct.	Num.	Pct.	Num.	Pct.	Num.	Pct.
*B. distachyon*	350	201	57.4%	60	17.1%	53	15.1%	18	5.1%	16	4.6%
*O. sativa*	347	156	45.0%	70	20.2%	77	22.2%	35	10.1%	7	2.0%
*H. vulgare*	397	198	50.0%	84	21.2%	60	15.1%	31	7.8%	24	6.1%
*T. urartu*	530	275	52.0%	107	20.2%	78	14.7%	24	4.5%	45	8.5%
*A. tauschii*	572	298	52.1%	113	19.8%	70	12.2%	50	8.7%	41	7.2%
*T. aestivum*	2273	1181	52.0%	367	16.2%	493	21.7%	98	4.3%	130	5.7%
(A genome)	(672)	346	51.5%	102	15.2%	153	22.8%	32	4.8%	38	5.7%
(B genome)	(834)	443	53.1%	146	17.5%	169	20.3%	27	3.2%	47	5.7%
(D genome)	(647)	339	52.4%	100	15.5%	135	20.9%	34	5.7%	38	5.9%
*H. villosa*	772	618	80.1%	98	12.7%	3	0.39%	1	0.13%	52	6.8%

Note: *Num.* number, *Pct.* percentage (%)

The domain compositions of all the NLRs were analyzed by InterProScan and Plant_rgene. Unusually for *H. villosa*, 80% of the NLRs were of the CNL type compared to ~ 50% in the other species. At first glance, this may suggest that different types of NLRs have traversed different evolutionary paths within the *Triticeae*. Alternatively, since the ‘complete NLR’ annotated by NLR-Annotator is defined as a sequence containing the P-loop, the start of the NB-ARC domain, as well as at least one LRR motif, the CN and N types NLRs were likely filtered out by our analysis.

### Phylogenetic analysis of NLRs in *H. villosa*

**Fig. 2:**
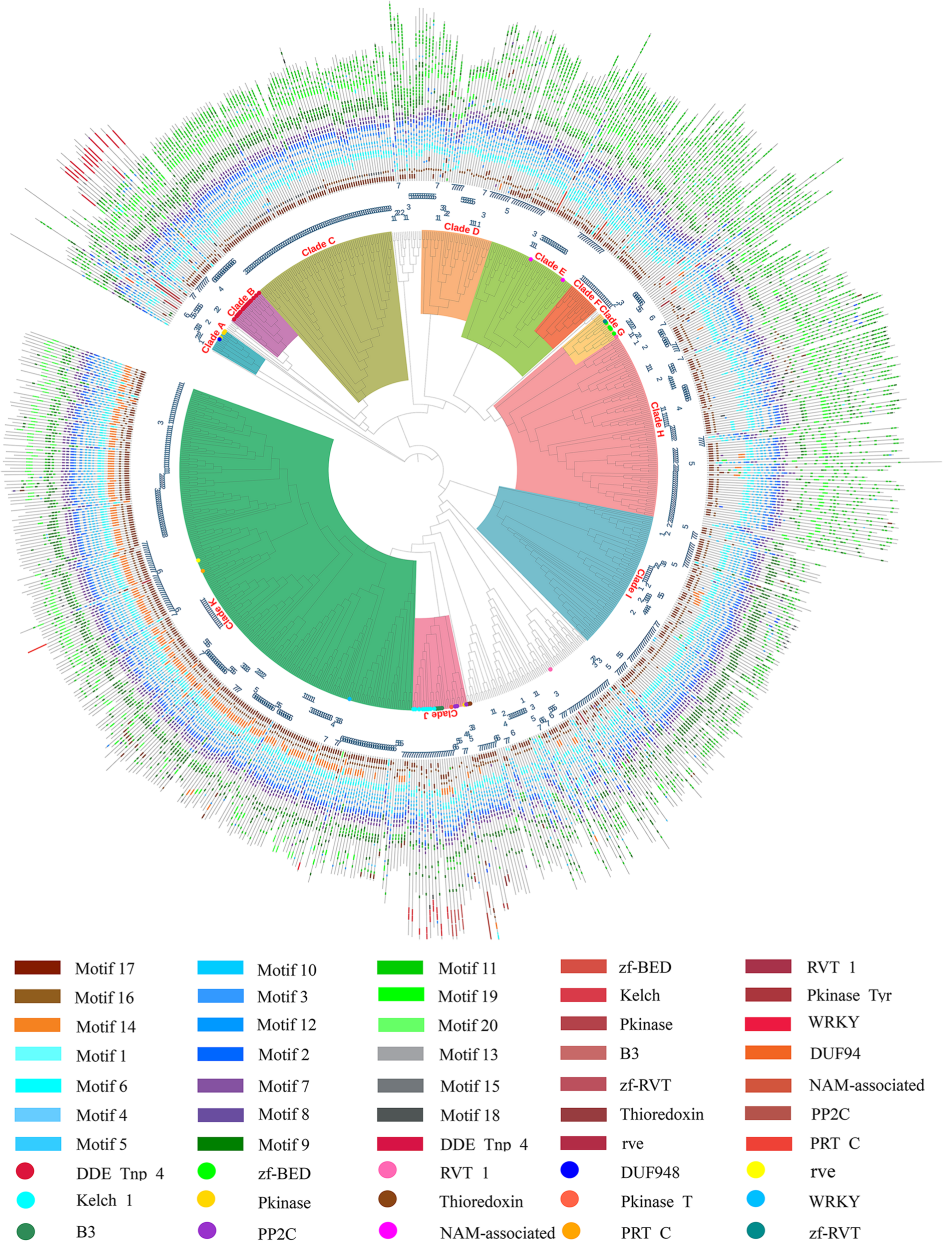
Phylogenetic analysis of 772 NLRs based on the NB-ARC domain. The phylogenetic tree of the 772 NLR of *H. villosa* was constructed based on the sequences of the NB-ARC domain using MEGA7 by Neighbor-Joining method with 1000 bootstrap replicates, and the tree was visualized using iTOL (https://itol.embl.de/shared/2018201031). Eleven main clades were displayed using different colors in the tree, and the conserved motifs from each complete NLR were displayed using different colors in the domain compositions. Number 1 to 7 present in silico localization of NLR genes on chromosome 1V to 7V

All 772 NLRs of *H. villosa* were used to construct a phylogenetic tree, based on the sequences of the NB-ARC domain, to reveal the potential evolutionary relationships (Fig. 2, https://itol.embl.de/shared/2018201031). The conserved motifs from each complete NLR were displayed in the phylogenetic tree to track the evolutionary characteristics of each clade; 11 main clades were thus derived and displayed in different colors. The structure and characteristics of each clade was analyzed according to the motif classification by Jupe [33]. Clade A is composed of NLs lacking CC domains. Clade B contains NLR-IDs carrying exclusively the DDE_Tnp_4 domain. Clade C members lack the NB-ARC ‘motif 10’, and the CC ‘motif 17’ is followed by ‘motif 15’ but not by ‘motif 16’ as is usually the case. ‘Motif 15’, also referred to as ‘TIR-2’, is found in both monocots and dicots [33]. In this study the annotated NLRs containing this motif were still classified as being CNL but not TNL due to the CC domain being detected. All the NLRs in clade C are located on chromosome 3 V, indicating that this type of NLR has experienced active expansion but not migration. Clade D members contain only ‘motif 16’ in the CC domain but lack ‘motif 17’. Clade E NB-ARC domains contain an additional ‘motif 6’ followed by ‘motif 1’. Clade H members contain a longer linker between the CC domain and the NB-ARC domain, and the LRR domain is more irregular. Clade I contains three or four tandem repeats of ‘motif 9’ following the linker. Clade F lacks ‘motif 10’ in the NB-ARC domain. Clade G and J members tend to contain one of either three or five, respectively, integrated domains. Clade K is mainly composed of CNLs and a few NLs. In this clade, most members contain ‘motif 14’ between the CC and NB-ARC domains, a signature reported to be unique in monocots [33]. Another characteristic of clade K is that eight motifs associated with the NB-ARC domain are arranged in a uniform order. Compared to the CC and NB-ARC domains, a larger diversity was found in the LRR domain, such as motif numbers and arrangements, possibly reflecting the role of the LRRs in recognizing pathogen effectors.

### NLR expression analysis

We sequenced a full-length transcriptome of *H. villosa* with PacBio SMRT chemistry. To detect expressed NLRs and to verify the predicted exon/intron boundaries, the 772 NLRs were searched against NLRs annotated in the transcriptome database. This revealed 91 NLRs with a transcript with more than > 95% identity and a query coverage > 90%. Sequence alignment between the transcript and the genomic DNA of these 91 NLRs indicated that the splicing pattern of 85 of the NLRs perfectly matched our predictions. The remaining 6 expressed *NLRs*, whose splicing patterns didn’t match with the predictions, were used to test whether specific splicing patterns happened in *H. villosa* by comparing their splicing patterns with those of the corresponding orthologous *NLRs*. The comparisons indicated that there were no specific splicing patterns happened in 3 *NLRs*, including Hv_Contig_1470_nlr_1, Hv_Contig_696_nlr_1 and Hv_Contig_1466_nlr_1, while the coding lengths were different between the predicted sequences and the expressed sequences. For Hv_Contig_249_nlr_2, its orthologous NLRs in wheat barley and *Ae. tauschii* contained A and B types of splicing patterns, and the previously predicted CDS was corresponding to the A type, while the expressed CDS was corresponding to the B type. So, there was no specific splicing pattern in Hv_Contig_249_nlr_2. However, the specific splicing patterns happened in Hv_Contig_670_nlr_1 and Hv_Contig_61_nlr_1. Knowledge of NLR expression can provide additional powerful support for *R* gene cloning.

### Assignment of NLRs to chromosomes

*H. villosa* displays good chromosome collinearity with barley, *Ae. tauchii*, *Triticum urartu* and wheat. Therefore the nucleotide sequences of the 772 complete NLRs were used as a query for BLASTn analysis against the genome databases of these species for in silico prediction of chromosomal location. This procedure assigned all 772 complete NLRs to homoelogous chromosomes; 139 NLRs to group 1, 85 to group 2, 164 to group 3, 15 to group 4, 60 to group 5, 101 to group 6, and 208 to group 7 (Table S3). However, due to the differentiation between the *H. villosa* genome and those of the species used in the analysis, we decided to independently confirm the assignment for a subset of NLRs. To this end, we took advantage of a previously developed full set of wheat-*H. villosa* translocation lines, each involving one of the 14 chromosome arms of *H. villosa* [86], to physically locate NLRs using PCR molecular markers. A subset of the in silico mapped NLRs were selected evenly from the seven chromosomes. The sequence of each selected *NLR* was aligned with the predicted wheat orthologues on the A, B, and D genome, then primers were designed based on insertions or deletions private to *H. villosa*. A total of 757 primer pairs were designed corresponding to 565 contigs, of which 105 primer pairs gave rise to polymorphisms between *H. villosa* and wheat. Thus, the polymorphism rate produced using InDel-markers based on NLRs was 14%, which is significantly lower than the 52% produced using IT (Intron-Target)-markers based on single copy genes [87]. The high sequence similarity between NLRs likely complicates the design of polymorphic markers.

**Fig. 3:**
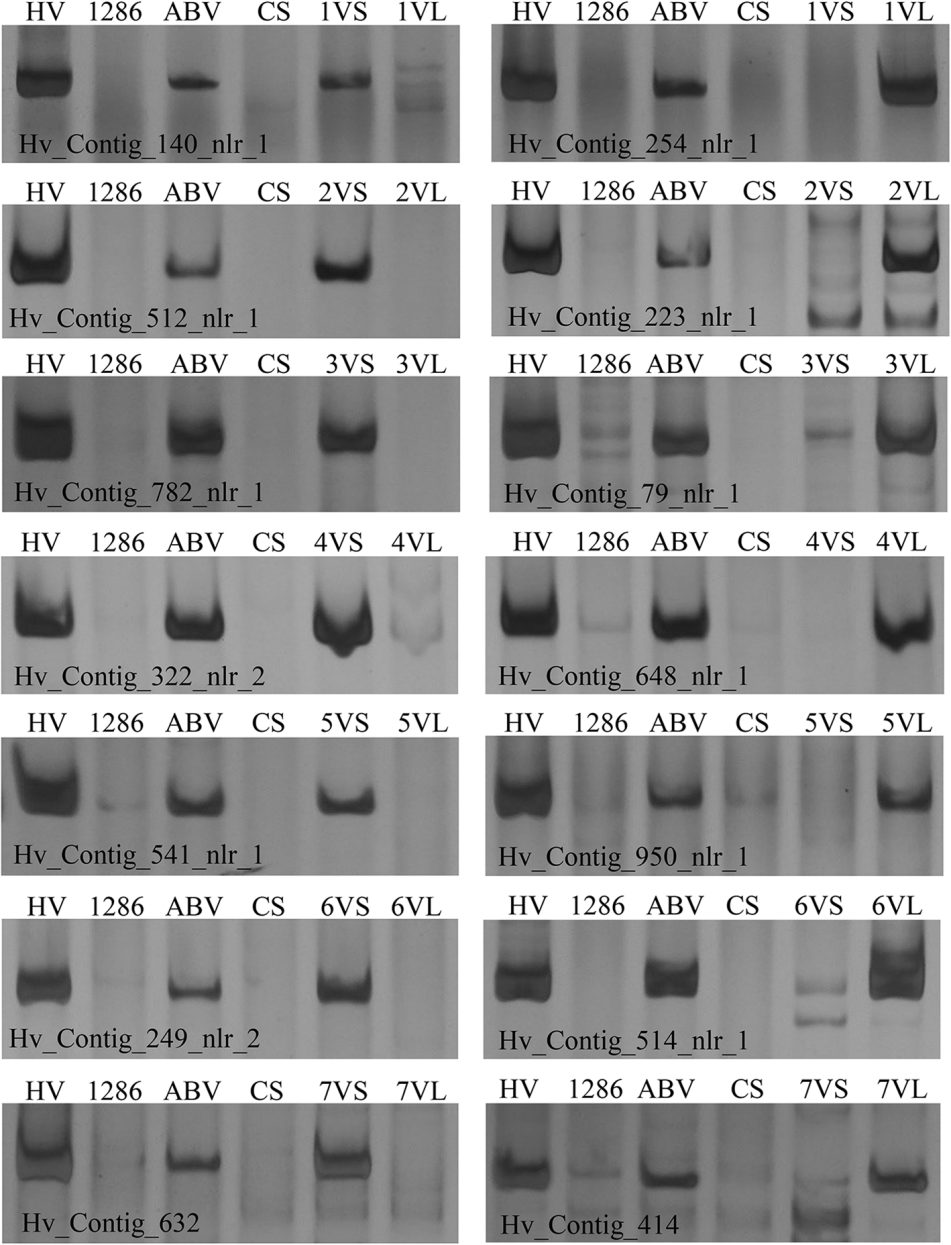
Chromosomal location of the annotated NLRs using a full set of 14 whole arm translocation lines. HV: *H. villosa* (2n = 14, VV); ZY1286: *T. turgidum* tetraploid wheat (2n = 28, AABB); ABV: *T. turgidum*-*H. villosa* hexaploid amphiploid wheat (2n = 42, AABBVV); CS: hexaploid wheat Chinese Spring (2n = 42, AABBDD), 1 VS, 2 VS, 3 VS, 4VS, 5 VS, 6VS, 7 VS: wheat-*H. villosa* translocation lines involving the short arm of 1 V, 2 V, 3 V, 4 V, 5 V, 6 V, 7 V; 1 VL, 2 VL, 3 VL, 4VL, 5 VL, 6VL, 7 VL: wheat-*H. villosa* translocation lines involved the long arm of 1 V, 2 V, 3 V, 4 V, 5 V, 6 V, 7 V

From the 105 polymorphic primer pairs, 61 complete and 26 partial (pseudogene) NLRs could be located to specific chromosome arms (Fig. 3; Table 2). All the NLRs located in silico on homoeologous groups 1, 2, 3, 5 and 6 were physically located on chromosome 1 V, 2 V, 3 V, 5 V and 6 V, respectively, thus showing a perfect homoeologous relationship between groups 1, 2, 3, 5 and 6, respectively, of *H. villosa* and other *Triticum* species (Table 2). As to the distribution of NLRs on 4 V, the NLRs physically located on 4VS were located in silico on homoeologous groups 4, however, NLRs physically located on 4VL were largely in silico predicted to be located on the homoeologous group 7 (Table 2). It was also previously reported that four 4VL-specific markers of *H. villosa* detected homoeoloci on the group 7 chromosomes of wheat [86]. Therefore, our data, and those of Zhang et al. [86], suggest that 4VL did not translocate with 7 VS, unlike the reciprocal 4 L/7S translocation which happened in wheat and other *Triticeae* species.

**Table 2:** Chromosomal location of the identified *NLRs* in *H. villosa*

NLR genes	NLR status	Chromosomal Location			
		***Triticum aestivum***	***Hordeum vulgare***	***Aegilops tauschii***	***Haynaldia villosa***
Hv_Contig_60_nlr_3	Complete NLR	1D	1H	1D	1 VS
Hv_Contig_138_nlr_2	Complete NLR	1A	1H	1D	1 VS
Hv_Contig_140_nlr_1	Complete NLR	1D	1H	1D	1 VS
Hv_Contig_232_nlr_1	Complete NLR	1B	1H	1D	1 VS
Hv_Contig_443_nlr_1	Complete NLR	1B	1H	1D	1 VS
Hv_Contig_992_nlr_1	Complete NLR	1A	1H	1D	1 VS
Hv_Contig_1391_nlr_1	Complete NLR	1A	1H	1D	1 VS
Hv_Contig_254_nlr_1	Complete NLR	1A	1H	1D	1 VL
Hv_Contig_452_nlr_1	Complete NLR	1D	1H	1D	1 VL
Hv_Contig_1453_nlr_1	Complete NLR	1D	1H	1D	1 VL
Hv_Contig_951	Partial or pseudogene	1A	3H	1D	1 VS
Hv_Contig_1193	Partial or pseudogene	1D	1H	1D	1 VS
Hv_Contig_141	Partial or pseudogene	1A	1H	1D	1 VL
Hv_Contig_219	Partial or pseudogene	1D	1H	1D	1 VL
Hv_Contig_512_nlr_1	Complete NLR	2A	2H	2D	2 VS
Hv_Contig_544_nlr_2	Complete NLR	2D	2H	2D	2 VS
Hv_Contig_223_nlr_1	Complete NLR	2B	2H	2D	2 VL
Hv_Contig_1028_nlr_1	Complete NLR	2D	2H	2D	2 VL
Hv_Contig_461_nlr_1	Complete NLR	2D	2H	2D	2 VL
Hv_Contig_1254	Partial or pseudogene	Un	2H	2D	2 VS
Hv_Contig_35	Partial or pseudogene	2D	2H	2D	2 VL
Hv_Contig_139	Partial or pseudogene	2D	2H	2D	2 VL
Hv_Contig_667	Partial or pseudogene	2B	2H	2D	2 VL
Hv_Contig_716_nlr_1	Complete NLR	3A	3H	3D	3 VS
Hv_Contig_782_nlr_1	Complete NLR	3B	3H	3D	3 VS
Hv_Contig_11_nlr_1	Complete NLR	3D	3H	3D	3 VL
Hv_Contig_79_nlr_1	Complete NLR	3B	3H	3D	3 VL
Hv_Contig_326_nlr_1	Complete NLR	3D	3H	3D	3 VL
Hv_Contig_657_nlr_2	Complete NLR	3A	3H	3D	3 VL
Hv_Contig_866_nlr_1	Complete NLR	3B	3H	3D	3 VL
Hv_Contig_77	Partial or pseudogene	3A	3H	3D	3 VL
Hv_Contig_686	Partial or pseudogene	3B	3H	3D	3 VL
Hv_Contig_90_nlr_1	Complete NLR	4B	Un	4D	4VS
Hv_Contig_116_nlr_1	Complete NLR	4D	4H	4D	4VS
Hv_Contig_322_nlr_2	Complete NLR	4B	4H	4D	4VS
Hv_Contig_670_nlr_1	Complete NLR	4B	4H	4D	4VS
Hv_Contig_28_nlr_1	Complete NLR	7B	7H	7D	4VL
Hv_Contig_55_nlr_1	Complete NLR	7D	7H	7D	4VL
Hv_Contig_82_nlr_2	Complete NLR	7D	7H	7D	4VL
Hv_Contig_172_nlr_1	Complete NLR	7B	7H	7D	4VL
Hv_Contig_299_nlr_1	Complete NLR	7D	7H	7D	4VL
Hv_Contig_393_nlr_1	Complete NLR	7A	7H	7D	4VL
Hv_Contig_648_nlr_1	Complete NLR	7D	7H	7D	4VL
Hv_Contig_798_nlr_1	Complete NLR	7D	7H	7D	4VL
Hv_Contig_913_nlr_2	Complete NLR	7D	7H	7D	4VL
Hv_Contig_958_nlr_1	Complete NLR	7B	7H	7D	4VL
Hv_Contig_1239_nlr_1	Complete NLR	7A	7H	7D	4VL
Hv_Contig_1300_nlr_1	Complete NLR	7D	7H	7D	4VL
Hv_Contig_1318_nlr_1	Complete NLR	7D	7H	7D	4VL
Hv_Contig_1410_nlr_1	Complete NLR	7D	7H	7D	4VL
Hv_Contig_120	Partial or pseudogene	4B	4H	4D	4VS
Hv_Contig_362	Partial or pseudogene	4A	7H	7D	4VL
Hv_Contig_1235	Partial or pseudogene	7A	2H	7D	4VL
Hv_Contig_1339	Partial or pseudogene	4A	7H	7D	4VL
Hv_Contig_541_nlr_1	Complete NLR	5D	5H	5D	5 VS
Hv_Contig_1146_nlr_1	Complete NLR	7D	5H	7D	5 VS
Hv_Contig_105_nlr_1	Complete NLR	5D	5H	5D	5 VL
Hv_Contig_253_nlr_1	Complete NLR	5A	5H	5D	5 VL
Hv_Contig_308_nlr_1	Complete NLR	5B	5H	7D	5 VL
Hv_Contig_705_nlr_1	Complete NLR	5D	5H	5D	5 VL
Hv_Contig_757_nlr_1	Complete NLR	5D	5H	5D	5 VL
Hv_Contig_937_nlr_1	Complete NLR	5B	Un	5D	5 VL
Hv_Contig_950_nlr_1	Complete NLR	5B	5H	5D	5 VL
Hv_Contig_85	Partial or pseudogene	5B	5H	2D	5 VS
Hv_Contig_270	Partial or pseudogene	5B	5H	5D	5 VL
Hv_Contig_1353	Partial or pseudogene	5A	5H	6D	5 VL
Hv_Contig_665_nlr_1	Complete NLR	6B	6H	6D	6VS
Hv_Contig_39_nlr_2	Complete NLR	6B	6H	6D	6VS
Hv_Contig_249_nlr_2	Complete NLR	6B	6H	6D	6VS
Hv_Contig_514_nlr_1	Complete NLR	6D	6H	6D	6VL
Hv_Contig_750_nlr_1	Complete NLR	6B	6H	5D	6VL
Hv_Contig_860_nlr_1	Complete NLR	6A	6H	6D	6VL
Hv_Contig_1012	Partial or pseudogene	6A	6H	6D	6VS
Hv_Contig_1162	Partial or pseudogene	6B	6H	Un	6VS
Hv_Contig_736	Partial or pseudogene	6D	Un	6D	6VS
Hv_Contig_539	Partial or pseudogene	6B	6H	6D	6VL
Hv_Contig_38_nlr_2	Complete NLR	7A	7H	7D	7VS
Hv_Contig_55_nlr_1	Complete NLR	7D	7H	7D	7VS
Hv_Contig_99_nlr_3	Complete NLR	7D	7H	7D	7VL
Hv_Contig_508_nlr_1	Complete NLR	4A	7H	3D	7VL
Hv_Contig_1010_nlr_1	Complete NLR	7B	Un	7D	7VL
Hv_Contig_1236_nlr_1	Complete NLR	7D	7H	7D	7VL
Hv_Contig_632	Partial or pseudogene	7B	7H	7D	7VS
Hv_Contig_912	Partial or pseudogene	7A	7H	7D	7VS
Hv_Contig_414	Partial or pseudogene	4A	7H	7D	7VL
Hv_Contig_940	Partial or pseudogene	7D	7H	7D	7VL
Hv_Contig_978	Partial or pseudogene	7A	4H	7D	7VL

Note: Un indicates that the chromosomal location of the matched gene was unknown

### Enrichment efficiency of NLR loci corresponding to cloned R genes

In the TSLMMHV1 bait design, baits corresponding to all the cloned barley *Mla* genes were added manually. To test the efficiency of enrichment, the orthologous *H. villosa Mla* genes were obtained from our *H. villosa* NLR assembly. In total, 18 NLRs in *H. villosa* were predicted to be orthologous to *Mla* genes because they showed the highest homology to NLRs at the barley *Mla* locus at 30.2 Mb on chromosome 1H (Table 3). When using the rye *Sr50*, the orthologous gene of barley *Mla*, as the query, the same set of homologous genes were obtained. It was reported that the number of *Mla* paralogues was five in wheat and barley but expanded to over 20 in rye [49]. We detected 19 paralogues in *H. villosa* indicating that the expansion of *Mla* genes also occurred in *H. villosa*. In barley, *Mla* paralogues also occur at a second locus at ~ 8.6 Mb on chromosome 1H; we obtained 15 *Mla* paralogues in *H. villosa* corresponding to this locus. The orthologous genes of *Mla* in other species confer resistance to different diseases, such as powdery mildew by *TmMLA1* from *Triticum monococcum* [31], and stem rust by *Sr50* from *Secale cereale* and *Sr33* from *Aegilops tauschii* [49, 56]. Moreover, barley *Mla3*, which confers resistance to barley powdery mildew [1], has recently been shown to also confer resistance to rice blast [10]. Our data suggest that the number of *MLA* paralogues expanded at both sites in *H. villosa* providing rich candidates to mine new resistance genes.

**Table 3:** Orthologous genes in *H. villosa* corresponding to the reported NLR genes

	Contig number	In silico location	Type of NLR	Length of protein (aa)	Identity to reference (%)	Coverage (%)
*Mla1* and *Sr50* (at ~ 30.2 Mb)	Hv_Contig_1079_nlr_1	1 V	CNL	967	86.7	86.0
	Hv_Contig_343_nlr_1	1 V	CNL	960	86.6	86.3
	Hv_Contig_1392_nlr_1	1 V	CNL	955	86.9	86.0
	Hv_Contig_1173_nlr_1	1 V	CNL	949	86.0	88.2
	Hv_Contig_1386_nlr_1	1 V	CNL	954	85.4	84.2
	Hv_Contig_857_nlr_1	1 V	CNL	973	85.0	84.1
	Hv_Contig_60_nlr_2	1 V	CNL	866	87.8	87.9
	Hv_Contig_212_nlr_1	1 V	CNL	951	87.9	86.2
	Hv_Contig_115_nlr_1	1 V	CNL	941	87.4	83.5
	Hv_Contig_653_nlr_1	1 V	CNL	882	87.5	85.9
	Hv_Contig_1211_nlr_1	1 V	CNL	880	86.4	79.3
	Hv_Contig_605_nlr_1	1 V	CNL	886	87.5	79.8
	Hv_Contig_181_nlr_1	1 V	CNL	967	86.6	88.0
	Hv_Contig_1213_nlr_1	1 V	CNL	895	85.1	86.4
	Hv_Contig_436_nlr_1	1 V	CNL	966	85.9	84.7
	Hv_Contig_188_nlr_1	1 V	CNL	913	85.8	80.4
	Hv_Contig_298_nlr_1	1 V	CNL	955	84.7	85.9
	Hv_Contig_650_nlr_1	1 V	CNL	883	84.8	86.0
*Mla1* and *Sr50* (at ~ 8.6 Mb)	Hv_Contig_751_nlr_1	1 V	CNL	921	84.7	78.6
	Hv_Contig_1287_nlr_1	1 V	CNL	932	86.3	67.1
	Hv_Contig_947_nlr_1	1 V	CNL	948	85.7	78.1
	Hv_Contig_678_nlr_2	1 V	CNL	967	85.7	68.1
	Hv_Contig_178_nlr_1	1 V	CNL	967	85.8	87.7
	Hv_Contig_1150_nlr_1	1 V	CNL	937	81.3	82.4
	Hv_Contig_785_nlr_1	1 V	CNL	859	85.8	69.0
	Hv_Contig_399_nlr_1	1 V	CNL	959	87.3	75.8
	Hv_Contig_422_nlr_1	1 V	CNL	943	87.5	78.0
	Hv_Contig_727_nlr_1	1 V	CNL	767	84.4	70.0
	Hv_Contig_559_nlr_1	1 V	CNL	745	80.1	73.5
	Hv_Contig_238_nlr_1	1 V	CNL	964	84.8	63.9
	Hv_Contig_668_nlr_1	1 V	CNL	909	84.5	85.0
	Hv_Contig_119_nlr_2	1 V	CNL	947	80.5	91.1
	Hv_Contig_924_nlr_1	1 V	CNL	947	84.9	84.9
*Pm3b*	Hv_Contig_1461_nlr_1	1 V	CNL	1422	91.5	93.4
	Hv_Contig_1202_nlr_1	1 V	CNL	1525	90.1	100
	Hv_Contig_1215_nlr_1	1 V	CNL	1473	90.4	93.7
	Hv_Contig_1186_nlr_1	1 V	CNL	1485	84.7	96.9
*Sr45*	Hv_Contig_1433_nlr_1	1 V	CNL	1204	91.6	65.2
	Hv_Contig_1007_nlr_1	1 V	CNNL	1156	91.6	61.6
	Hv_Contig_232_nlr_1	1 V	CNL	1219	84.1	73.1
*Yr7*	Hv_Contig_909_nlr_1	2 V	CC-zf-BED-NL	1509	80.0	82.0
	Hv_Contig_430_nlr_1	2 V	zf-BED-NL	1417	74.5	91.3
*RCR1*	Hv_Contig_1205_nlr_1	3 V	CNL	943	87.8	96.6
*Sr35*	Hv_Contig_191_nlr_1	3 V	CNL	922	89.5	67.0
	Hv_Contig_479_nlr_1	3 V	CNL	918	84.3	78.2
*Lr1*	Hv_Contig_1032_nlr_1	5 V	CNL	872	86.3	73.6
	Hv_Contig_1499_nlr_1	5 V	NL	1169	86.1	71.2
	Hv_Contig_582_nlr_1	5 V	CNL	1392	85.3	77.6
*Pm2*	Hv_Contig_335_nlr_1	5 V	CNL	1260	93.0	99.8
	Hv_Contig_1045_nlr_1	5 V	CNL	1230	86.1	89.4
	Hv_Contig_810_nlr_1	5 V	CNL	1053	80.1	86.0

We used several other cloned NLR genes to test the enrichment efficiency. Six NLRs in silico*-*located on 1 V showed highest homology to the *Pm3* locus and as such were predicted to be *H. villosa Pm3* orthologues. Similarly, orthologues for *Sr45*, *Yr7*, *RCR1*, *Sr35*, *Lr1*, and *Pm2* could also be successfully identified in *H. villosa* from 1 V, 2 V, 3 V and 5 V respectively. No orthologues of wheat *Tsn-1*, *Sr22* or *Lr10* were detected on the corresponding chromosomes of *H. villosa*, however, these *R* genes also lack orthologues in barley. Our results indicate that orthologues of cloned wheat *R* genes could be efficiently captured by NLR enrichment, and the data could be used for evolutionary an functional studies of NLR genes at specific loci.

### Identification of NLRs with integrated domains

**Fig. 4:**
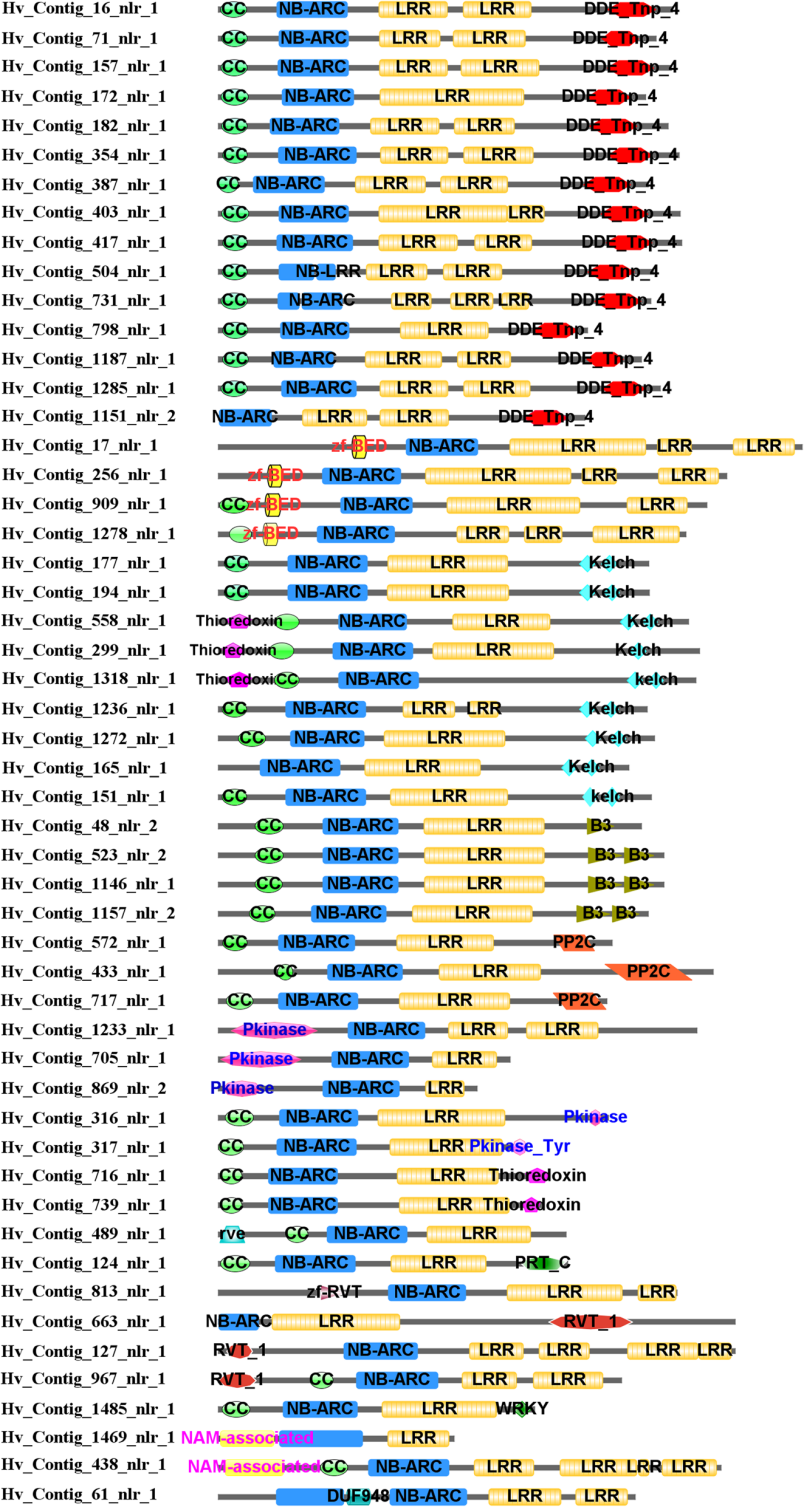
Domain composition of the identified NLR-IDs in *H. villosa*. Besides the CC, NB-ARC and LRR domains, the atypical domains were characterized as integrated domains in 52 annotated NLR-IDs, including DDE_Tnp_4, Kelch repeats, Thioredoxin, Pkinase, zf-BED, B3 DNA binding, PP2C, WRKY, Pkinase_Tyr, RVT_1, rve, zf-RVT, DUF948, NAM-associated, and PRT_C

Atypical domains in NLRs have recently been found to play vital roles in the recognition of pathogens, and these domains can be found in any region of the protein. The additional atypical domains fused into NLRs were designated as integrated decoys (ID), and those proteins with IDs were then classified to be NLR-IDs [12]. Among the 772 determined NLRs of *H. villosa*, a total of 52 NLR-IDs were identified carrying domains other than NB-ARC, CC, and LRR. The identified 15 types of IDs included DDE_Tnp_4, Kelch repeats, Thioredoxin, Pkinase, zf-BED, B3 DNA binding, PP2C, WRKY and others (Fig. 4). It was interesting to find that the DDE, Kelch and PP2C domains preferred to fuse with the LRR-terminal, the Zinc Finger-BED preferred to fuse with the CC-terminal, while the thioredoxin and Pkinase domains could fuse with both terminals (Fig. 4).

As shown by phylogenetic analysis based on all the NLRs in Fig. 2, NLRs integrated with DDE_Tnp_4, the largest NLR-ID group, were clustered into one branch as Clade B. However, NLR-IDs containing different domains were clustered into ‘Clade J’ and ‘Clade G’ indicating that NLRs in these groups appear to have a predisposition to fuse with other proteins. In addition, the region involved in integration was different in that the NLRs in ‘Clade J’ tend to fuse decoys in the LRR-terminal, while the NLRs in Clade G tend to fuse decoys in the CC-terminal.

**Fig. 5:**
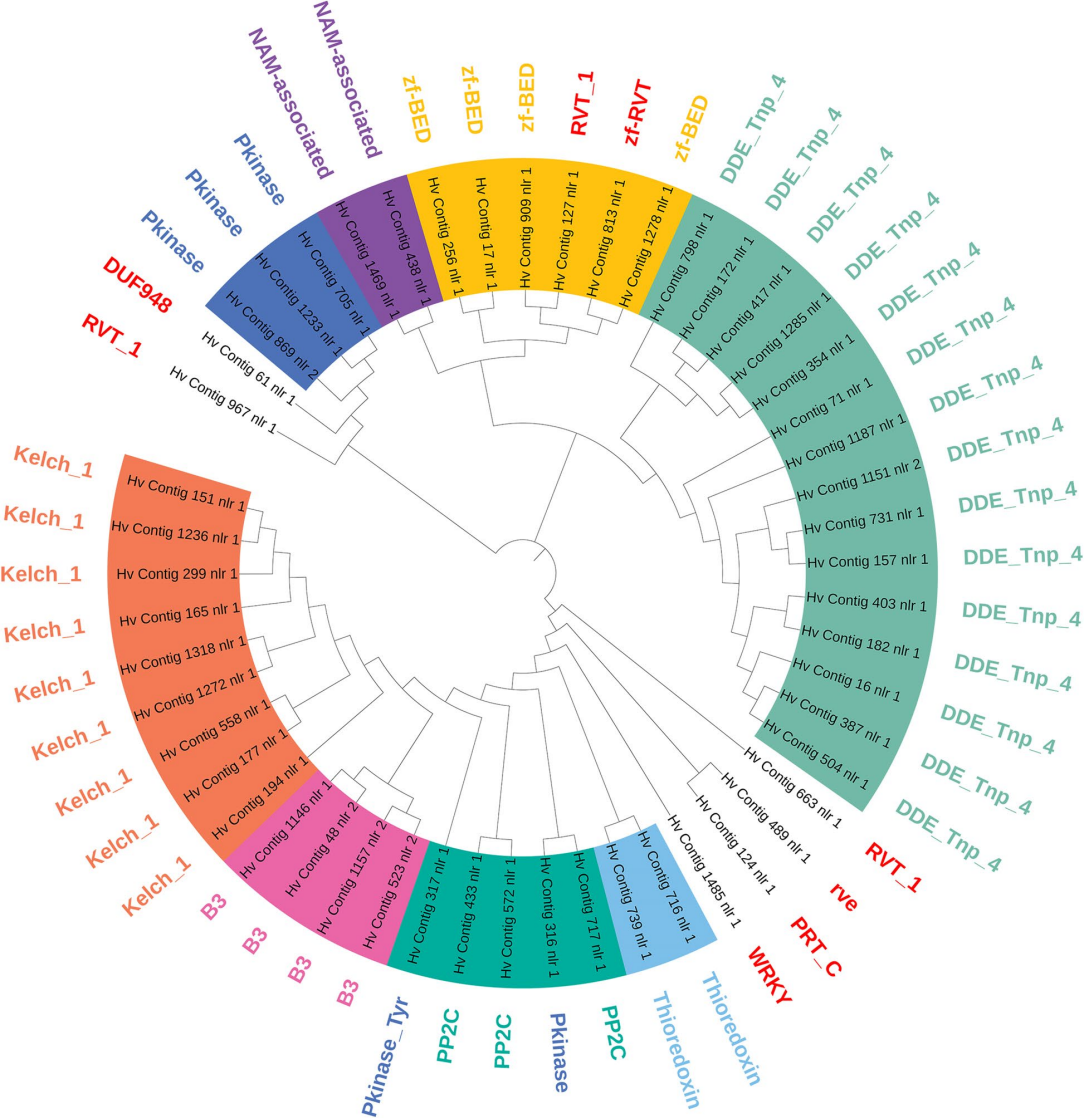
Phylogenetic analysis of the 52 NLR-IDs in *H. villosa*. The phylogenetic tree of the 52 NLR-IDs of *H. villosa* was constructed using MEGA7 by Neighbor-Joining method with 1000 bootstrap replicates, and the tree was visualized using iTOL (https://itol.embl.de/shared/2018201031). Most of NLR-IDs with the same IDs could be phylogenetically clustered, in particular those NLR-IDs containing DDE, B3 and Kelch domains. However, NLR-IDs with Pkinase or RVT_1 were spread across different groups

The phylogenetic analysis using 52 NLR-IDs of *H. villosa* was also conducted as shown in Fig. 5 (https://itol.embl.de/shared/2018201031). Most of the NLR-IDs with the same IDs could be phylogenetically clustered, especially those NLR-IDs containing DDE, B3 and Kelch clustered into large groups, respectively. The tightly clustered NLR-IDs with similar domain compositions in the tree were usually in silico located on the same chromosomes, indicating that these members likely expanded by tandem duplication after the NLR fused with the ID. There are exceptions, for example, NLR-IDs with Pkinase were clustered into different groups indicating independent cases of fusion. The direct evidence is that the tightly grouped NLR-IDs, Hv-contig-705, Hv-contig-869 and Hv-contig-1233, contained a Pkinase integrated into the CC-terminal, while Hv-contig-316 and Hv-contig-317 contained a Pkinase integrated into the LRR-terminal (Figs. 4 and 5). Another example for independent fusion concerns the case of RVT_1 which is found integrated into Hv-contig-967, Hv-contig-127 and Hv-contig-663, which have different domain compositions and chromosome locations (Figs. 4 and 5).

**Fig. 6:**
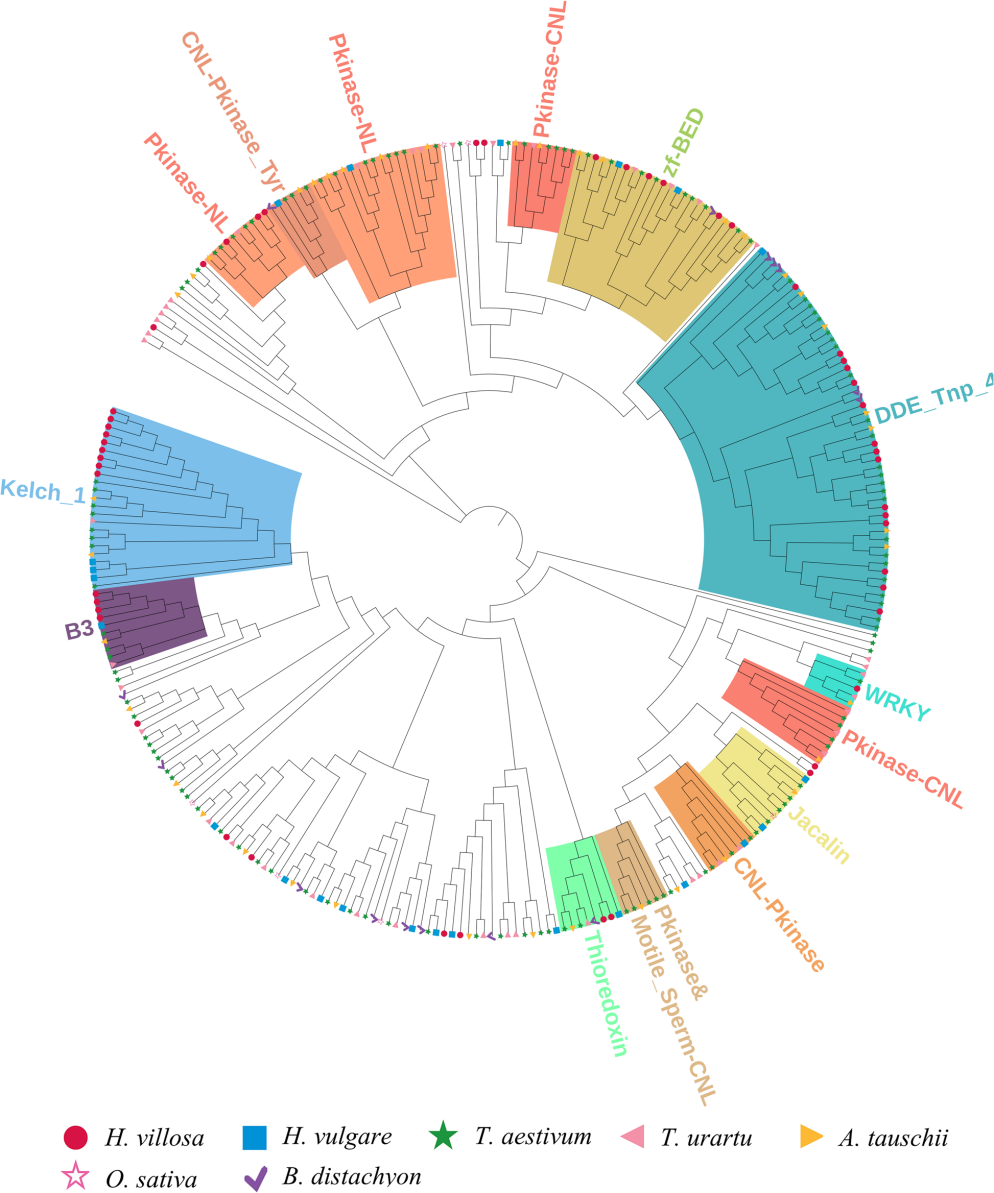
Phylogenetic analysis of the 315 NLR-IDs in seven *Triticeae* species. The phylogenetic tree of the 315 NLR-IDs from seven *Triticeae* species was constructed using MEGA7 by Neighbor-Joining method with 1000 bootstrap replicates, and the tree was visualized using iTOL (https://itol.embl.de/shared/2018201031). *NLR-IDs* carrying Kelch and B3 domains showed a pronounced expansion in *H. villosa*, whereas *NLR-IDs* with Pkinase were less abundant, and those with Jacalin were altogether lacking

The NLR-IDs were extracted from another six *Triticeae* species, then the number of NLR-IDs and the types of IDs were compared in detail. A total of 65 IDs were detected from the seven species (Table 4). The number of shared IDs and specific IDs in each species were as follows, 28 and 5 in *T. aestivum*, 35 and 19 in *T. urartu*, 18 and 2 in *Ae. tauschii*, 19 and 6 in *H. vulgare*, 9 and 0 in *B. distachyon*, and 8 and 3 in *O. sativa* (Table 4). Among the 15 IDs found in *H. villosa*, 12 IDs were shared with at least one species. However, three IDs, including DUF948, NAM-associated and PRT_C, specifially existed in *H. villosa* implying the emergence of these after divergence of *H. villosa*. The phylogenetic tree was also constructed using all the NLR-IDs from these species. It was found that NLR-IDs fused with Kelch and B3 domains expanded dramatically in *H. villosa*, however, NLR-IDs containing Pkinase were fewer in number, whereas those containing Jacalin were altogether lacking in *H. villosa* (Fig. 6, https://itol.embl.de/shared/2018201031).

**Table 4:** The integrated domains identified in seven grass species

Integrated Domain	Description of IDs	Genomes where IDs were detected
Thioredoxin	Thioredoxin	VV, AABBDD, AA, DD, HH, Bd, Os
B3	B3 DNA binding domain	VV, AABBDD, AA, DD, HH, Os
Pkinase	Protein kinase domain	VV, AABBDD, AA, DD, HH, Bd
Pkinase_Tyr	Protein tyrosine and serine/threonine kinase	VV, AABBDD, AA, DD, HH, Bd
WRKY	WRKY DNA-binding domain	VV, AABBDD, AA, DD, HH, Bd
zf-BED	BED zinc finger	VV, AABBDD, AA, DD, HH, Bd
DDE_Tnp_4	DDE superfamily endonuclease	VV, AABBDD, DD, HH, Bd
PP2C	Protein phosphatase 2C	VV, AABBDD, AA, DD, HH
Kelch_1	Kelch motif	VV, AABBDD, AA, DD, HH
RVT_1	Reverse transcriptase	VV, AA
rve	Integrase core domainV	VV, AA
zf-RVT	Zinc-binding in reverse transcriptase	VV, AA
DUF948	Domain of unknown function	VV
NAM-associated	EF-hand domain pair	VV
PRT_C	NPR1/NIM1 like defence protein C terminal	VV
Jacalin	Jacalin-like lectin domain	AABBDD, DD, HH, Bd, Os
DUF761	Cotton fibre expressed protein	AABBDD, DD, Os
Exo70	Exo70 exocyst complex subunit	AABBDD, AA, HH
Motile_Sperm	MSP (major sperm protein) domain	AABBDD, AA, DD
CG-1	CG-1 domains	AABBDD, AA
DUF295	Unknown function	AABBDD, AA
Ank_2	Ankyrin repeats	AABBDD, AA
TB2_DP1_HVA22	TB2/DP1, HVA22 family	AABBDD, DD
DUF296	Plants and prokaryotes conserved (PCC) domain	AABBDD, DD
GRAS	GRAS (GAI, RGA, SCR) family	AABBDD, DD
Myb_DNA-binding	Myb-like DNA-binding domain	AABBDD, Bd
AP2	AP2 domain	AABBDD, Bd
RIP	Ribosome inactivating protein	AABBDD, HH
VQ	VQ motif	AABBDD, Os
AvrRpt-cleavage	Cleavage site for pathogenic type III effector avirulence factor Avr	DD, HH
BPS1	Staphylococcal nuclease homologue	AABBDD
CPSF100_C	Tudor domain	AABBDD
Ceramidase	Bacterial protein of unknown function	AABBDD
TIG	No apical meristem-associated C-terminal domain	AABBDD
zf-RING_2	Phosphoribosyltransferase C-terminal	AABBDD
Aldo_ket_red	AUX/IAA family	AA
BTB	FNIP Repeat	AA
CwfJ_C_1	Glutaredoxin	AA
CwfJ_C_2	Paired amphipathic helix repeat	AA
DUF3420	Zinc-finger of the FCS-type, C2-C2	AA
DUF3615	LSD1 zinc finger	AA
DUF4216	C1 domain	AA
DUF4218	F-box	AA
EF-hand_7	Transport inhibitor response 1 protein domain	AA
NPR1_like_C	Protein BYPASS1-related	AA
PARP	Cleavage and polyadenylation factor 2 C-terminal	AA
PTEN_C2	Ceramidase	AA
RST	IPT/TIG domain	AA
RVT_3	Ring finger domain	AA
Retrotran_gag_2	Aldo/keto reductase family	AA
XH	BTB/POZ domain	AA
gag_pre-integrs	Protein similar to CwfJ C-terminus 1	AA
tRNA_synt_2f	Protein similar to CwfJ C-terminus 2	AA
zf-CCHC_4	Domain of unknown function	AA
SNase	Protein of unknown function	DD
TUDOR	Domain of unknown function	DD
AUX_IAA	Poly (ADP-ribose) polymerase catalytic domain	HH
FNIP	C2 domain of PTEN tumour-suppressor protein	HH
Glutaredoxin	RCD1-SRO-TAF4 (RST) plant domain	HH
PAH	Reverse transcriptase-like	HH
zf-FLZ	Gag-polypeptide of LTR copia-type	HH
zf-LSD1	XH domain	HH
C1_2	GAG-pre-integrase domain	Os
F-box_5	Glycyl-tRNA synthetase beta subunit	Os
Transp_inhibit	Zinc knuckle	Os

Note: *VV* indicates species *Haynaldia villosa*, *AABBDD* indicates *Triticum aestivum*, *AA* indicates *Triticum urartu*, *DD* indicates *Aegilops tauschii*, *HH* indicates *Hordeum vulgare*, *Bd* indicates *Brachypodium distachyon* and *Os* indicates *Oryza sativa*

We found that the homeologues of paired NLRs, such as RPS4/RRS1, Rpg5/RGA1, and RGA4/RGA5, usually displayed a pattern of head-to-head arrangement. *NLR-IDs* with physically tightly linked *NLRs* often form paired protein complexes. In this study, we found that nine NLR-IDs were located tandemly with another NLRs in the same contig, and in two contigs, namely Hv_Contig_48 and Hv_Contig_1157, NLR-IDs and its linked NLRs were arranged in a head-to-head pattern.

## Discussion

### SMRT-RenSeq and NLR-annotator facilitates reference-free genome-wide mining of NLRs in a wild grass species

NLRs display high domain-conservation even across species. Previously, random isolation of NLRs from whole genomes was achieved using homology-based PCR in wide variety of species [8]. In recent years, great progress has been made in whole genome NLR gene discovery in species with reference quality genomes, but less so in the large number of non-sequenced species. RenSeq, however, has greatly promoted reference-free genome-wide identification of NLRs and accelerated the cloning of disease resistance genes efficiently from non-model and non-crop species. The application of RenSeq in *Solanum tuberosum*, a wild potato, indicated that ~ 80% sequence identity is enough for capturing NLR genes using oligonucleotide baits [34]. Previously, we compared *H. villosa* and barley and found a high degree of identity between these two closely related species [11]. We therefore used a barley NLR bait library in this study to capture NLRs from *H. villosa*. The enrichment efficiency analysis using the sequences of cloned *R* genes indicated that the homologous genes in *H. villosa* were successfully enriched. Therefore, the bait library designed based on a species with ample genomic resources could be used to isolate NLRs from an evolutionary closely related species.

Before annotation of the NLRs from the assembled contigs, the redundant contigs were removed using the cutoff value of 90 and 95%. Actually, the same 53 contigs were removed by both parameters. To avoid the situation that a recent duplication or residual heterozygosity perhaps could be ruled out as a source for some of the removed contigs, these 53 removed contigs were reanalyzed (Table S4). It was found that the 22 contigs contained no, partial or pseudogene NLRs, 16 contigs contained complete NLRs with > 99% identity to the already annotated NLRs, and the 15 contigs contained complete NLRs with 95–99% identity to the already annotated NLRs. Finally, the contigs with no, partial, pseudogene NLRs or with complete NLRs showing > 99% identity to the already annotated NLRs were removed. The reannotated 15 complete NLRs showing 95–99% identity to the already annotated NLRs were included in the database along with the previously identified 772 NLRs.

### RenSeq helps to identify more NLRs in *Haynaldia villosa*

NLR complements have been identified in several diploid species related to *H. villosa* using different reference assemblies and different pipelines, such as 463, 570, 563 or 558 NLRs in *T. urartu*, 842 or 738 NLRs in *A. tauschii*, 420, 336 or 462 NLRs in *H. vulgare*, 422 or 341 NLRs in *S. bicolor*, and 470 or 438 in *S. italica* [4, 22, 44, 63]. In this study, using the latest version of the released genomic database, we identified 350 NLRs from *B. distachyon*, 347 from *O. sativa*, 397 from *H. vulgare*, 530 from *T. urartu*, 572 from *A. tauschii* and 772 from *H. villosa* by the same pipeline. *H. villosa* contained relatively more *NLRs* than what has been reported in most diploid genomes. The study in *Solanum tuberosum* indicated that more *NLRs* were revealed by RenSeq than those predicted by the then available annotation software and genome reference suggesting that RenSeq facilitates the recovery of *NLRs* from the poorly or previously unannotated regions of the genome [34]. Moreover, cDNA SMRT-RenSeq, with its longer reads, could help correct splicing errors generated by using short read sequencing technology. The large number of NLRs that we recorded in our study in *H. villosa*, might be accounted for by the NLR expansion that occurred at some loci.

Some of the in silico located NLRs were selected for physical location to specific chromosome arms, and good congruency was found for chromosomes 1 V, 2 V, 3 V, 5 V and 6 V suggesting these NLRs could be used as candidates for mining for R genes genetically assigned to these chromosomes. However, some of the NLRs, which were in silico located on 7 VS were physically located on 4VL. Therefore, NLRs in silico located on 7 VS could be used as candidates for mining for R genes which map to 4VL. Chromosomal rearrangements are commonly found in wheat and its wild relatives [75], and our results will facilitate accurate R gene location and cloning in such chromosome regions.

### NLRs with unique integrated domains are found in *Haynaldia villosa*

In host-pathogen interactions, a decoy is used to describe those molecules which mimic a component manipulated during infection [72]. The decoy strategy is used by both the pathogen to inhibit the host defense and by the host to inhibit the pathogen infection [55]. In the host, decoys are often integrated with NLRs, but decoys can also be physically independent of the NLRs [17]. For example, Pto in tomato mimics the RLKs that are targeted by the effector *AvrPto* injected into the host cell by the bacterial pathogen *Pseudomonas syringae*. The partner of Pto, Prf, an NLR receptor, senses the modification of Pto by AvrPto to trigger immune signaling [51]. Sometimes, decoys, referred to as integrated-decoys, are fused with the N or C terminal of NLRs. For example, RRS1, an integrated-decoy NLR from *A. thaliana*, carries a C-terminal WRKY domain which mimics the WRKY protein targeted by the PopP2 effector. Other examples include the NLRs RGA5 and Pik-1 in rice, which carry a heavy metal associated (HMA) domain targeted by AVR-Pia and AVR-Pik, respectively [13, 53, 82]. Intracellular detection of pathogen-derived molecules through integrated domains in NLRs is a typical resistance pathway among the nine mechanisms identified in hosts to date [36].

Integration of decoy domains in NLRs is frequent in plants. It was found that on average across 31 species analyzed, 3.5% of all NLR proteins are integrated with 90 diverse protein domains, with 2.5% in CNLs and 4.7% in TNLs [37]. However, Sarris [63] predicted that as many as 10% of plant NLRs contain highly variable domains. In the present study, 52 NLR-IDs (about 6.7%) were found among the 772 NLRs in *H. villosa*. While the NLR-ID ratio was smaller in *H. villosa* compared to *T. urartu* and *Ae. tauschii*, the total number of NLR-IDs in *H. villosa* was the largest among all the seven species studied (Table 4). Except for *B. distachyon*, unique IDs were detected in each species, indicating that novel fusions happened after species divergence. The most cases were observed in *T. urartu* harboring 19 distinct IDs, while three cases happened in *H. villosa* harboring three distinct IDs. We also found ‘Clade J’ and ‘Clade G’ of *H. villosa* appear to have a predisposition to fuse with other proteins, and a major integration clade whose members underwent repeated independent integration events was also described [4].

The decoys integrated in NLRs are usually the duplicated products of effector targets. Protein kinases, transcription factors and proteases, which have been reported to be effector targets previously, were found to be fused as decoys in NLRs with high frequency. Therefore, genome-wide mining of NLR-IDs can help identify putative effector targets. The integrated domains identified here in *H. villosa* include DDE_Tnp_4, kelch repeats, thioredoxin, protein kinase, zinc finger-BED, B3 DNA binding, integrase and PP2C. These protein families are therefore considered as likely effector targets.

Previous studies have found that NLR-IDs usually work in pairs and link proximally in genome. For example, RPS4 works in concert with RRS1 to confer resistance to *P. syringae* [53]*,* RPP2A pairs with RPP2B to confer resistance to *Hyaloperonospora arabidopsidis* [65], Rpg5 pairs with RGA1 to confer resistance to *P. tritici* [74]*,* while Pikh-1 with Pikh-2 [82] and Pi5–1 with Pi5–2 [39] to confer resistance to *M. oryzae*. Therefore, the ability to detect tightly-linked NLRs in genomes can provide important clues as to their modus operandi. In this study, we found nine NLRs-ID located physically proximal to another NLR in the same contig, among which two cases were arranged in a head-to-head orientation. However, NLR-IDs seem not to be physically paired NLRs at the DNA sequence level which was also predicted from a study in rice [37].

### Genome-wide exploration and chromosomal location of NLRs is critical for cloning and introducing new resistance genes from *H. villosa*

Wild crop relatives represent an important reservoir of disease resistance genes, which in some cases can be introduced into cultivated crops by wide crosses. Genetically diverse accessions of *H. villosa* has been configured and shown to display variation in disease resistance [14, 16, 26, 43, 52, 59, 83–85]. However, as is the case with most wild species, gene utilization is inefficient. In this study, the genome-wide identification of NLRs from *H. villosa* and mapping of NLRs to each chromosome arm of *H. villosa* will change the way of introducing new resistances into wheat. Firstly, the public availability of all the NLRs provides a precious resource for accelerating the cloning of resistance genes, which will help to introduce new resistances into wheat through gene transformation. Secondly, the information of NLRs sequences and their chromosome locations provides important data for different research groups to compare the NLRs sequences located on specific chromosome or even on specific chromosome region between different *H. villosa* accessions, which could help researchers identify new resistance germplasm and transferring new resistance genes or alleles of *H.villosa* into wheat. Furthermore, the availability of all the NLRs facilitates developing molecular markers specific to *H.villosa* based on the NLRs, so it could help transfer chromatin containing putative resistance genes of *H.villosa* into wheat by molecular marker assistant selection and help accelerate developing new resistance germplasms with small segments introgression.

Previously, the SMRT-RenSeq data of *H. villosa* accession 91C43 helped us to clone the broad-spectrum powdery mildew resistance gene *Pm21* [79]. Following this, alleles of *Pm21* were cloned by homology-based PCR from more than 100 accessions. The high genetic diversity between these alleles provides valuable information to understand the structure, function and evolution of *Pm21*.

NLRs are subject to intensive diversifying selection due to their genetic interaction with rapidly evolving pests and pathogens [23, 64]. This process may lead to the production of large amounts of pseudogenes. In *H. villosa*, besides the 772 complete NLRs, we detected another 289 NLRs which were classified as pseudogenes. These were widely spread across the genome and may be the remnants of past evolutionary activity. In the hexaploid wheat cultivar Chinese Spring, there are 1540 complete NLRs and 2360 putative pseudogene NLRs [69]. Pseudogenes may retain useful evolutionary potential through the propensity of NLRs for intergenic recombination. For example, a functional chimeric allele of the leaf rust resistance gene *Lr21* was recovered from a cross between two non-functional alleles [24]. Moreover, a locus containing a pseudogene in one accession may harbor a functional allele in another accession [47]. For example, the *Pm2* allele in Chinese spring contains a premature stop codon, while in cultivar Ulka the open reading frame is retained giving rise to a functional resistance gene [62, 69]. Therefore, the pseudogenes identified in *H. villosa* may provide valuable clues for mining of functional alleles in other accessions.

## Conclusions

In this study, the genome-wide *NLR* complement of *H. villosa* was efficiently identified using SMRT-RenSeq, and a total of 772 complete NLRs were annotated. The information of the chromosome location of all the *NLRs* were provided, which is valuable for resistance gene mining from the specific chromosome. The physical location of *NLRs* from group 1, 2, 3, 5 and 6 showed a perfect homoeologous relationship with other *Triticeae* species except the *NLRs* on chromosome 4VL which were predicted to be located on the homoeologous group 7 in silico. Cluster expansion observed in some specific gene loci indicated that independent evolutionary cases occurred in *H. villosa*. We also identified 52 NLR-IDs with fifteen types of integrated domains (IDs), among which Kelch and B3-type NLR-IDs experienced expansion, and three type of IDs were unique in *H. villosa*. This study gave an example to successfully capture the genome-wide *NLRs* in wild species using the baits from another species in *Triticeae*. The availability of the NLRs from *H. villosa* provides a valuable library for mining and transferring of disease resistance into wheat.

## Materials and methods

### NLR-enrichment library construction and physical mapping of NLR genes

*Haynaldia villosa* (genome constitution VV, 2n = 14), with long hairs on the keel of the glume and apex of the lemma, is an annual diploid species which belonging to *Triticinae* of *Triticeae* in *Gramineae*. It is a vigorous ruderal wild plant growing on the harsh, moisture-stressed soils in the northeastern part of the Mediterranean region and Caucasus area [20]. This species usually confers resistance to different diseases and tolerance to various abiotic stresses [20]. The *H. villosa* accession 91C43 was obtained from Cambridge University, UK and propagated by artificial bagging and self-pollination for 6–7 successive generations to obtain an inbred line. Tissue from the 91C43 inbred line was used for DNA extraction. This accession was also used as the donor to develop 14 wheat-*H.villosa* translocation lines through wide-crosses and chromosome engineering, each involving one of the 14 chromosome arms of *H. villosa*, respectively [86]. This set of translocation lines was used for chromosomal location of NLRs.

### Library construction for NLR gene enrichment and SMRT sequencing

The barley NLR bait library TSLMMHV1 [9] was used to capture the NLR complement from *H. villosa* accession 91C43. The design of this NLR bait library has been previously described [9]. In brief, NLR-Parser [ 67], a tool for high-throughput identification of NLRs based on conserved motifs [33], was used to search eight barley tanscriptomes and six barley genomes [25] (IBGSC2012) to identify those sequences containing at least one CC and two NBS motifs, or two NBS and one LRR motifs [9]. Previously-reported barley disease resistance genes, including *Mla*, *Mlo*, *Rpg1*, etc., were manually added. Repetitive and redundent sequences were removed to yield the final set of 99,421,100-mer RNA baits.

Genomic DNA for SMRT library preparation was extracted from seedlings of *H. villosa* with the DNeasy Plant Mini Kit (Qiagen, Hilden, Germany). SMRT library preparation and NLR capture followed the procedure described by Witek et al. [76]. The enriched library was sequenced on the PacBio RSII platform using four SMRT cells in the Genome Analysis Center (TGAC, Norwich Research Park, UK) with P4-C6 chemistry to minimize errors.

### Assembly of the SMRT-RenSeq reads

The raw reads were screened by the SMRT Portal software with parameter settings > 3 full passes and > 90% accuracy to generate the inserted sequence ROI. The long ROI were analyzed and assembled using genetic R8 software (www.geneious.com) with defaults of 1% mismatch, 1% gap (no more than 3 bp) and minimum read length overlap greater than 100 nt with at least 98% identity. Only contigs assembled from at least five sequences and having a minimum coverage of two were considered for further analysis. A total of 1509 contigs fulfilling these criteria were obtained.

### Sequence annotation of the assembled SMRT-RenSeq contigs by NLR-annotator

To obtain non-redundant NLR-containing contigs, the raw assembled contigs were analysed by the global alignment program Cd-hit (version 4.8.1) [42] to construct a phylogenetic tree and remove the sequences with > 95% identity. The retained contigs were analysed with RepeatMasker followed by screening for NLRs using NLR-Annotator (https://github.com/steuernb/NLR-Annotator).

### Extraction of protein sequences from the annotated NLRs

The predicted NLR loci were extended backwards by 3000 bp from the left border and forwards by 3000 bp from the right border, respectively. The extended sequences were cut out for BLASTx against the database of barley (ftp://ftp.ensemblgenomes.org/pub/plants/release-46/fasta/hordeum_vulgare), *Aegilops tauchii* (ftp://ftp.ensemblgenomes.org/pub/plants/release-46/fasta/aegilops_tauschii), and common wheat (ftp://ftp.ensemblgenomes.org/pub/plants/release-46/fasta/triticum_aestivum) to find the orthologous genes from the three species. In each species, the proteins with the highest identity and longest coverage were selected as the putative orthologous proteins. The protein sequence from the species with the highest identity and longest coverage compared with *H. villosa* was used as the reference to predict the coding sequence of the corresponding *H. villosa* NLR using FGENESH+ (http://www.softberry.com/).

### Domain composition analysis of the annotated NLRs

The protein sequences of six species, including *T. aestivum*, *Ae. tauschii*, *T. urartu*, *H. vulgare*, *O. sativa*, and *B. distachyon*, were downloaded from Ensembl Plants (http://plants.ensembl.org/info/website/ftp/index.html) and the longest proteins corresponding to the longest transcripts of genes with alternative splicing were used for annotation analysis (Table S1). Plant_rgene (https://github.com/krasileva/plant_rgenes) and InterProScan (interproscan-version 5.30–69.0) were used to analyze the NLRs annotated as being complete. Plant_rgene program is a pipeline for analyzing the domain composition of putative NLRs, however, sometimes it cannot accurately output LRR domains. The InterProScan software [30] is a complementary tool for identification of the LRR domains deposited in the SUPERFAMILY database. Therefore, all the proteins, including the 774 complete *H. villosa* NLRs and the proteomes from the six species listed above, were analyzed by the Plant_rgene software (evalue = 1e-3) to search for conserved domains (including CC, NB-ARC and integrated domains). Then the same set of proteins were analyzed again by InterProScan to search for the conserved LRR domains deposited in the SUPERFAMILY database. The conserved domains output from both programs were integrated. Only those proteins containing an NB-ARC domain were designated as NLRs. If any atypical domain was additionally predicted in the NLRs, these domains were considered to be the integrated-decoys (IDs), and those NLRs containing ID(s) were then designated as NLR-IDs. The diagrams of all the 52 NLR-IDs were displayed by IBS (Version 1.0.3) [45].

### Phylogenetic tree construction

For construction of the phylogenetic tree of all the 772 complete NLRs, the conserved NB-ARC domain was extracted from each NLR and a phylogenetic tree was constructed using MEGA7 by Neighbor-Joining method with 1000 bootstrap replicates [38]. The tree was visualized using iTOL [40]. The conserved motif was searched using MAST (version 4.9.1) [5] based on the ‘motif 1’ to ‘motif 20’ defined by Jupe [33], and displayed in the phylogenetic tree. The phylogenetic tree of the 52 detected NLR-IDs in *H. villosa* and all the 315 NLR-IDs from seven *Triticeae* species were also constructed by the same method using the same parameters.

### Identification of the orthologous NLR genes corresponding to cloned *R* genes

The cloned *R* genes, including *Mla1* (AY009939.1), *Sr50* (KT725812.1), *Sr35* (KC573058.1), *Pm3b* (AY325736.1), *RCR1* (KU161103.1), *Lr1* (EF439840.1) and *Yr7* (MN273771.1) were downloaded from the NCBI database, then the nucleotide sequences were used to perform a sequence alignment with the assembled SMRT RenSeq contigs. The sequences showing > 80% identity and > 60% coverage with the cloned R genes were screened from the annotated NLRs. Then the screened NLRs in silico located to the same homoeologous chromosome loci as the corresponding queries were considered as their putative orthologues respectively.

### The chromosomal location of NLRs

The nucleotide sequences of the identified NLRs were used as query for BLASTn analysis against the genome databases of barley (ftp://ftp.ensemblgenomes.org/pub/plants/release-46/fasta/hordeum_vulgare), *Aegilops tauchii* (ftp://ftp.ensemblgenomes.org/pub/plants/release-46/fasta/aegilops_tauschii), *Triticum urartu* (ftp://ftp.ensemblgenomes.org/pub/plants/release-46/fasta/triticum_urartu) and common wheat (ftp://ftp.ensemblgenomes.org/pub/plants/release-46/fasta/triticum_aestivum) to locate NLRs to chromosomes. Then, a subset of the NLRs were selected to determine the physical location by PCR analysis using 14 wheat-*H. villosa* translocation lines each involving one of the 14 chromosome arms of *H. villosa*. The PCR primers were designed according to specific deletions or insertions in the *H. villosa* NLRs when compared to the presumed orthologous genes in Chinese Spring (Table S2). The PCR procedure was as follows: incubation at 94 °C for 3 min followed by 33 cycles of 94 °C for 45 s, 55 °C–60 °C for 45 s, and 72 °C for 1 min. The PCR products were separated by polyacrylamide gel electrophoresis (Acrylamide:Bisacrylamide = 39:1) followed by silver nitrate staining.

### PacBio transcriptome sequencing of *H. villosa* and NLR expression analysis

PacBio sequencing of the full-length transcriptome extracted from ten mixed tissues of *H. villosa* accession 91C43 was performed by Novogene (Beijing). The mixed tissue sample included seedling leaves, seedling stems, seedling roots, immature spikes, seedling leaves inoculated with *Bgt* for 6 and 24 h, seedling leaves and roots treated with 200 mM NaCl for 3 days, and seedling leaves and roots treated with 16% PEG4000 for 3 days. The PacBio trasncriptome sequences were screened by NLR-Annotator. Then the annotated NLRs of SMRT RenSeq were used to search against the annotated transcribed NLRs. When an NLR from SMRT RenSeq could find a corresponding transcribed NLR with an identity higher than 95% and coverage longer than 90%, then the enriched NLR was considered to be expressed. The sequences of all the expressed NLRs were compared with their genomic counterparts one by one to analyze the accuracy of the protein prediction based on the genomic sequence.

## Supplementary Information

**Additional file 1.**

**Additional file 2.**

**Additional file 3.**

**Additional file 4.**

**Additional file 5.**

**Additional file 6.**

**Additional file 7.**

**Additional file 8.**

## Data Availability

The *H. villosa* SMRT-RenSeq data is available from NCBI under study number SRP132107(https://www.ncbi.nlm.nih.gov/sra/?term=SRP132107). The data of coding sequences and the protein sequences of the annotated NLRs are available in NCBI Genebank database with accession number MZ672249-MZ673036. The data of the full-length transcriptome of *H. villosa* with PacBio SMRT chemistry is available in NCBI SRA database with the accession number SRR15206288 (https://www.ncbi.nlm.nih.gov/sra/SRR15206288). The protein data used for NLR identification from different species were provided in the supplementary information file Table S1, which were obtained from Ensembl Plants database, including: *Aegilops tauschii* (ftp://ftp.ensemblgenomes.org/pub/plants/release-46/fasta/aegilops_tauschii/pep/), *Brachypodium distachyon* (ftp://ftp.ensemblgenomes.org/pub/plants/release-46/fasta/brachypodium_distachyon/pep/), *Hordeum vulgare* (ftp://ftp.ensemblgenomes.org/pub/plants/release-46/fasta/hordeum_vulgare/pep/), *Oryza sativa* (ftp://ftp.ensemblgenomes.org/pub/plants/release-46/fasta/oryza_sativa/pep/), *Triticum aestivum* (ftp://ftp.ensemblgenomes.org/pub/plants/release-46/fasta/triticum_aestivum/pep/) and *Triticum urartu* (ftp://ftp.ensemblgenomes.org/pub/plants/release-46/fasta/triticum_urartu/pep/)*.* In addition, the genomic data used to help identify and chromosomal locate of NLR from *H.villosa* were avalible in Ensembl Plants database, including *Aegilops tauschii* (ftp://ftp.ensemblgenomes.org/pub/plants/release-46/fasta/aegilops_tauschii/dna/), *Hordeum vulgare* (ftp://ftp.ensemblgenomes.org/pub/plants/release-46/fasta/hordeum_vulgare/dna/) and *Triticum aestivum* (ftp://ftp.ensemblgenomes.org/pub/plants/release-46/fasta/triticum_aestivum/dna/).

## Abbreviations

NLR: Nucleotide-binding and leucine-rich repeat
SMRT: PacBio single-molecule real-time
RenSeq: Resistance gene enrichment sequencing
IDs: Integrated domains
NLR-IDs: NLR with integrated domains
CC: Coiled-coil domain
NB-ARC: NB-ARC domain
LRR: Leucine-rich-repeat domain
PAMPs: Pathogen associated molecular patterns
PRRs: Pattern-recognition receptors
PTI: PAMP-triggered immunity
ETI: Effector-triggered immunity
