# Supplementary material for: Genome-wide identification of the NLR gene family in Haynaldia villosa by SMRT-RenSeq
Source: BMC Genomics. 2022 Feb 10;23:118. doi: 10.1186/s12864-022-08334-w (PMC8832786; doi:10.1186/s12864-022-08334-w)
Supplement: Supplementary file 1 — Additional file 1. [file 12864_2022_8334_MOESM1_ESM.pdf]

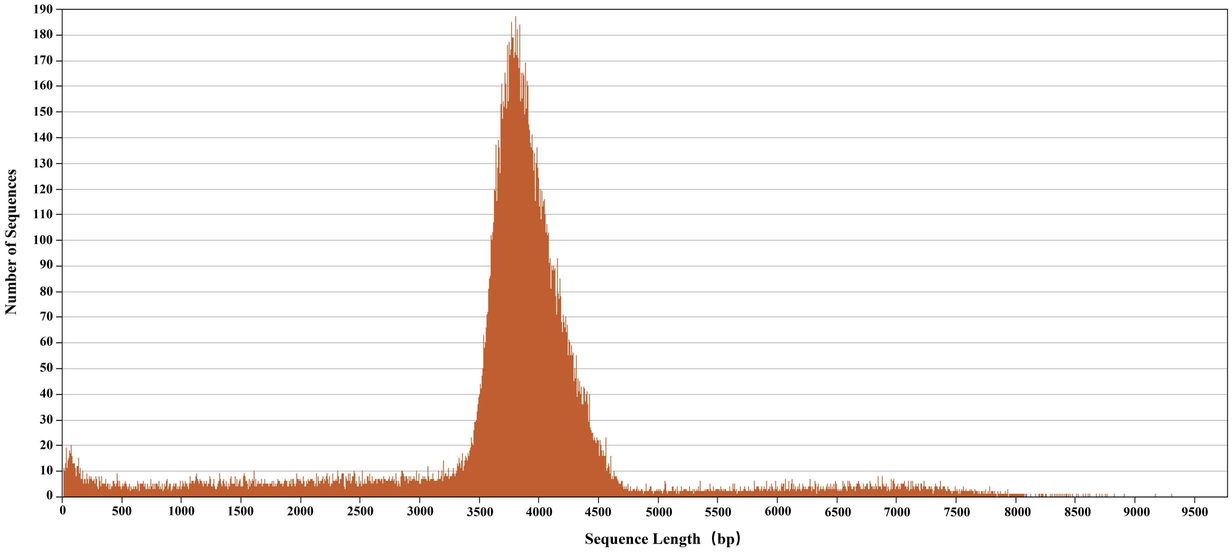

Additional file 1: Fig. S1. Size distribution of the *H. villosa* SMRT-RenSeq raw reads of Insert (ROI).

The size distribution of the 107,153 raw reads produced by SMRT-RenSeq data were analyzed, and the average length of the raw reads was about 4.5 kb.
