# Supplementary material for: Genome-wide identification of the NLR gene family in Haynaldia villosa by SMRT-RenSeq
Source: BMC Genomics. 2022 Feb 10;23:118. doi: 10.1186/s12864-022-08334-w (PMC8832786; doi:10.1186/s12864-022-08334-w)
Supplement: Supplementary file 2 — Additional file 2. [file 12864_2022_8334_MOESM2_ESM.pdf]

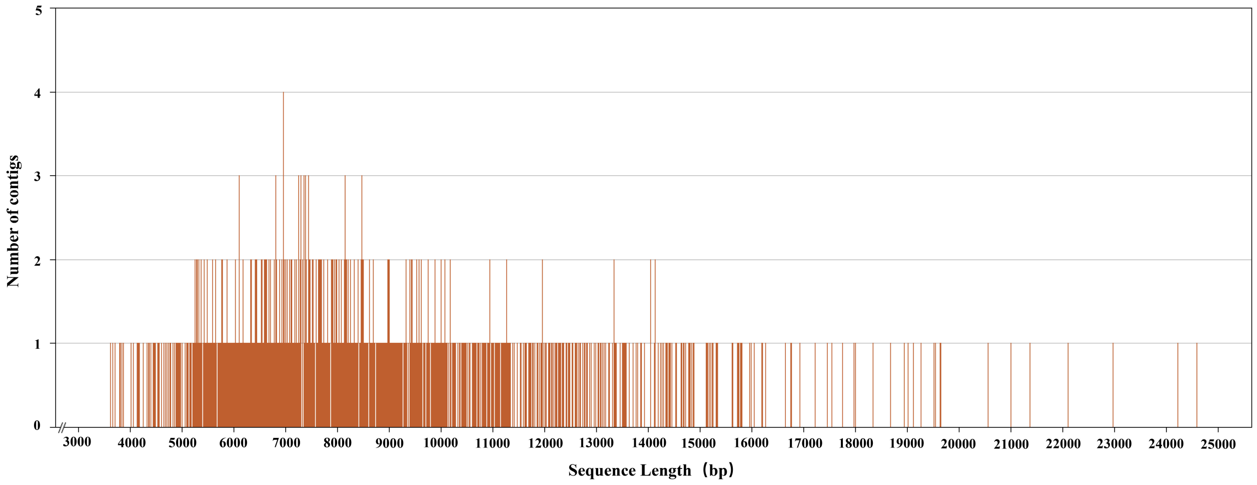

Additional file 2: Fig. S2. Size distribution of 1509 *H. villosa* SMRT-RenSeq assemblies.

The size distribution of the 1509 assembled contigs were analyzed, and 80% of the contigs ranged from 5 kb to 11 kb, with the largest one spanning 24.6 kb.
