# Supplementary figures and images for: Genome-wide identification of the NLR gene family in Haynaldia villosa by SMRT-RenSeq
Source: BMC Genomics. 2022 Feb 10;23:118. doi: 10.1186/s12864-022-08334-w (PMC8832786; doi:10.1186/s12864-022-08334-w)

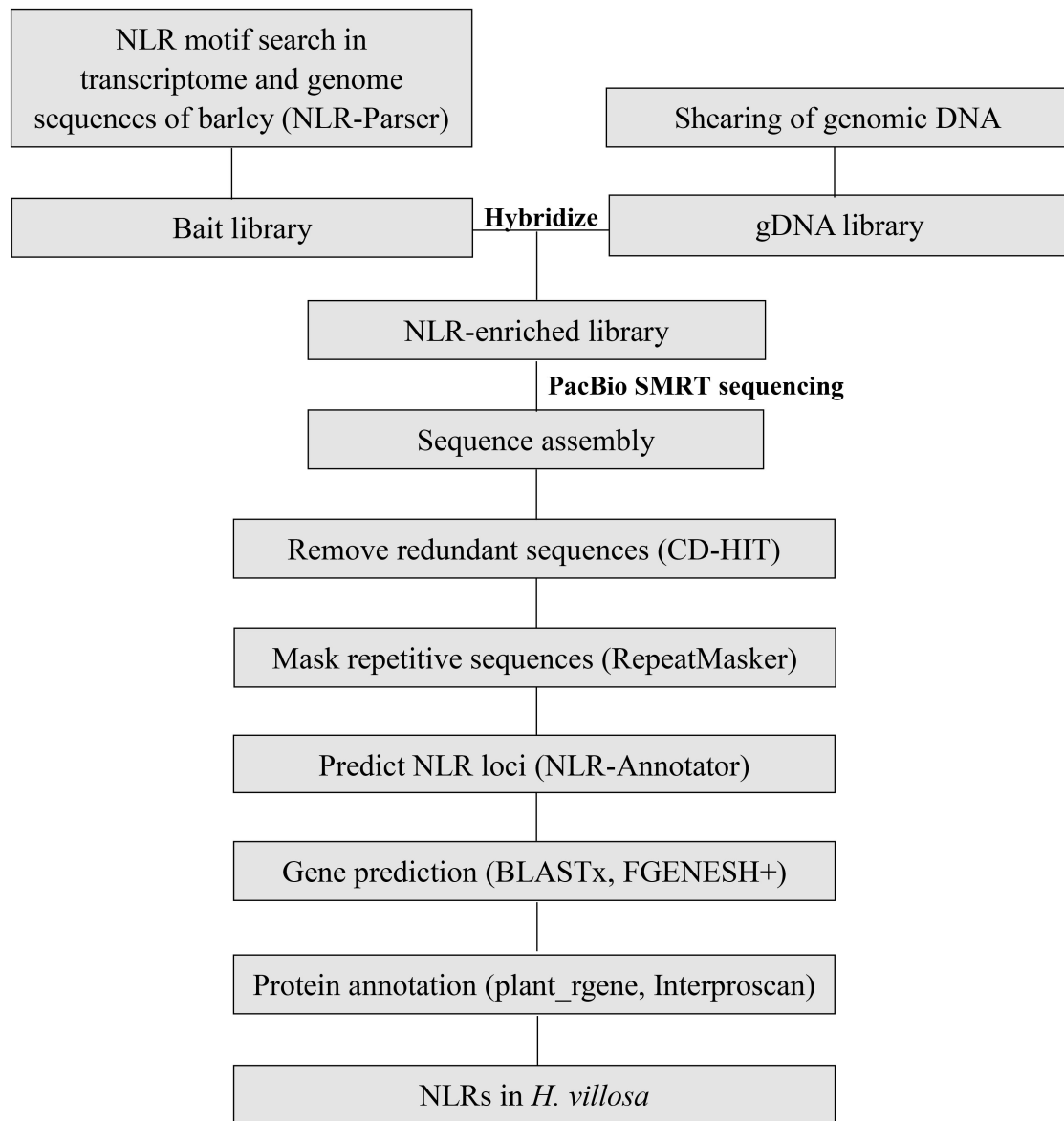

**Additional file 3: Fig. S3.** Pipeline for the identification and annotation of *NLRs* in *H. villosa*.

Supplement: Supplementary file 3 — Additional file 3. [file 12864_2022_8334_MOESM3_ESM.pdf]
