# Supplementary material for: Genome-wide identification of the NLR gene family in Haynaldia villosa by SMRT-RenSeq
Source: BMC Genomics. 2022 Feb 10;23:118. doi: 10.1186/s12864-022-08334-w (PMC8832786; doi:10.1186/s12864-022-08334-w)
Supplement: Supplementary file 4 — Additional file 4. [file 12864_2022_8334_MOESM4_ESM.docx]

| **Organism** | **Accession** | **Genome Assembly** | **Protein** | **Database** | **Data source** |
| --- | --- | --- | --- | --- | --- |
| *Aegilops tauschii* | AL8/78 | Aet_v4.0.pep.all.fa.gz | Aegilops_tauschii.Aet_v4.0.pep.all.fa.gz | Ensembl plants | University of California, Davis |
| *Brachypodium distachyon* | Bd21 | Brachypodium_distachyon_v3.0.pep.all.fa | Brachypodium_distachyon_v3.0.pep.all.fa | Ensembl plants | Joint Genome Institute |
|  |  |  |  |  |  |
| *Hordeum vulgare* | Morex | IBSC_v2.pep.all.fa.gz | Hordeum_vulgare.IBSC_v2.pep.all.fa.gz | Ensembl plants | International Barley Genome Sequencing Consortium |
| *Oryza sativa* | Nipponbare | IRGSP-1.0.pep.all.fa.gz | Oryza_sativa.IRGSP-1.0.pep.all.fa.gz | Ensembl plants | RAP-DB |
| *Triticum aestivum* | Chinese Spring | IWGSC.pep.all.fa.gz | Triticum_aestivum.IWGSC.pep.all.fa.gz | Ensembl plants | International Wheat Genome Sequencing Consortium |
|  |  |  |  |  |  |
| *Triticum urartu* | G1812 | ASM34745v1.pep.all.fa.gz | Triticum_urartu.ASM34745v1.pep.all.fa.gz | Ensembl plants | Beijing Genomics Institute |

**Table S1.** Database used for NLR and NLR-ID identification.
