# Supplementary material for: Genome-wide identification of the NLR gene family in Haynaldia villosa by SMRT-RenSeq
Source: BMC Genomics. 2022 Feb 10;23:118. doi: 10.1186/s12864-022-08334-w (PMC8832786; doi:10.1186/s12864-022-08334-w)
Supplement: Supplementary file 5 — Additional file 5. [file 12864_2022_8334_MOESM5_ESM.docx]

**Table S2.** Primers for chromosomal location of the identified NLRs in *H. villosa*.

| **No. of Contig** | **Primers (5’-3’)** | **Primers (5’-3’)** |
| --- | --- | --- |
| Hv_Contig_60_nlr_3 | GCCTCCTGTACATCTCCGTG | CCAGGCTGAACCCAACTTCT |
| Hv_Contig_138_nlr_2 | CAGCTTTGGCAATGCACCTT | GGACCTAGGGAGCCTGATCA |
| Hv_Contig_140_nlr_1 | ATGTGCTTGTAGCGGTTCCA | TCGAAGCCAACCTGCAAGAT |
| Hv_Contig_232_nlr_1 | GGGCGGAGTGAAACTTTGCTC | CAAATCCCTCGCAGAGAATG |
| Hv_Contig_443_nlr_1 | TCTAATCTCACAGATGTCTAGCAA | TTCAATCGGACTGTAAGCCAT |
| Hv_Contig_992_nlr_1 | CGGTCTCTTGTAATAACAGCAT | CCCGATTGGTATCTTTCTCTGA |
| Hv_Contig_1391_nlr_1 | GGTATCTTCCACCCATTGAA | GCTTTAAGAAAACGGACATACTG |
| Hv_Contig_254_nlr_1 | TTGGCATCTTTATCCGAGAG | TATTCCAACACTGGTCATCTG |
| Hv_Contig_452_nlr_1 | GCTGCAGACACCAACAGTTG | CAGCTCTCGAGTTGCATTGC |
| Hv_Contig_1453_nlr_1 | CCGAGATGCTATCCACCACC | ATCCAGCTCACTGCAGACAC |
| Hv_Contig_951 | ACTCCTCTGTGCGTGTGATG | CCAAGCGATTCGATGCCATG |
| Hv_Contig_1193 | TGTGCTGAACGGAGTGATCC | TGAGGTGGTAGCGCATGTTT |
| Hv_Contig_141 | TGTTCAAGGAAGGACAGGCC | GGACACATGCTGCACTCTCT |
| Hv_Contig_219 | ACTAACCGGCAGATGCAGAG | GGCAAAAGGCTTCACGGAAG |
| Hv_Contig_512_nlr_1 | TTCAGATCGATCGCCTGACG | CGGATCGCCCTTAGATTGCT |
| Hv_Contig_544_nlr_1 | TGGGGCGAAGAACAACTCAT | TAGACACCACCAGCTTGCTG |
| Hv_Contig_223_nlr_1 | GGTCAGACCTGCTGCTACAG | ACCAGCCTTTGTCAGCTTGT |
| Hv_Contig_1028_nlr_1 | CATGGTCCGACTCAAGACAAC | GGACTTAACTGGTTGTTGGCT |
| Hv_Contig_461_nlr_1 | AAGCCAGAACGCCTTGTCAT | TTCAGTGAAGTGAGGCTAACTCG |
| Hv_Contig_1254 | CAGTGAAGAATGGGAGACCG | TCATGCATGACATACCGTGG |
| Hv_Contig_35 | GGATACAGTGGTGCCATCCC | TGCCCAGCGTTGAAAGAAGA |
| Hv_Contig_139 | GCAACCTCTACCTCAACGCT | GCACACACAGCACATGACTG |
| Hv_Contig_667 | TATCCCCATCCCGTCTCCTC | GCATTGGGCGATGAAAGCAA |
| Hv_Contig_716_nlr_1 | ATTCTCCGCATGTCCACGAG | CGACAGCTCCAAGGGAAGTT |
| Hv_Contig_782_nlr_1 | CGAGAGGGCTTCACGAACTT | GCTGAACGTGAAGACCTGGA |
| Hv_Contig_11_nlr_1 | CAAGAAGAGTCCCGGAAGCC | TCATCTGCTCCAAACTGCGA |
| Hv_Contig_79_nlr_1 | TGAGGTACCTGGGATTGTGGA | CTCAGCATTGTCAGCTTGCC |
| Hv_Contig_326_nlr_1 | CCCATGACAGTTGTTTCGGG | GTGTGCTCAATCTACCCGCT |
| Hv_Contig_657_nlr_1 | CCAGTGACGGAAACTCTCCC | TCACTGATTGTGCCGCTCTT |
| Hv_Contig_866_nlr_1 | TGAGGACACCCTTTCTCGTAA | GTTTCCTTTGAAATAACGCTTG |
| Hv_Contig_77 | ACAATGAGCCGCTCTTTCCA | GGCCATGACTCTGTTGAGCA |
| Hv_Contig_686 | CTTGGCACTTCAACACCACG | ACTGGAAGGGGGATCAGTGT |
| Hv_Contig_90_nlr_1 | GGTTGGGAAAGGAGTCTGCA | TAGTGCTGCTGACTGTGCAA |
| Hv_Contig_116_nlr_1 | CATCAATGCTCGCCACACAG | ACTTGGGTAGGGATACGGCT |
| Hv_Contig_322_nlr_2 | TCAAAAAGGCGGGGACAGAA | CTCGAGCCCAACATCACGTA |
| Hv_Contig_670_nlr_1 | TCCCGGTAGACTACCCCATG | CCGGCGAACATCCTTATCCA |
| Hv_Contig_28_nlr_1 | GCACAAGAAAGAAAGAAATATAACC | GTTCAAAGACATCCATAATACAGC |
| Hv_Contig_55_nlr_1 | TGTGGAGGAATTCCCTCATG | CCATTCATATCATCGGGCTAG |
| Hv_Contig_82_nlr_2 | GCAAAGTGCAAACTAGTAGCC | CCAAGGGTGCAGTAAACAGTA |
| Hv_Contig_172_nlr_1 | AGCCACTGCAGAAATCTCGA | TGTTCCACGCAAGAGTCCTC |
| Hv_Contig_299_nlr_1 | CTGCCTTGACTACCCTTGGG | GCTTCAGTACATGCGTGTCG |
| Hv_Contig_393_nlr_1 | ACAACGGGGAAGATTCGACG | CGGAACCCTAGCGTGAAGAA |
| Hv_Contig_648_nlr_1 | TTCCAATGTAAGCATGAGCTGA | TCACCTCTTGTTCTTGTTCTGCT |
| Hv_Contig_798_nlr_1 | GTAGGGGAGCTGACACAACC | TGGTGCAGTTTGGACGAGTT |
| Hv_Contig_913_nlr_1 | GGATGGCCGTGTATTCTGGT | AAACGAGAACGATGCGCTTG |
| Hv_Contig_958_nlr_1 | GAAGCAGACTCGAGATGCCA | TGGAAGAGGGTGCTAGTGGA |
| Hv_Contig_1239_nlr_1 | TGTCCAGGAGGCGATTGAAC | AATCCTGTGGGGGATCCTGA |
| Hv_Contig_1300_nlr_1 | GCAAGGGGAAGACCATCACA | TGGTGTGACATGAAGGAGGC |
| Hv_Contig_1318_nlr_1 | CAAGCAAGCCAAGACGCTTC | TCAAGAATGCACGGACGTCA |
| Hv_Contig_1410_nlr_1 | AGATACAAGGTGCCATGGCT | CTGGTTGTACTTGTACATGGAATG |
| Hv_Contig_120 | GAGAGATTTGAGAAGCACGC | GGGTTTGGTTGTACTATCAGAA |
| Hv_Contig_362 | ACCGCAGTTTGAGCTGGTAA | ATGGTATGGTGGGGCTCTCT |
| Hv_Contig_1235 | AATGCTTGCCCCTGAGTTCA | ACACGCCTGTCACATCACTT |
| Hv_Contig_1339 | TGTCCAGGAGGCGATTGAAC | AATCCTGTGGGGGATCCTGA |
| Hv_Contig_541_nlr_1 | CTGCCGAAACTTCCCCTGAT | GACTGAGCGACGGCATGTAT |
| Hv_Contig_1146_nlr_1 | GCGTTCCAACATTGCAGCTT | GCCCAAAAGTCTGCTGAGGA |
| Hv_Contig_105_nlr_1 | ACTCACGGGGCAACAACTAG | ACAGCATGCTTTCTAGGAGGT |
| Hv_Contig_253_nlr_1 | CTGAGATCAGGGATGTCGCC | CGATGGTTGACTTCCCTGCT |
| Hv_Contig_308_nlr_1 | ACAAGGAGTTCCACTGCTGG | GTCGAGACAATGACCGAGGG |
| Hv_Contig_705_nlr_1 | GATAGGGTGCCCAACTCTCG | TTGGCCCGCACGAAAAATTT |
| Hv_Contig_757_nlr_1 | GCTGATTCTCTGCGGCATTG | AATGGCTCCTGACCAAGCTC |
| Hv_Contig_937_nlr_1 | GCTGGGAAGATTGCTGCAAC | TGAGTGCGCTGAACACATCT |
| Hv_Contig_950_nlr_1 | AATTGGAAAACCTGCGCACC | GCTGAGTTGCTTTAACCCGC |
| Hv_Contig_85 | TCCGGAAGCTTCAATGCCAT | CTCATGCCACCTCCTAGCAG |
| Hv_Contig_270 | CACCTTCACCTTCGGAACCA | ACATTGTCCACTCTCCTCGG |
| Hv_Contig_1353 | ACCTTTGTATCGGTCGGCAA | TGGTGGTGCAATGTCGAAGA |
| Hv_Contig_665_nlr_1 | TAAGTGGGTGCTGAGCAAGG | ATTGATGCGGAGGCCTTGAA |
| Hv_Contig_39_nlr_2 | CAAATGAGCGACGCATGAGG | CCACGAAGGAGGCTTGACAT |
| Hv_Contig_249_nlr_2 | AGGGCAAGACATCGGGAAAG | CTACAGGGCCGTCATCATCC |
| Hv_Contig_514_nlr_1 | GTCCACATCAGCATGGGACA | AGACCTTCTGACCCTCTCCC |
| Hv_Contig_750_nlr_1 | TCCACCCTTTTCACGCACAT | CACCATAAGGAAAAGCACGGC |
| Hv_Contig_860_nlr_1 | GGGTAGGCAGGCACAAAGAT | AGCTCCCAGAACAAATCGGG |
| Hv_Contig_1012 | TTCCTGTCAAGGAAAAACCA | TTGCGGAAAAAGATTGCA |
| Hv_Contig_1162 | CACGAGTCGATGATGTGGCT | TGCTCTGCCACTCGTCTTTT |
| Hv_Contig_736 | CCCAACTTCTCCCCCACATC | TATGGGAAGCACACGCAACT |
| Hv_Contig_539 | GCGGAGAAAAGCAAGCGATT | GCCTGAGTTTCACGAGGTGA |
| Hv_Contig_38_nlr_2 | GGCGTGGATCTGATCAATAT | GTCTCAAGAACGACCCTTGC |
| Hv_Contig_55_nlr_1 | CAAGATAAGCAAGAGAGCACAC | CACTTCTTCCACATTTCTAGGC |
| Hv_Contig_99_nlr_3 | CTGTTCGCTTGCATGTGGTG | TCACCTGAGCACTCGGTTTG |
| Hv_Contig_508_nlr_1 | ATCCTGACACTGGAGTTGCG | AGGTCCGGCCTCAAATTCAG |
| Hv_Contig_1010_nlr_1 | TCTTTGCAGGTGGTGGTGGT | TTTACCTGCAACAACTCTTGTCAG |
| Hv_Contig_1236_nlr_1 | CTGCCTTGACTACCCTTGGG | GCTTCAGTACATGCGTGTCG |
| Hv_Contig_632 | ATGTAGCACCAGAGCACAGG | TGATTCGGCCAGGGTACATT |
| Hv_Contig_912 | TGCGGTACTGAGAGGGCTTA | TGGCCCCTATTTCAGCAAGT |
| Hv_Contig_414 | CTCAGGGACAACAGCTGAGG | AGCAGCAGACAAGGGGAATC |
| Hv_Contig_940 | GCACATGGTCAACACAGCAG | TGGGATGGTGCATGACCTTC |
| Hv_Contig_978 | GACCAGATTGTCCTGCAGCT | ACTGAGGCATGTTCCTGTCG |
