# Supplementary material for: Genome-wide identification of the NLR gene family in Haynaldia villosa by SMRT-RenSeq
Source: BMC Genomics. 2022 Feb 10;23:118. doi: 10.1186/s12864-022-08334-w (PMC8832786; doi:10.1186/s12864-022-08334-w)
Supplement: Supplementary file 6 — Additional file 6. [file 12864_2022_8334_MOESM6_ESM.docx]

**Table S3. The predicted chromosomal location of annotated *NLRs* in *H. villosa* and its putative orthologous NLRs in *T. aestivum, H. vulgare and A. tauschii*.**

| **NLR genes** | **Chromosomal Location** | | | |
| --- | --- | --- | --- | --- |
|  | ***T. aestivum*** | ***H. vulgare*** | ***A. tauschii*** | ***H. villosa*** |
| Hv_Contig_6_nlr_1 | chr3D | chr3H | chr3D | chr3V |
| Hv_Contig_6_nlr_2 | chr3A | chr3H | chr3D | chr3V |
| Hv_Contig_7_nlr_1 | chr5D | chr5H | chr5D | chr5V |
| Hv_Contig_8_nlr_1 | chr7B | chr7H | chr7D | chr7V |
| Hv_Contig_8_nlr_2 | chr7A | chr7H | chr7D | chr7V |
| Hv_Contig_9_nlr_1 | chr2A | chr2H | chr2D | chr2V |
| Hv_Contig_11_nlr_1 | chr3D | chr3H | chr3D | chr3V |
| Hv_Contig_13_nlr_1 | chr3B | chr3H | chr3D | chr3V |
| Hv_Contig_14_nlr_1 | chr7A | chr7H | chr7D | chr7V |
| Hv_Contig_16_nlr_1 | chr6A | chr6H | chr6D | chr6V |
| Hv_Contig_17_nlr_1 | chr5D | chr5H | chr5D | chr5V |
| Hv_Contig_19_nlr_1 | chr6A | chr6H | chr6D | chr6V |
| Hv_Contig_20_nlr_1 | chr6D | chr6H | chr6D | chr6V |
| Hv_Contig_20_nlr_2 | chr6B | chr6H | chr6D | chr6V |
| Hv_Contig_23_nlr_1 | chr7D | chr7H | chr7D | chr7V |
| Hv_Contig_25_nlr_2 | chr7D | chr7H | chr7D | chr7V |
| Hv_Contig_26_nlr_1 | chr5B | chr7H | chr5D | chr7V |
| Hv_Contig_27_nlr_1 | chr2D | chr2H | chr2D | chr2V |
| Hv_Contig_28_nlr_1 | chr7B | chr7H | chr7D | chr7V |
| Hv_Contig_29_nlr_1 | chr3A | chr3H | chr3D | chr3V |
| Hv_Contig_30_nlr_1 | chr3D | chr3H | chr3D | chr3V |
| Hv_Contig_31_nlr_2 | chr3B | chr3H | chr3D | chr3V |
| Hv_Contig_31_nlr_4 | chr3A | chr3H | chr3D | chr3V |
| Hv_Contig_32_nlr_1 | chr4B | chr4H | chr4D | chr4V |
| Hv_Contig_36_nlr_1 | chr7D | chr7H | chr7D | chr7V |
| Hv_Contig_38_nlr_1 | chr7A | chr7H | chr7D | chr7V |
| Hv_Contig_38_nlr_2 | chr7A | chr7H | chr7D | chr7V |
| Hv_Contig_39_nlr_1 | chr6B | chr6H | chr6D | chr6V |
| Hv_Contig_39_nlr_2 | chr6B | chr6H | chr6D | chr6V |
| Hv_Contig_41_nlr_1 | chr7A | chr7H | chr7D | chr7V |
| Hv_Contig_43_nlr_1 | chr6A | chr6H | chr6D | chr6V |
| Hv_Contig_44_nlr_1 | chr2D | chr2H | chr2D | chr2V |
| Hv_Contig_45_nlr_3 | chr3B | chr3H | chr3D | chr3V |
| Hv_Contig_47_nlr_1 | chr2A | chr2H | chr2D | chr2V |
| Hv_Contig_48_nlr_1 | chr7D | chr7H | chr7D | chr7V |
| Hv_Contig_48_nlr_2 | chr7D | chr7H | chr7D | chr7V |
| Hv_Contig_49_nlr_1 | chr2A | chr2H | chr2D | chr2V |
| Hv_Contig_50_nlr_1 | chr3D | chr3H | chr3D | chr3V |
| Hv_Contig_54_nlr_1 | chr3B | chr3H | chr3D | chr3V |
| Hv_Contig_55_nlr_1 | chr7D | chr7H | chr7D | chr7V |
| Hv_Contig_56_nlr_1 | chr7A | chr7H | chr7D | chr7V |
| Hv_Contig_57_nlr_1 | chr2A | chr2H | chr2D | chr2V |
| Hv_Contig_58_nlr_1 | chr7D | chr7H | chr7D | chr7V |
| Hv_Contig_59_nlr_1 | chr7D | chr7H | chr7D | chr7V |
| Hv_Contig_60_nlr_2 | chr1B | chr1H | chr1D | chr1V |
| Hv_Contig_60_nlr_3 | chr1D | chr1H | chr1D | chr1V |
| Hv_Contig_61_nlr_1 | chr6B | chr6H | chr6D | chr6V |
| Hv_Contig_64_nlr_1 | chr5B | chr5H | chr5D | chr5V |
| Hv_Contig_65_nlr_1 | chr7A | chr7H | chr7D | chr7V |
| Hv_Contig_66_nlr_1 | chr6D | chr6H | chr6D | chr6V |
| Hv_Contig_67_nlr_1 | chr6D | chr6H | chr6D | chr6V |
| Hv_Contig_69_nlr_1 | chr7D | chr7H | chr7D | chr7V |
| Hv_Contig_71_nlr_1 | chr6A | chr6H | chr6D | chr6V |
| Hv_Contig_72_nlr_1 | chr3D | chr3H | chr3D | chr3V |
| Hv_Contig_72_nlr_2 | chr3D | chr3H | chr3D | chr3V |
| Hv_Contig_73_nlr_1 | chr3B | chr3H | chr3D | chr3V |
| Hv_Contig_73_nlr_3 | chr3B | chr3H | chr3D | chr3V |
| Hv_Contig_78_nlr_1 | chr7D | chr7H | chr7D | chr7V |
| Hv_Contig_79_nlr_1 | chr3B | chr3H | chr3D | chr3V |
| Hv_Contig_80_nlr_1 | chr2B | chr2H | chr2D | chr2V |
| Hv_Contig_81_nlr_2 | chr7A | chr7H | chr7D | chr7V |
| Hv_Contig_82_nlr_2 | chr7D | chr7H | chr7D | chr7V |
| Hv_Contig_83_nlr_1 | chr1A | chr1H | chr1D | chr1V |
| Hv_Contig_86_nlr_1 | chr7A | chr7H | No Hit | chr7V |
| Hv_Contig_88_nlr_1 | chr6D | chr6H | chr6D | chr6V |
| Hv_Contig_89_nlr_1 | chr2D | chr2H | chr2D | chr2V |
| Hv_Contig_90_nlr_1 | chr4B | chrUn | chr4D | chr4V |
| Hv_Contig_92_nlr_1 | chr7B | chr7H | chr7D | chr7V |
| Hv_Contig_93_nlr_1 | chr3D | chr3H | chr3D | chr3V |
| Hv_Contig_96_nlr_1 | chr3D | chr3H | chr3D | chr3V |
| Hv_Contig_97_nlr_2 | chr5B | chr5H | chr5D | chr5V |
| Hv_Contig_99_nlr_3 | chr7D | chr7H | chr7D | chr7V |
| Hv_Contig_100_nlr_1 | chr7D | chr7H | chr7D | chr7V |
| Hv_Contig_100_nlr_2 | chr7A | chr7H | chr7D | chr7V |
| Hv_Contig_103_nlr_1 | chr1A | chr1H | chr1D | chr1V |
| Hv_Contig_105_nlr_1 | chr5D | chr5H | chr5D | chr5V |
| Hv_Contig_106_nlr_1 | chr6A | chr6H | chr6D | chr6V |
| Hv_Contig_107_nlr_1 | chr5D | chr5H | chr5D | chr5V |
| Hv_Contig_112_nlr_1 | chr3D | chr3H | chr3D | chr3V |
| Hv_Contig_114_nlr_1 | chr7B | chr7H | chr7D | chr7V |
| Hv_Contig_115_nlr_1 | chr1B | chr1H | chr3D | chr1V |
| Hv_Contig_116_nlr_1 | chr4D | chr4H | chr4D | chr4V |
| Hv_Contig_119_nlr_2 | chr1D | chr1H | chr1D | chr1V |
| Hv_Contig_122_nlr_1 | chr3D | chr3H | chr3D | chr3V |
| Hv_Contig_123_nlr_1 | chr5A | chr2H | chr2D | chr2V |
| Hv_Contig_124_nlr_1 | chr7B | chr7H | chr7D | chr7V |
| Hv_Contig_126_nlr_1 | chr4B | chr4H | chr4D | chr4V |
| Hv_Contig_127_nlr_1 | chr2B | chr2H | chr2D | chr2V |
| Hv_Contig_129_nlr_1 | chr5D | chr5H | chr5D | chr5V |
| Hv_Contig_130_nlr_1 | chr3B | chr3H | chr3D | chr3V |
| Hv_Contig_131_nlr_1 | chr3D | chr3H | chr3D | chr3V |
| Hv_Contig_132_nlr_1 | chr3B | chr3H | chr3D | chr3V |
| Hv_Contig_133_nlr_1 | chr1A | chr1H | chr1D | chr1V |
| Hv_Contig_135_nlr_1 | chr5A | chr7H | chr5D | chr5V |
| Hv_Contig_137_nlr_1 | chr7A | chr7H | chr7D | chr7V |
| Hv_Contig_138_nlr_2 | chr1A | chr1H | chr1D | chr1V |
| Hv_Contig_140_nlr_1 | chr1D | chr1H | chr1D | chr1V |
| Hv_Contig_142_nlr_1 | chr6A | chr6H | chr6D | chr6V |
| Hv_Contig_145_nlr_1 | chr1D | chr7H | chr1D | chr1V |
| Hv_Contig_148_nlr_1 | chr6A | chr6H | chr6D | chr6V |
| Hv_Contig_149_nlr_1 | chr4A | chr7H | chr7D | chr7V |
| Hv_Contig_150_nlr_1 | chr2B | chr2H | chr2D | chr2V |
| Hv_Contig_151_nlr_1 | chr7D | chr7H | chr7D | chr7V |
| Hv_Contig_152_nlr_1 | chr7A | chr7H | chr7D | chr7V |
| Hv_Contig_154_nlr_1 | chr1D | chr1H | chr1D | chr1V |
| Hv_Contig_155_nlr_2 | chr5D | chr5H | chr5D | chr5V |
| Hv_Contig_156_nlr_1 | chr3B | chr3H | chr3D | chr3V |
| Hv_Contig_157_nlr_1 | chr6B | chr6H | chr6D | chr6V |
| Hv_Contig_158_nlr_1 | chr2B | chr2H | chr2D | chr2V |
| Hv_Contig_159_nlr_1 | chr7B | chr7H | chr7D | chr7V |
| Hv_Contig_160_nlr_1 | chr7A | chr7H | chr7D | chr7V |
| Hv_Contig_161_nlr_1 | chr6B | chr6H | chr6D | chr6V |
| Hv_Contig_165_nlr_1 | chr4A | chr7H | chr7D | chr7V |
| Hv_Contig_166_nlr_1 | chr5B | chr5H | chr6D | chr5V |
| Hv_Contig_171_nlr_1 | chr5D | chr5H | chr5D | chr5V |
| Hv_Contig_172_nlr_1 | chr7B | chr7H | chr7D | chr7V |
| Hv_Contig_173_nlr_1 | chr3D | chr3H | chr3D | chr3V |
| Hv_Contig_174_nlr_1 | chr6D | chr6H | chr6D | chr6V |
| Hv_Contig_176_nlr_1 | chr5B | chr5H | No hit | chr5V |
| Hv_Contig_177_nlr_1 | chr7D | chr7H | chr7D | chr7V |
| Hv_Contig_178_nlr_1 | chr1D | chr1H | chr1D | chr1V |
| Hv_Contig_181_nlr_1 | chr1A | chr1H | chr1D | chr1V |
| Hv_Contig_182_nlr_1 | chrUn | chr6H | chr6D | chr6V |
| Hv_Contig_183_nlr_1 | chr7D | chr7H | chr7D | chr7V |
| Hv_Contig_184_nlr_1 | chr1A | chr1H | chr1D | chr1V |
| Hv_Contig_185_nlr_1 | chr7A | chr7H | chr7D | chr7V |
| Hv_Contig_186_nlr_1 | chr2D | chr2H | chr2D | chr2V |
| Hv_Contig_187_nlr_1 | chr6B | chr6H | chr6D | chr6V |
| Hv_Contig_188_nlr_1 | chr1B | chr1H | chr1D | chr1V |
| Hv_Contig_189_nlr_1 | chr2A | chr2H | chr2D | chr2V |
| Hv_Contig_191_nlr_1 | chr3D | chr3H | chr3D | chr3V |
| Hv_Contig_193_nlr_1 | chr7D | chr7H | chr7D | chr7V |
| Hv_Contig_194_nlr_1 | chr7A | chr7H | chr7D | chr7V |
| Hv_Contig_195_nlr_1 | chrUn | chr6H | chr6D | chr6V |
| Hv_Contig_196_nlr_1 | chr6A | chr6H | chr6D | chr6V |
| Hv_Contig_198_nlr_1 | chr6D | chr6H | chr6D | chr6V |
| Hv_Contig_199_nlr_1 | chr1D | chr1H | chr1D | chr1V |
| Hv_Contig_200_nlr_1 | chr7A | chr7H | chr7D | chr7V |
| Hv_Contig_202_nlr_1 | chr7D | chr7H | chr7D | chr7V |
| Hv_Contig_207_nlr_1 | chr6B | chr6H | chr6D | chr6V |
| Hv_Contig_208_nlr_1 | chr5D | chr5H | chr5D | chr5V |
| Hv_Contig_210_nlr_1 | chr2A | chr2H | chr2D | chr2V |
| Hv_Contig_211_nlr_1 | chr3D | chr3H | chr3D | chr3V |
| Hv_Contig_212_nlr_1 | chr1B | chr1H | chr1D | chr1V |
| Hv_Contig_212_nlr_2 | chr1D | chr1H | chr1D | chr1V |
| Hv_Contig_214_nlr_2 | chr3A | chr3H | chr3D | chr3V |
| Hv_Contig_216_nlr_1 | chr3D | chr3H | chr3D | chr3V |
| Hv_Contig_220_nlr_2 | chr7B | chr7H | chr7D | chr7V |
| Hv_Contig_223_nlr_1 | chr2B | chr2H | chr2D | chr2V |
| Hv_Contig_225_nlr_1 | chr2B | chr2H | chr2D | chr2V |
| Hv_Contig_227_nlr_1 | chr7A | chr7H | chr7D | chr7V |
| Hv_Contig_228_nlr_1 | chr5D | chr5H | chr5D | chr5V |
| Hv_Contig_230_nlr_1 | chr7B | chr7H | chr7D | chr7V |
| Hv_Contig_231_nlr_1 | chr6A | chr6H | chr6D | chr6V |
| Hv_Contig_232_nlr_1 | chr1B | chr1H | chr1D | chr1V |
| Hv_Contig_233_nlr_1 | chr7D | chr7H | chr7D | chr7V |
| Hv_Contig_234_nlr_1 | chr5D | chr6H | chr5D | chr5V |
| Hv_Contig_235_nlr_1 | chr3D | chr3H | chr3D | chr3V |
| Hv_Contig_238_nlr_1 | chr1D | chr1H | chr1D | chr1V |
| Hv_Contig_239_nlr_1 | chr2D | chr2H | chr2D | chr2V |
| Hv_Contig_241_nlr_1 | chr6D | chr6H | chr6D | chr6V |
| Hv_Contig_244_nlr_1 | chr1D | chr1H | chr1D | chr1V |
| Hv_Contig_245_nlr_1 | chr2B | chr2H | chr2D | chr2V |
| Hv_Contig_246_nlr_1 | chr2A | chr2H | chr2D | chr2V |
| Hv_Contig_247_nlr_1 | chr2B | chr2H | chr7D | chr2V |
| Hv_Contig_248_nlr_1 | chr3D | chr3H | chr3D | chr3V |
| Hv_Contig_248_nlr_2 | chr3D | chr3H | chr3D | chr3V |
| Hv_Contig_248_nlr_3 | chr3D | chr3H | chr3D | chr3V |
| Hv_Contig_249_nlr_2 | chr6B | chr6H | chr6D | chr6V |
| Hv_Contig_252_nlr_2 | chr1B | chr1H | chr1D | chr1V |
| Hv_Contig_253_nlr_1 | chr5A | chr5H | chr5D | chr5V |
| Hv_Contig_254_nlr_1 | chr1A | chr1H | chr1D | chr1V |
| Hv_Contig_256_nlr_1 | chr5D | chr5H | chr5D | chr5V |
| Hv_Contig_258_nlr_1 | chr7D | chr7H | chr7D | chr7V |
| Hv_Contig_259_nlr_2 | chr3B | chr3H | chr3D | chr3V |
| Hv_Contig_260_nlr_1 | chr7B | chr7H | chr7D | chr7V |
| Hv_Contig_261_nlr_1 | chr6B | chr6H | chr6D | chr6V |
| Hv_Contig_263_nlr_1 | chr3B | chr3H | chr3D | chr3V |
| Hv_Contig_264_nlr_1 | chr7B | chr5H | chr7D | chr7V |
| Hv_Contig_266_nlr_1 | chr3B | chr3H | chr3D | chr3V |
| Hv_Contig_267_nlr_1 | chr3A | chr3H | chr3D | chr3V |
| Hv_Contig_268_nlr_1 | chr1B | chr1H | chr1D | chr1V |
| Hv_Contig_277_nlr_1 | chr7B | chr7H | chr7D | chr7V |
| Hv_Contig_280_nlr_1 | chr1B | chr1H | chr1D | chr1V |
| Hv_Contig_281_nlr_1 | chr7B | chr7H | chr7D | chr7V |
| Hv_Contig_282_nlr_1 | chr1D | chr1H | chr1D | chr1V |
| Hv_Contig_283_nlr_1 | chr7A | chr7H | chr7D | chr7V |
| Hv_Contig_284_nlr_1 | chr1B | chr1H | chr1D | chr1V |
| Hv_Contig_285_nlr_1 | chr7A | chr7H | chr7D | chr7V |
| Hv_Contig_286_nlr_1 | chr3D | chr3H | chr3D | chr3V |
| Hv_Contig_289_nlr_1 | chr1D | chr1H | chr1D | chr1V |
| Hv_Contig_291_nlr_1 | chr7A | chr7H | chr7D | chr7V |
| Hv_Contig_292_nlr_1 | chr2A | chr2H | chr2D | chr2V |
| Hv_Contig_293_nlr_1 | chr1A | chr1H | chr1D | chr1V |
| Hv_Contig_294_nlr_1 | chr5A | chr5H | chr5D | chr5V |
| Hv_Contig_295_nlr_1 | chr5D | chr5H | chr5D | chr5V |
| Hv_Contig_296_nlr_1 | chr5A | chr5H | chr5D | chr5V |
| Hv_Contig_297_nlr_1 | chr5D | chr5H | chr5D | chr5V |
| Hv_Contig_298_nlr_1 | chr1D | chr1H | chr1D | chr1V |
| Hv_Contig_299_nlr_1 | chr7D | chr7H | chr7D | chr7V |
| Hv_Contig_302_nlr_1 | chr6B | chr6H | chr6D | chr6V |
| Hv_Contig_303_nlr_1 | chr3B | chr3H | chr3D | chr3V |
| Hv_Contig_306_nlr_1 | chr7D | chr7H | chr7D | chr7V |
| Hv_Contig_307_nlr_1 | chr7D | chr7H | chr7D | chr7V |
| Hv_Contig_308_nlr_1 | chr5B | chr5H | chr7D | chr5V |
| Hv_Contig_309_nlr_1 | chr1B | chr1H | chr1D | chr1V |
| Hv_Contig_314_nlr_1 | chr3B | chr3H | chr3D | chr3V |
| Hv_Contig_315_nlr_1 | chr7A | chr2H | chr7D | chr7V |
| Hv_Contig_316_nlr_1 | chr4D | chr4H | chr4D | chr4V |
| Hv_Contig_317_nlr_1 | chr6A | chr6H | chr6D | chr6V |
| Hv_Contig_320_nlr_1 | chr3B | chr3H | chr3D | chr3V |
| Hv_Contig_320_nlr_2 | chr3B | chr3H | chr3D | chr3V |
| Hv_Contig_321_nlr_1 | chr2D | chr2H | chr2D | chr2V |
| Hv_Contig_322_nlr_2 | chr4B | chr4H | chr4D | chr4V |
| Hv_Contig_324_nlr_1 | chr1D | chr1H | chr1D | chr1V |
| Hv_Contig_324_nlr_2 | chr1D | chr1H | chr1D | chr1V |
| Hv_Contig_326_nlr_1 | chr3D | chr3H | chr3D | chr3V |
| Hv_Contig_327_nlr_1 | chr7B | chr7H | chr7D | chr7V |
| Hv_Contig_328_nlr_1 | chr3D | chr3H | chr3D | chr3V |
| Hv_Contig_329_nlr_1 | chr6D | chr6H | chr6D | chr6V |
| Hv_Contig_330_nlr_2 | chr3A | chr3H | chr3D | chr3V |
| Hv_Contig_331_nlr_1 | chr3B | chr3H | chr3D | chr3V |
| Hv_Contig_335_nlr_1 | chr5D | chr6H | chr5D | chr5V |
| Hv_Contig_338_nlr_2 | chr1D | chr7H | chr1D | chr1V |
| Hv_Contig_338_nlr_3 | chr1D | chr7H | chr1D | chr1V |
| Hv_Contig_340_nlr_1 | chr3B | chr3H | chr3D | chr3V |
| Hv_Contig_343_nlr_1 | chr1B | chr1H | chr1D | chr1V |
| Hv_Contig_345_nlr_1 | chr1B | chr1H | chr1D | chr1V |
| Hv_Contig_347_nlr_1 | chr6D | chr6H | chr6D | chr6V |
| Hv_Contig_349_nlr_1 | chr1A | chr1H | chr1D | chr1V |
| Hv_Contig_350_nlr_1 | chr2D | chr2H | chr2D | chr2V |
| Hv_Contig_351_nlr_1 | chr4B | chr4H | chr4D | chr4V |
| Hv_Contig_351_nlr_2 | chr4D | chr4H | chr4D | chr4V |
| Hv_Contig_352_nlr_1 | chr2D | chr2H | chr2D | chr2V |
| Hv_Contig_353_nlr_1 | chr4A | chr7H | chr7D | chr7V |
| Hv_Contig_354_nlr_1 | chr7A | chr7H | chr7D | chr7V |
| Hv_Contig_355_nlr_1 | chr7D | chr7H | chr7D | chr7V |
| Hv_Contig_356_nlr_1 | chr1B | chr1H | chr1D | chr1V |
| Hv_Contig_357_nlr_1 | chr7A | chr7H | chr7D | chr7V |
| Hv_Contig_359_nlr_1 | chr3B | chr3H | chr3D | chr3V |
| Hv_Contig_363_nlr_1 | chr7A | chr7H | chr7D | chr7V |
| Hv_Contig_364_nlr_1 | chr3B | chr3H | chr3D | chr3V |
| Hv_Contig_366_nlr_1 | chr3B | chr3H | chr3D | chr3V |
| Hv_Contig_367_nlr_1 | chr7D | chr7H | chr7D | chr7V |
| Hv_Contig_368_nlr_1 | chr2A | chr2H | No hit | chr2V |
| Hv_Contig_369_nlr_1 | chr7A | chr7H | chr7D | chr7V |
| Hv_Contig_370_nlr_1 | chr3B | chr3H | chr3D | chr3V |
| Hv_Contig_371_nlr_1 | chr7B | chr7H | chr7D | chr7V |
| Hv_Contig_374_nlr_1 | chr3B | chr3H | chr3D | chr3V |
| Hv_Contig_379_nlr_1 | chr4D | chr4H | chr4D | chr4V |
| Hv_Contig_382_nlr_1 | chr7A | chr7H | chr7D | chr7V |
| Hv_Contig_383_nlr_1 | chr3B | chr3H | chr3D | chr3V |
| Hv_Contig_384_nlr_1 | chr3D | chr3H | chr3D | chr3V |
| Hv_Contig_384_nlr_2 | chr3B | chr3H | chr3D | chr3V |
| Hv_Contig_387_nlr_1 | chr6B | chr6H | chr6D | chr6V |
| Hv_Contig_388_nlr_1 | chr3D | chr3H | chr3D | chr3V |
| Hv_Contig_390_nlr_1 | chr6D | chr6H | chr6D | chr6V |
| Hv_Contig_391_nlr_2 | chr3B | chr3H | chr3D | chr3V |
| Hv_Contig_393_nlr_1 | chr7A | chr7H | chr7D | chr7V |
| Hv_Contig_394_nlr_2 | chr3D | chr3H | chr3D | chr3V |
| Hv_Contig_395_nlr_1 | chr6B | chr6H | chr6D | chr6V |
| Hv_Contig_396_nlr_1 | chr1B | chr1H | chr1D | chr1V |
| Hv_Contig_397_nlr_1 | chr7D | chr7H | chr7D | chr7V |
| Hv_Contig_398_nlr_1 | chr6A | chr6H | chr6D | chr6V |
| Hv_Contig_399_nlr_1 | chr1D | chr1H | chr1D | chr1V |
| Hv_Contig_403_nlr_1 | chr6A | chr6H | chr6D | chr6V |
| Hv_Contig_405_nlr_1 | chr3D | chr3H | chr3D | chr3V |
| Hv_Contig_406_nlr_2 | chr3B | chr3H | chr3D | chr3V |
| Hv_Contig_408_nlr_1 | chr2B | chr2H | chr2D | chr2V |
| Hv_Contig_409_nlr_1 | chr6A | chr6H | chr6D | chr6V |
| Hv_Contig_410_nlr_1 | chr3D | chr3H | chr3D | chr3V |
| Hv_Contig_412_nlr_1 | chr2B | chr2H | chr2D | chr2V |
| Hv_Contig_415_nlr_1 | chr3D | chr3H | chr3D | chr3V |
| Hv_Contig_417_nlr_1 | chr7A | chr7H | chr7D | chr7V |
| Hv_Contig_419_nlr_1 | chr6D | chr6H | chr6D | chr6V |
| Hv_Contig_422_nlr_1 | chr1D | chr1H | chr1D | chr1V |
| Hv_Contig_424_nlr_1 | chr6D | chr6H | chr6D | chr6V |
| Hv_Contig_425_nlr_1 | chr3D | chr3H | chr3D | chr3V |
| Hv_Contig_429_nlr_1 | chr7B | chr7H | chr7D | chr7V |
| Hv_Contig_430_nlr_1 | chr2B | chr5H | chr2D | chr2V |
| Hv_Contig_431_nlr_1 | chr3B | chr3H | chr3D | chr3V |
| Hv_Contig_433_nlr_1 | chr5B | chr5H | chr5D | chr5V |
| Hv_Contig_435_nlr_1 | chr1A | chr1H | chr1D | chr1V |
| Hv_Contig_436_nlr_1 | chr1B | chr1H | chr1D | chr1V |
| Hv_Contig_437_nlr_1 | chr7A | chr7H | chr7D | chr7V |
| Hv_Contig_438_nlr_1 | chr3A | chr3H | chr3D | chr3V |
| Hv_Contig_441_nlr_1 | chr6D | chr6H | chr6D | chr6V |
| Hv_Contig_442_nlr_1 | chr7A | chr7H | chr7D | chr7V |
| Hv_Contig_443_nlr_1 | chr1B | chr1H | chr1D | chr1V |
| Hv_Contig_445_nlr_1 | chr2B | chr2H | chr2D | chr2V |
| Hv_Contig_446_nlr_1 | chr6B | chr6H | chr6D | chr6V |
| Hv_Contig_447_nlr_1 | chr4B | chr4H | chr4D | chr4V |
| Hv_Contig_448_nlr_1 | chr2A | chr2H | chr2D | chr2V |
| Hv_Contig_449_nlr_1 | chr1D | chr1H | chr1D | chr1V |
| Hv_Contig_450_nlr_1 | chr7A | chr7H | chr7D | chr7V |
| Hv_Contig_452_nlr_1 | chr1D | chr1H | chr1D | chr1V |
| Hv_Contig_455_nlr_1 | chr7D | chr7H | chr7D | chr7V |
| Hv_Contig_458_nlr_1 | chr7D | chr7H | chr7D | chr7V |
| Hv_Contig_460_nlr_1 | chr3D | chr3H | chr3D | chr3V |
| Hv_Contig_461_nlr_1 | chr2D | chr2H | chr2D | chr2V |
| Hv_Contig_462_nlr_1 | chr3A | chr3H | chr3D | chr3V |
| Hv_Contig_463_nlr_1 | chr6A | chrUn | chr3D | chr6V |
| Hv_Contig_465_nlr_1 | chr3B | chr3H | chr3D | chr3V |
| Hv_Contig_467_nlr_1 | chr1A | chr1H | chr1D | chr1V |
| Hv_Contig_468_nlr_1 | chr7D | chr7H | chr7D | chr7V |
| Hv_Contig_469_nlr_1 | chr7D | chr7H | chr7D | chr7V |
| Hv_Contig_471_nlr_1 | chr2D | chr2H | chr2D | chr2V |
| Hv_Contig_473_nlr_1 | chr3B | chr3H | chr3D | chr3V |
| Hv_Contig_476_nlr_1 | chr7B | chr7H | chr7D | chr7V |
| Hv_Contig_477_nlr_1 | chr7B | chr7H | chr7D | chr7V |
| Hv_Contig_479_nlr_1 | chr3B | chr3H | chr3D | chr3V |
| Hv_Contig_481_nlr_1 | chr1D | chr6H | chr1D | chr1V |
| Hv_Contig_486_nlr_1 | chrUn | chr6H | chr6D | chr6V |
| Hv_Contig_488_nlr_1 | chr3A | chr3H | chr3D | chr3V |
| Hv_Contig_489_nlr_1 | chr7A | chr7H | chr7D | chr7V |
| Hv_Contig_493_nlr_2 | chr3A | chr3H | chr3D | chr3V |
| Hv_Contig_496_nlr_1 | chr1B | chr1H | chr1D | chr1V |
| Hv_Contig_499_nlr_1 | chr1D | chr1H | chr1D | chr1V |
| Hv_Contig_500_nlr_1 | chr1D | chr1H | chr1D | chr1V |
| Hv_Contig_504_nlr_1 | chr6B | chr6H | chr6D | chr6V |
| Hv_Contig_506_nlr_1 | chr6B | chr6H | chr6D | chr6V |
| Hv_Contig_510_nlr_1 | chr6D | chr6H | chr6D | chr6V |
| Hv_Contig_512_nlr_1 | chr2A | chr2H | chr2D | chr2V |
| Hv_Contig_514_nlr_1 | chr6D | chr6H | chr6D | chr6V |
| Hv_Contig_516_nlr_1 | chr6D | chr6H | chr6D | chr6V |
| Hv_Contig_517_nlr_1 | chr2D | chr2H | chr2D | chr2V |
| Hv_Contig_521_nlr_1 | chr4A | chr4H | chr4D | chr4V |
| Hv_Contig_522_nlr_1 | chr1D | chr1H | chr1D | chr1V |
| Hv_Contig_523_nlr_2 | chr7D | chr5H | chr7D | chr7V |
| Hv_Contig_524_nlr_1 | chr3B | chr3H | chr3D | chr3V |
| Hv_Contig_525_nlr_1 | chr1D | chr1H | chr1D | chr1V |
| Hv_Contig_527_nlr_1 | chr2A | chr2H | chr2D | chr2V |
| Hv_Contig_528_nlr_1 | chr6A | chr6H | chr6D | chr6V |
| Hv_Contig_529_nlr_1 | chr2A | chr2H | chr2D | chr2V |
| Hv_Contig_532_nlr_1 | chr7A | chr7H | chr7D | chr7V |
| Hv_Contig_533_nlr_1 | chr2B | chr2H | chr2D | chr2V |
| Hv_Contig_534_nlr_1 | chr3B | chr3H | chr3D | chr3V |
| Hv_Contig_535_nlr_1 | chr6A | chr6H | chr6D | chr6V |
| Hv_Contig_537_nlr_1 | chr7D | chr7H | chr7D | chr7V |
| Hv_Contig_538_nlr_1 | chr7B | chr7H | chr7D | chr7V |
| Hv_Contig_541_nlr_1 | chr5D | chr5H | chr5D | chr5V |
| Hv_Contig_542_nlr_1 | chr2D | chr2H | chr2D | chr2V |
| Hv_Contig_544_nlr_1 | chr2D | chr2H | chr2D | chr2V |
| Hv_Contig_544_nlr_2 | chr2D | chr2H | chr2D | chr2V |
| Hv_Contig_545_nlr_1 | chr1B | chr1H | chr1D | chr1V |
| Hv_Contig_551_nlr_1 | chr1B | chr1H | chr1D | chr1V |
| Hv_Contig_554_nlr_1 | chr1D | chr1H | chr1D | chr1V |
| Hv_Contig_555_nlr_1 | chr7A | chr7H | chr7D | chr7V |
| Hv_Contig_556_nlr_1 | chr7D | chr7H | chr7D | chr7V |
| Hv_Contig_557_nlr_1 | chr7A | chr7H | chr7D | chr7V |
| Hv_Contig_558_nlr_1 | chr4A | chr7H | chr7D | chr7V |
| Hv_Contig_559_nlr_1 | chr1D | chr1H | chr1D | chr1V |
| Hv_Contig_565_nlr_1 | chr6D | chr6H | chr6D | chr6V |
| Hv_Contig_566_nlr_1 | chr6B | chr6H | chr6D | chr6V |
| Hv_Contig_567_nlr_1 | chr3B | chr3H | chr3D | chr3V |
| Hv_Contig_568_nlr_1 | chr6B | chr6H | chr6D | chr6V |
| Hv_Contig_569_nlr_1 | chr2A | chr2H | No hit | chr2V |
| Hv_Contig_570_nlr_1 | chr3B | chr3H | No hit | chr3V |
| Hv_Contig_571_nlr_1 | chr7A | chr7H | chr7D | chr7V |
| Hv_Contig_572_nlr_1 | chr5D | chr6H | chr5D | chr5V |
| Hv_Contig_576_nlr_1 | chr7D | chr7H | chr7D | chr7V |
| Hv_Contig_578_nlr_1 | chr3B | chr3H | chr3D | chr3V |
| Hv_Contig_579_nlr_1 | chr3D | chr3H | chr3D | chr3V |
| Hv_Contig_580_nlr_1 | chr3D | chr3H | chr3D | chr3V |
| Hv_Contig_581_nlr_1 | chr7B | chr7H | chr7D | chr7V |
| Hv_Contig_582_nlr_1 | chr5D | chr5H | chr5D | chr5V |
| Hv_Contig_584_nlr_1 | chr3B | chr3H | chr3D | chr3V |
| Hv_Contig_589_nlr_1 | chr5A | chr5H | chr5D | chr5V |
| Hv_Contig_590_nlr_1 | chr3B | chr3H | chr3D | chr3V |
| Hv_Contig_591_nlr_1 | chr2D | chr2H | chr2D | chr2V |
| Hv_Contig_592_nlr_1 | chr3B | chr3H | chr3D | chr3V |
| Hv_Contig_593_nlr_1 | chr2A | chr2H | chr2D | chr2V |
| Hv_Contig_594_nlr_1 | chr3B | chr3H | chr3D | chr3V |
| Hv_Contig_595_nlr_1 | chr3D | chr3H | chr3D | chr3V |
| Hv_Contig_600_nlr_1 | chr3D | chr3H | chr3D | chr3V |
| Hv_Contig_602_nlr_1 | chr1B | chr1H | chr1D | chr1V |
| Hv_Contig_605_nlr_1 | chr1B | chr1H | chr3D | chr1V |
| Hv_Contig_606_nlr_1 | chr3B | chr3H | chr3D | chr3V |
| Hv_Contig_610_nlr_1 | chr7B | chr7H | chr7D | chr7V |
| Hv_Contig_616_nlr_1 | chr1D | chr1H | chr1D | chr1V |
| Hv_Contig_617_nlr_1 | chr7B | chr7H | chr7D | chr7V |
| Hv_Contig_620_nlr_1 | chr7A | chr7H | chr7D | chr7V |
| Hv_Contig_621_nlr_1 | chr2D | No hit | chr6D | chr2V |
| Hv_Contig_622_nlr_1 | chr7B | chr7H | chr7D | chr7V |
| Hv_Contig_623_nlr_1 | chr3A | chr3H | chr3D | chr3V |
| Hv_Contig_625_nlr_1 | chr1A | chr1H | chr1D | chr1V |
| Hv_Contig_626_nlr_1 | chr5B | chr3H | chr5D | chr5V |
| Hv_Contig_629_nlr_1 | chr6D | chr6H | chr6D | chr6V |
| Hv_Contig_631_nlr_1 | chr7A | chr7H | chr7D | chr7V |
| Hv_Contig_633_nlr_1 | chr7A | chr7H | chr7D | chr7V |
| Hv_Contig_638_nlr_1 | chr7A | chr7H | chr7D | chr7V |
| Hv_Contig_639_nlr_1 | chr5D | chr7H | chr5D | chr5V |
| Hv_Contig_648_nlr_1 | chr7D | chr7H | chr7D | chr7V |
| Hv_Contig_649_nlr_1 | chr1A | chr1H | chr1D | chr1V |
| Hv_Contig_650_nlr_1 | chr1D | chr1H | chr1D | chr1V |
| Hv_Contig_651_nlr_2 | chr3A | chr3H | chr3D | chr3V |
| Hv_Contig_653_nlr_1 | chr1B | chr1H | chr3D | chr1V |
| Hv_Contig_655_nlr_1 | chr3B | chr3H | chr3D | chr3V |
| Hv_Contig_655_nlr_2 | chr3A | chr3H | chr3D | chr3V |
| Hv_Contig_656_nlr_1 | chr6B | chr6H | No hit | chr6V |
| Hv_Contig_656_nlr_2 | chr6B | chr6H | No hit | chr6V |
| Hv_Contig_657_nlr_1 | chr3A | chr3H | chr3D | chr3V |
| Hv_Contig_657_nlr_2 | chr3A | chr3H | chr3D | chr3V |
| Hv_Contig_658_nlr_1 | chr7D | chr7H | chr7D | chr7V |
| Hv_Contig_663_nlr_1 | chr1A | chr1H | chr1D | chr1V |
| Hv_Contig_665_nlr_1 | chr6B | chr6H | chr6D | chr6V |
| Hv_Contig_666_nlr_2 | chr3B | chr3H | chr3D | chr3V |
| Hv_Contig_668_nlr_1 | chr1D | chr1H | chr1D | chr1V |
| Hv_Contig_670_nlr_1 | chr4B | chr4H | chr4D | chr4V |
| Hv_Contig_671_nlr_2 | chr7A | chr7H | chr7D | chr7V |
| Hv_Contig_672_nlr_2 | chr1B | chr1H | chr1D | chr1V |
| Hv_Contig_673_nlr_1 | chr1D | chr1H | chr1D | chr1V |
| Hv_Contig_674_nlr_1 | chr5D | chr5H | chr5D | chr5V |
| Hv_Contig_675_nlr_1 | chr1A | chr1H | chr1D | chr1V |
| Hv_Contig_676_nlr_2 | chr3B | chr3H | chr3D | chr3V |
| Hv_Contig_677_nlr_1 | chr3D | chr3H | chr3D | chr3V |
| Hv_Contig_678_nlr_2 | chr1D | chr1H | chr1D | chr1V |
| Hv_Contig_679_nlr_1 | chr3D | chr3H | chr3D | chr3V |
| Hv_Contig_681_nlr_1 | chr2B | chr2H | chr2D | chr2V |
| Hv_Contig_684_nlr_1 | chr6B | chr6H | chr6D | chr6V |
| Hv_Contig_687_nlr_1 | chr7A | chr7H | chr7D | chr7V |
| Hv_Contig_690_nlr_1 | chr6B | chr6H | chr6D | chr6V |
| Hv_Contig_693_nlr_1 | chr1B | chr1H | chr1D | chr1V |
| Hv_Contig_695_nlr_1 | chr1B | chr1H | chr1D | chr1V |
| Hv_Contig_696_nlr_1 | chr5B | chr5H | No hit | chr5V |
| Hv_Contig_699_nlr_1 | chr3B | chr3H | chr3D | chr3V |
| Hv_Contig_700_nlr_1 | chr7D | chr7H | chr7D | chr7V |
| Hv_Contig_705_nlr_1 | chr5D | chr5H | chr5D | chr5V |
| Hv_Contig_708_nlr_1 | chr2A | chr2H | chr2D | chr2V |
| Hv_Contig_712_nlr_1 | chr7A | chr7H | chr7D | chr7V |
| Hv_Contig_715_nlr_1 | chr6D | No hit | chr6D | chr6V |
| Hv_Contig_716_nlr_1 | chr3A | chr3H | chr3D | chr3V |
| Hv_Contig_717_nlr_1 | chr5B | chr7H | chr5D | chr5V |
| Hv_Contig_718_nlr_1 | chr3B | chr3H | chr3D | chr3V |
| Hv_Contig_719_nlr_1 | chr1A | chr1H | chr1D | chr1V |
| Hv_Contig_720_nlr_1 | chr3B | chr3H | chr3D | chr3V |
| Hv_Contig_721_nlr_1 | chr1D | chr1H | chr1D | chr1V |
| Hv_Contig_723_nlr_1 | chr3B | chr3H | chr3D | chr3V |
| Hv_Contig_727_nlr_1 | chr1D | chr1H | chr1D | chr1V |
| Hv_Contig_729_nlr_1 | chr7D | chr5H | chr7D | chr7V |
| Hv_Contig_730_nlr_1 | chr7D | chr7H | chr7D | chr7V |
| Hv_Contig_731_nlr_1 | chr6B | chr6H | chr6D | chr6V |
| Hv_Contig_733_nlr_1 | chr2D | chr2H | chr2D | chr2V |
| Hv_Contig_734_nlr_1 | chr5A | chr5H | chr5D | chr5V |
| Hv_Contig_735_nlr_1 | chr2B | chr2H | chr2D | chr2V |
| Hv_Contig_738_nlr_1 | chr3D | chr3H | chr3D | chr3V |
| Hv_Contig_739_nlr_1 | chr3A | chr3H | chr3D | chr3V |
| Hv_Contig_744_nlr_1 | chr7D | chr7H | chr7D | chr7V |
| Hv_Contig_746_nlr_1 | chr7B | chr7H | chr7D | chr7V |
| Hv_Contig_750_nlr_1 | chr6B | chr6H | chr5D | chr6V |
| Hv_Contig_751_nlr_1 | chr1D | chr1H | chr1D | chr1V |
| Hv_Contig_752_nlr_1 | chr7D | chr7H | chr7D | chr7V |
| Hv_Contig_754_nlr_1 | chr6A | chr6H | chr6D | chr6V |
| Hv_Contig_757_nlr_1 | chr5D | chr5H | chr5D | chr5V |
| Hv_Contig_758_nlr_1 | chr3A | chr3H | chr3D | chr3V |
| Hv_Contig_759_nlr_1 | chr7A | chr7H | chr7D | chr7V |
| Hv_Contig_761_nlr_1 | chr7B | chr7H | chr7D | chr7V |
| Hv_Contig_762_nlr_1 | chr3A | chr3H | chr3D | chr3V |
| Hv_Contig_763_nlr_1 | chr7A | chr7H | chr7D | chr7V |
| Hv_Contig_764_nlr_1 | chr2D | chr2H | chr2D | chr2V |
| Hv_Contig_766_nlr_1 | chr6B | chr6H | chr6D | chr6V |
| Hv_Contig_767_nlr_1 | chr3B | chr3H | chr3D | chr3V |
| Hv_Contig_769_nlr_1 | chr5B | chr5H | chr5D | chr5V |
| Hv_Contig_771_nlr_1 | chr2D | chr2H | chr2D | chr2V |
| Hv_Contig_773_nlr_1 | chr4A | chr7H | chr7D | chr7V |
| Hv_Contig_774_nlr_1 | chr1A | chr1H | chr1D | chr1V |
| Hv_Contig_776_nlr_1 | chr3B | chr3H | chr3D | chr3V |
| Hv_Contig_777_nlr_1 | chr5D | chr5H | chr5D | chr5V |
| Hv_Contig_778_nlr_1 | chr2D | chr2H | chr2D | chr2V |
| Hv_Contig_779_nlr_1 | chr2A | chr6H | chr2D | chr2V |
| Hv_Contig_780_nlr_1 | chr7B | chr7H | chr7D | chr7V |
| Hv_Contig_781_nlr_1 | chr3D | chr3H | chr3D | chr3V |
| Hv_Contig_782_nlr_1 | chr3B | chr3H | chr3D | chr3V |
| Hv_Contig_785_nlr_1 | chr1D | chr1H | chr1D | chr1V |
| Hv_Contig_788_nlr_1 | chr3A | chr3H | chr3D | chr3V |
| Hv_Contig_790_nlr_1 | chr1A | chr1H | chr1D | chr1V |
| Hv_Contig_794_nlr_1 | chr1D | chr1H | chr1D | chr1V |
| Hv_Contig_795_nlr_1 | chr1D | chr1H | chr1D | chr1V |
| Hv_Contig_798_nlr_1 | chr7D | chr7H | chr7D | chr7V |
| Hv_Contig_799_nlr_1 | chr3D | chr3H | chr3D | chr3V |
| Hv_Contig_800_nlr_1 | chr7A | chr7H | chr7D | chr7V |
| Hv_Contig_801_nlr_1 | chr2B | chr2H | chr2D | chr2V |
| Hv_Contig_802_nlr_1 | chr6B | chr6H | chr6D | chr6V |
| Hv_Contig_805_nlr_1 | chr7D | chr7H | chr7D | chr7V |
| Hv_Contig_810_nlr_1 | chr5D | chr6H | chr5D | chr5V |
| Hv_Contig_813_nlr_1 | chr6D | chr6H | chr6D | chr6V |
| Hv_Contig_814_nlr_1 | chr7D | chr7H | chr7D | chr7V |
| Hv_Contig_817_nlr_1 | chr2D | chr2H | chr2D | chr2V |
| Hv_Contig_819_nlr_1 | chr3A | chr3H | chr3D | chr3V |
| Hv_Contig_820_nlr_1 | chr7B | chr7H | chr7D | chr7V |
| Hv_Contig_821_nlr_1 | chr3D | chr3H | chr3D | chr3V |
| Hv_Contig_823_nlr_1 | chr1B | chr1H | chr1D | chr1V |
| Hv_Contig_824_nlr_1 | chr7D | chr7H | chr7D | chr7V |
| Hv_Contig_826_nlr_1 | chr7D | chr7H | chr7D | chr7V |
| Hv_Contig_827_nlr_1 | chr1D | chr1H | chr1D | chr1V |
| Hv_Contig_829_nlr_1 | chr3A | chr3H | chr3D | chr3V |
| Hv_Contig_831_nlr_1 | chr1D | chr1H | chr1D | chr1V |
| Hv_Contig_837_nlr_1 | chr1D | chr1H | chr1D | chr1V |
| Hv_Contig_841_nlr_1 | chr5B | chr5H | chr5B | chr5V |
| Hv_Contig_844_nlr_2 | chr3B | chr3H | chr3D | chr3V |
| Hv_Contig_845_nlr_1 | chr1D | chr1H | chr1D | chr1V |
| Hv_Contig_845_nlr_2 | chr1D | chr1H | chr1D | chr1V |
| Hv_Contig_846_nlr_1 | chr3D | chr3H | chr3D | chr3V |
| Hv_Contig_848_nlr_2 | chr6B | chr6H | chr6D | chr6V |
| Hv_Contig_849_nlr_2 | chr6B | chr6H | chr6D | chr6V |
| Hv_Contig_850_nlr_1 | chr7B | chr7H | chr7D | chr7V |
| Hv_Contig_851_nlr_1 | chr1A | chr1H | chr1D | chr1V |
| Hv_Contig_854_nlr_1 | chr3A | chr3H | chr3D | chr3V |
| Hv_Contig_857_nlr_1 | chr1A | chr1H | chr1D | chr1V |
| Hv_Contig_860_nlr_1 | chr6A | chr6H | chr6D | chr6V |
| Hv_Contig_865_nlr_2 | chr1B | chr1H | chr1D | chr1V |
| Hv_Contig_866_nlr_1 | chr3B | chr3H | chr3D | chr3V |
| Hv_Contig_867_nlr_1 | chr3B | chr3H | chr3D | chr3V |
| Hv_Contig_868_nlr_1 | chr7D | chr7H | chr7D | chr7V |
| Hv_Contig_869_nlr_2 | chr5D | chr5H | chr5D | chr5V |
| Hv_Contig_870_nlr_1 | chr2D | chr1H | chr2D | chr2V |
| Hv_Contig_871_nlr_2 | chr7D | chr5H | chr7D | chr7V |
| Hv_Contig_872_nlr_1 | chr2D | chr2H | chr2D | chr2V |
| Hv_Contig_873_nlr_1 | chr1B | chr1H | chr1D | chr1V |
| Hv_Contig_879_nlr_1 | chr6A | chr5H | chr6D | chr6V |
| Hv_Contig_881_nlr_1 | chr1B | chr1H | chr1D | chr1V |
| Hv_Contig_884_nlr_1 | chr7A | chr2H | chr7D | chr7V |
| Hv_Contig_887_nlr_1 | chr6A | chr6H | chr6D | chr6V |
| Hv_Contig_888_nlr_1 | chr1A | chr1H | chr1D | chr1V |
| Hv_Contig_889_nlr_1 | chr3D | chr3H | chr3D | chr3V |
| Hv_Contig_893_nlr_1 | chr2D | chr2H | chr2D | chr2V |
| Hv_Contig_894_nlr_1 | chr3D | chr3H | chr3D | chr3V |
| Hv_Contig_896_nlr_1 | chr7A | chr7H | chr7D | chr7V |
| Hv_Contig_898_nlr_1 | chr1B | chr1H | chr1D | chr1V |
| Hv_Contig_900_nlr_1 | chr7B | chr7H | chr7D | chr7V |
| Hv_Contig_906_nlr_1 | chr6D | chr6H | chr6D | chr6V |
| Hv_Contig_908_nlr_1 | chr7D | chr7H | chr7D | chr7V |
| Hv_Contig_909_nlr_1 | chr2B | chr2H | chr2D | chr2V |
| Hv_Contig_911_nlr_1 | chr2D | chr2H | chr2D | chr2V |
| Hv_Contig_913_nlr_2 | chr7D | chr7H | chr7D | chr7V |
| Hv_Contig_919_nlr_1 | chr6D | chr6H | chr6D | chr6V |
| Hv_Contig_921_nlr_1 | chr3B | chr3H | chr3D | chr3V |
| Hv_Contig_922_nlr_1 | chr3B | chr3H | chr3D | chr3V |
| Hv_Contig_924_nlr_1 | chr1D | chr1H | chr1D | chr1V |
| Hv_Contig_925_nlr_1 | chr7A | chr7H | chr7D | chr7V |
| Hv_Contig_926_nlr_1 | chr6B | chr6H | chr6D | chr6V |
| Hv_Contig_930_nlr_1 | chr7A | chr7H | chr7D | chr7V |
| Hv_Contig_931_nlr_1 | chr2B | chr2H | chr2D | chr2V |
| Hv_Contig_932_nlr_1 | chr2D | chr2H | chr2D | chr2V |
| Hv_Contig_935_nlr_1 | chr7B | chr7H | chr7D | chr7V |
| Hv_Contig_936_nlr_1 | chr7B | chr7H | chr7D | chr7V |
| Hv_Contig_937_nlr_1 | chr5B | chrUn | chr5D | chr5V |
| Hv_Contig_939_nlr_1 | chr6B | chr6H | chr6D | chr6V |
| Hv_Contig_942_nlr_1 | chr7A | chr7H | chr7D | chr7V |
| Hv_Contig_943_nlr_1 | chr2D | chr2H | chr2D | chr2V |
| Hv_Contig_947_nlr_1 | chr1D | chr1H | chr1D | chr1V |
| Hv_Contig_948_nlr_1 | chr7D | chr3H | chr2D | chr7V |
| Hv_Contig_950_nlr_1 | chr5B | chr5H | chr5D | chr5V |
| Hv_Contig_954_nlr_1 | chr3B | chr3H | chr3D | chr3V |
| Hv_Contig_955_nlr_1 | chr3D | chr5H | chr3D | chr3V |
| Hv_Contig_957_nlr_1 | chr2B | chr2H | chr2D | chr2V |
| Hv_Contig_958_nlr_1 | chr7B | chr7H | chr7D | chr7V |
| Hv_Contig_962_nlr_1 | chr7B | chr7H | chr7D | chr7V |
| Hv_Contig_963_nlr_1 | chr7A | chr7H | chr7D | chr7V |
| Hv_Contig_967_nlr_1 | chr7D | chr7H | chr7D | chr7V |
| Hv_Contig_968_nlr_1 | chr3D | chr3H | chr3D | chr3V |
| Hv_Contig_971_nlr_1 | chr2B | chr2H | chr2D | chr2V |
| Hv_Contig_972_nlr_1 | chr2A | chr2H | chr2D | chr2V |
| Hv_Contig_979_nlr_1 | chr7D | chr7H | chr7D | chr7V |
| Hv_Contig_981_nlr_1 | chr3B | chr3H | chr3D | chr3V |
| Hv_Contig_984_nlr_1 | chr2A | chr2H | chr2D | chr2V |
| Hv_Contig_992_nlr_1 | chr1A | chr1H | chr1D | chr1V |
| Hv_Contig_993_nlr_1 | chr1D | chr1H | chr1D | chr1V |
| Hv_Contig_994_nlr_1 | chr7B | chr7H | chr1D | chr7V |
| Hv_Contig_996_nlr_1 | chr1B | chr1H | chr1D | chr1V |
| Hv_Contig_997_nlr_1 | chr3D | chr3H | chr3D | chr3V |
| Hv_Contig_998_nlr_1 | chr3D | chr3H | chr3D | chr3V |
| Hv_Contig_1000_nlr_1 | chr7A | chr7H | chr7D | chr7V |
| Hv_Contig_1002_nlr_1 | chr6B | chr6H | chr6D | chr6V |
| Hv_Contig_1005_nlr_1 | chr1B | chr1H | chr1D | chr1V |
| Hv_Contig_1007_nlr_1 | chr1D | chr1H | chr1D | chr1V |
| Hv_Contig_1008_nlr_1 | chr3B | chr3H | chr3D | chr3V |
| Hv_Contig_1010_nlr_1 | chr7B | No hit | chr7D | chr7V |
| Hv_Contig_1016_nlr_1 | chr7A | chr7H | chr7D | chr7V |
| Hv_Contig_1020_nlr_1 | chr3D | chr3H | chr3D | chr3V |
| Hv_Contig_1021_nlr_1 | chr7A | chr7H | chr7D | chr7V |
| Hv_Contig_1022_nlr_1 | chr5D | chr5H | chr5D | chr5V |
| Hv_Contig_1024_nlr_1 | chr7A | chr7H | chr1D | chr7V |
| Hv_Contig_1026_nlr_1 | chr1A | chr1H | chr1D | chr1V |
| Hv_Contig_1027_nlr_1 | chr3D | chr3H | chr3D | chr3V |
| Hv_Contig_1028_nlr_1 | chr2D | chr2H | chr2D | chr2V |
| Hv_Contig_1029_nlr_1 | chr7B | chr7H | chr7D | chr7V |
| Hv_Contig_1031_nlr_1 | chr3D | chr3H | chr3D | chr3V |
| Hv_Contig_1032_nlr_1 | chr5D | chr5H | chr5D | chr5V |
| Hv_Contig_1037_nlr_1 | chr3B | chr3H | chr3D | chr3V |
| Hv_Contig_1038_nlr_1 | chr7B | chr7H | chr7D | chr7V |
| Hv_Contig_1041_nlr_1 | chr3D | chr3H | chr3D | chr3V |
| Hv_Contig_1042_nlr_1 | chr2D | chr2H | chr2D | chr2V |
| Hv_Contig_1044_nlr_1 | chr6A | chr5H | chr6D | chr6V |
| Hv_Contig_1045_nlr_1 | chr5A | chr6H | chr5D | chr5V |
| Hv_Contig_1046_nlr_1 | chr7B | chr7H | chr7D | chr7V |
| Hv_Contig_1048_nlr_1 | chr7D | chr7H | chr7D | chr7V |
| Hv_Contig_1049_nlr_1 | chr7D | chr7H | chr7D | chr7V |
| Hv_Contig_1054_nlr_1 | chr5B | chr5H | chr5D | chr5V |
| Hv_Contig_1056_nlr_1 | chr7B | chr7H | chr7D | chr7V |
| Hv_Contig_1060_nlr_1 | chr1D | chr6H | chr1D | chr1V |
| Hv_Contig_1063_nlr_1 | chr2B | chr2H | chr2D | chr2V |
| Hv_Contig_1065_nlr_1 | chr7D | chr7H | chr7D | chr7V |
| Hv_Contig_1067_nlr_1 | chr1B | chr1H | chr1D | chr1V |
| Hv_Contig_1069_nlr_1 | chr5D | chr7H | chr5D | chr5V |
| Hv_Contig_1079_nlr_1 | chr1B | chr1H | chr1D | chr1V |
| Hv_Contig_1083_nlr_1 | chr2A | chr2H | chr2D | chr2V |
| Hv_Contig_1087_nlr_1 | chr7A | chr7H | chr7D | chr7V |
| Hv_Contig_1089_nlr_1 | chr7A | chr1H | chr7D | chr7V |
| Hv_Contig_1092_nlr_1 | chr3A | chr3H | chr3D | chr3V |
| Hv_Contig_1095_nlr_1 | chr7D | chr7H | chr7D | chr7V |
| Hv_Contig_1096_nlr_1 | chr1A | chr3H | chr1D | chr1V |
| Hv_Contig_1097_nlr_1 | chr7D | chr7H | chr7D | chr7V |
| Hv_Contig_1098_nlr_1 | chr3B | chr3H | chr3D | chr3V |
| Hv_Contig_1100_nlr_1 | chr7B | chr7H | chr7D | chr7V |
| Hv_Contig_1103_nlr_1 | chr5D | chr5H | chr5D | chr2V |
| Hv_Contig_1111_nlr_1 | chr3B | chr3H | chr3D | chr3V |
| Hv_Contig_1113_nlr_1 | chr7A | chr7H | chr7D | chr7V |
| Hv_Contig_1115_nlr_1 | chr6A | chr6H | chr6D | chr6V |
| Hv_Contig_1135_nlr_1 | chr3B | chr3H | chr3D | chr3V |
| Hv_Contig_1143_nlr_1 | chr6A | chr6H | chr6D | chr6V |
| Hv_Contig_1145_nlr_1 | chr6D | chr6H | chr6D | chr6V |
| Hv_Contig_1146_nlr_1 | chr7D | chr5H | chr7D | chr7V |
| Hv_Contig_1147_nlr_1 | chr3B | chr3H | chr3D | chr3V |
| Hv_Contig_1150_nlr_1 | chr1D | chr1H | chr1D | chr1V |
| Hv_Contig_1151_nlr_2 | chr6B | chr6H | chr6D | chr6V |
| Hv_Contig_1154_nlr_2 | chr7D | chr7H | chr7D | chr7V |
| Hv_Contig_1155_nlr_1 | chr2B | chr2H | chr2D | chr2V |
| Hv_Contig_1157_nlr_1 | chr7D | chr5H | chr7D | chr7V |
| Hv_Contig_1157_nlr_2 | chr7D | chr5H | chr7D | chr7V |
| Hv_Contig_1158_nlr_1 | chr4B | chr4H | chr4D | chr4V |
| Hv_Contig_1159_nlr_3 | chr3B | chr3H | chr3D | chr3V |
| Hv_Contig_1161_nlr_1 | chr6A | chr6H | chr6D | chr6V |
| Hv_Contig_1163_nlr_1 | chr7B | chr7H | chr7D | chr7V |
| Hv_Contig_1164_nlr_1 | chr1D | chr1H | chr1D | chr1V |
| Hv_Contig_1166_nlr_2 | chr3B | chr3H | chr3D | chr3V |
| Hv_Contig_1168_nlr_1 | chr2D | chr2H | chr2D | chr2V |
| Hv_Contig_1169_nlr_1 | chr1B | chr1H | chr1D | chr1V |
| Hv_Contig_1170_nlr_2 | chr7D | chr7H | chr7D | chr7V |
| Hv_Contig_1173_nlr_1 | chr1B | chr1H | chr1D | chr1V |
| Hv_Contig_1175_nlr_1 | chr2A | chr1H | chr1D | chr1V |
| Hv_Contig_1178_nlr_3 | chr3B | chr3H | chr3D | chr3V |
| Hv_Contig_1183_nlr_1 | chr6A | chr6H | chr6D | chr6V |
| Hv_Contig_1186_nlr_1 | chr1B | chr1H | chr1D | chr1V |
| Hv_Contig_1187_nlr_1 | chr6A | chr6H | chr6D | chr6V |
| Hv_Contig_1191_nlr_1 | chr6D | chr6H | chr6D | chr6V |
| Hv_Contig_1192_nlr_1 | chr3D | chr3H | chr3D | chr3V |
| Hv_Contig_1201_nlr_1 | chr1B | chr1H | chr3D | chr1V |
| Hv_Contig_1202_nlr_1 | chr1A | chr1H | chr1D | chr1V |
| Hv_Contig_1204_nlr_1 | chr3B | chr3H | chr3D | chr3V |
| Hv_Contig_1205_nlr_1 | chr3D | chr3H | chr3D | chr3V |
| Hv_Contig_1211_nlr_1 | chr1B | chr1H | chr3D | chr1V |
| Hv_Contig_1213_nlr_1 | chr1B | chr1H | chr3D | chr1V |
| Hv_Contig_1215_nlr_1 | chr1A | chr1H | chr1D | chr1V |
| Hv_Contig_1217_nlr_1 | chr6B | chr6H | chr6D | chr6V |
| Hv_Contig_1218_nlr_1 | chr6D | chr6H | chr6D | chr6V |
| Hv_Contig_1219_nlr_1 | chr6A | chr6H | chr6D | chr6V |
| Hv_Contig_1220_nlr_1 | chr7A | chr7H | chr7D | chr7V |
| Hv_Contig_1221_nlr_1 | chr7A | chr7H | chr7D | chr7V |
| Hv_Contig_1223_nlr_1 | chr1D | chr1H | chr1D | chr1V |
| Hv_Contig_1228_nlr_1 | chr5B | chr5H | chr5D | chr5V |
| Hv_Contig_1230_nlr_1 | chr6B | chr6H | chr6D | chr6V |
| Hv_Contig_1232_nlr_1 | chr2A | chr2H | chr2D | chr2V |
| Hv_Contig_1233_nlr_1 | chr5D | chr5H | chr5D | chr5V |
| Hv_Contig_1236_nlr_1 | chr7D | chr7H | chr7D | chr7V |
| Hv_Contig_1239_nlr_1 | chr7A | chr7H | chr7D | chr7V |
| Hv_Contig_1240_nlr_2 | chr1A | chr1H | chr1D | chr1V |
| Hv_Contig_1241_nlr_1 | chr3A | chr3H | chr3D | chr3V |
| Hv_Contig_1249_nlr_1 | chr3B | chr3H | chr3D | chr3V |
| Hv_Contig_1250_nlr_2 | chr7D | chr7H | chr7D | chr7V |
| Hv_Contig_1251_nlr_1 | chr7D | chr7H | chr7D | chr7V |
| Hv_Contig_1252_nlr_2 | chr7B | chr7H | chr7D | chr7V |
| Hv_Contig_1253_nlr_1 | chr6A | chr6H | chr6D | chr6V |
| Hv_Contig_1255_nlr_1 | chr2D | chr2H | chr2D | chr2V |
| Hv_Contig_1256_nlr_1 | chr4B | chr4H | chr4D | chr4V |
| Hv_Contig_1257_nlr_1 | chr3B | chr3H | chr3D | chr3V |
| Hv_Contig_1267_nlr_1 | chr6D | chr6H | chr6D | chr6V |
| Hv_Contig_1268_nlr_1 | chr3A | chr3H | chr3D | chr3V |
| Hv_Contig_1272_nlr_1 | chr7D | chr7H | chr7D | chr7V |
| Hv_Contig_1277_nlr_1 | chr7D | chr7H | chr7D | chr7V |
| Hv_Contig_1278_nlr_1 | chr6D | chr6H | chr6D | chr6V |
| Hv_Contig_1285_nlr_1 | chr7B | chr7H | chr7D | chr7V |
| Hv_Contig_1287_nlr_1 | chr1D | chr1H | chr1D | chr1V |
| Hv_Contig_1291_nlr_1 | chr6D | chr6H | chr6D | chr6V |
| Hv_Contig_1292_nlr_1 | chr7D | chr7H | chr7D | chr7V |
| Hv_Contig_1297_nlr_1 | chr1B | chr1H | chr1D | chr1V |
| Hv_Contig_1299_nlr_1 | chr7B | chr7H | chr7D | chr7V |
| Hv_Contig_1300_nlr_1 | chr7D | chr7H | chr7D | chr7V |
| Hv_Contig_1304_nlr_1 | chr7A | chr7H | chr7D | chr7V |
| Hv_Contig_1306_nlr_1 | chr3B | chr3H | chr3D | chr3V |
| Hv_Contig_1307_nlr_1 | chr1B | chr1H | chr1D | chr1V |
| Hv_Contig_1310_nlr_1 | chr3B | chr3H | chr3D | chr3V |
| Hv_Contig_1311_nlr_1 | chr3B | chr3H | chr3D | chr3V |
| Hv_Contig_1314_nlr_1 | chr2B | chr2H | chr2D | chr2V |
| Hv_Contig_1315_nlr_1 | chr1B | chr1H | chr1D | chr1V |
| Hv_Contig_1317_nlr_1 | chr5D | chr5H | chr5D | chr5V |
| Hv_Contig_1318_nlr_1 | chr7D | chr7H | chr7D | chr7V |
| Hv_Contig_1319_nlr_1 | chr1B | chr1H | chr1D | chr1V |
| Hv_Contig_1323_nlr_1 | chr3D | chr3H | chr3D | chr3V |
| Hv_Contig_1328_nlr_1 | chr7D | chr7H | chr7D | chr7V |
| Hv_Contig_1332_nlr_1 | chr3A | chr3H | chr3D | chr3V |
| Hv_Contig_1333_nlr_1 | chr5B | chr5H | chr5D | chr5V |
| Hv_Contig_1334_nlr_1 | chr1A | chr1H | chr1D | chr1V |
| Hv_Contig_1336_nlr_1 | chr7D | chr7H | chr7D | chr7V |
| Hv_Contig_1337_nlr_1 | chr7A | chr7H | chr7D | chr7V |
| Hv_Contig_1338_nlr_1 | chr7D | chr7H | chr7D | chr7V |
| Hv_Contig_1341_nlr_2 | chr3A | chr3H | chr5D | chr3V |
| Hv_Contig_1343_nlr_1 | chr5D | chr5H | chr5D | chr5V |
| Hv_Contig_1345_nlr_1 | chr5D | chr5H | chr5D | chr5V |
| Hv_Contig_1346_nlr_1 | chr1D | chr1H | chr1D | chr1V |
| Hv_Contig_1350_nlr_1 | chr7D | chr7H | chr7D | chr7V |
| Hv_Contig_1352_nlr_1 | chr7A | chr7H | chr7D | chr7V |
| Hv_Contig_1356_nlr_1 | chr1D | chr1H | chr1D | chr1V |
| Hv_Contig_1361_nlr_1 | chr4A | chr7H | chr7D | chr7V |
| Hv_Contig_1362_nlr_1 | chr7B | chr7H | chr7D | chr7V |
| Hv_Contig_1363_nlr_1 | chr5D | chr5H | chr5D | chr5V |
| Hv_Contig_1365_nlr_1 | chr3A | chr3H | chr3D | chr3V |
| Hv_Contig_1370_nlr_1 | chr1B | chr1H | chr1D | chr1V |
| Hv_Contig_1374_nlr_1 | chr6A | chrUn | chr3D | chr6V |
| Hv_Contig_1375_nlr_1 | chr3B | chr3H | chr3D | chr3V |
| Hv_Contig_1376_nlr_1 | chr7D | chr7H | chr7D | chr7V |
| Hv_Contig_1379_nlr_1 | chr1A | chr1H | chr1D | chr1V |
| Hv_Contig_1385_nlr_1 | chr1B | chr1H | chr1D | chr1V |
| Hv_Contig_1386_nlr_1 | chr1A | chr1H | chr1D | chr1V |
| Hv_Contig_1388_nlr_1 | chr2D | chr2H | chr2D | chr2V |
| Hv_Contig_1391_nlr_1 | chr1A | chr1H | chr1D | chr1V |
| Hv_Contig_1392_nlr_1 | chr1B | chr1H | chr1D | chr1V |
| Hv_Contig_1394_nlr_1 | chr4B | chr2H | chr2D | chr2V |
| Hv_Contig_1395_nlr_1 | chr5D | chr7H | chr5D | chr5V |
| Hv_Contig_1397_nlr_1 | chr6B | chr6H | chr6D | chr6V |
| Hv_Contig_1398_nlr_1 | chr3A | chr3H | chr3D | chr3V |
| Hv_Contig_1400_nlr_1 | chr3B | chr3H | chr3D | chr3V |
| Hv_Contig_1402_nlr_1 | chr7A | chr7H | chr7D | chr7V |
| Hv_Contig_1406_nlr_1 | chr6D | chr6H | chr6D | chr6V |
| Hv_Contig_1408_nlr_1 | chr7B | chr7H | chr1D | chr7V |
| Hv_Contig_1410_nlr_1 | chr7D | chr7H | chr7D | chr7V |
| Hv_Contig_1411_nlr_1 | chr7A | chr7H | chr7D | chr7V |
| Hv_Contig_1412_nlr_1 | chr4A | chr7H | chr7D | chr7V |
| Hv_Contig_1415_nlr_1 | chr7B | chr7H | chr7D | chr7V |
| Hv_Contig_1416_nlr_1 | chr5D | chr5H | chr5D | chr5V |
| Hv_Contig_1419_nlr_1 | chr7D | chr7H | chr7D | chr7V |
| Hv_Contig_1423_nlr_1 | chr2A | chr2H | chr2D | chr2V |
| Hv_Contig_1424_nlr_1 | chr6D | chr6H | chr6D | chr6V |
| Hv_Contig_1425_nlr_1 | chr5B | chr5H | chr5D | chr5V |
| Hv_Contig_1427_nlr_1 | chr3B | chr3H | chr3D | chr3V |
| Hv_Contig_1429_nlr_1 | chr2D | chr2H | chr2D | chr2V |
| Hv_Contig_1430_nlr_1 | chr2A | chr2H | chr2D | chr2V |
| Hv_Contig_1431_nlr_1 | chr4A | chr7H | chr5D | chr7V |
| Hv_Contig_1433_nlr_1 | chr1D | chr1H | chr1D | chr1V |
| Hv_Contig_1434_nlr_1 | chr1B | chr1H | chr1D | chr1V |
| Hv_Contig_1439_nlr_1 | chr5A | chr5H | chr5D | chr5V |
| Hv_Contig_1448_nlr_1 | chr7D | chr7H | chr7D | chr7V |
| Hv_Contig_1452_nlr_1 | chr6B | chr6H | chr6D | chr6V |
| Hv_Contig_1453_nlr_1 | chr1D | chr1H | chr1D | chr1V |
| Hv_Contig_1454_nlr_1 | chr1B | chr1H | chr1D | chr1V |
| Hv_Contig_1459_nlr_1 | chr2D | No hit | chr2D | chr2V |
| Hv_Contig_1461_nlr_1 | chr1A | chr1H | chr1D | chr1V |
| Hv_Contig_1466_nlr_1 | chr7A | chr7H | chr7D | chr7V |
| Hv_Contig_1468_nlr_1 | chr7D | chr7H | chr7D | chr7V |
| Hv_Contig_1469_nlr_1 | chr7B | chr7H | chr7D | chr7V |
| Hv_Contig_1470_nlr_1 | chr4B | chr4H | chr4D | chr4V |
| Hv_Contig_1472_nlr_1 | chr2D | chr2H | chr2D | chr2V |
| Hv_Contig_1473_nlr_1 | chr7A | chr7H | chr7D | chr7V |
| Hv_Contig_1475_nlr_1 | chr3A | chr3H | chr3D | chr3V |
| Hv_Contig_1478_nlr_1 | chr6A | chr6H | chr6D | chr6V |
| Hv_Contig_1483_nlr_1 | chr7D | chr7H | chr7D | chr7V |
| Hv_Contig_1485_nlr_1 | chr3D | chr3H | chr3D | chr3V |
| Hv_Contig_1486_nlr_1 | chr6B | chr6H | chr6D | chr6V |
| Hv_Contig_1488_nlr_1 | chr2D | chr2H | chr2D | chr2V |
| Hv_Contig_1499_nlr_1 | chr5D | chr7H | chr5D | chr5V |
| Hv_Contig_1500_nlr_1 | chr7B | chr7H | chr7D | chr7V |
| Hv_Contig_1508_nlr_1 | chr3D | chr3H | chr3D | chr3V |
