# Supplementary material for: Genome-wide identification of the NLR gene family in Haynaldia villosa by SMRT-RenSeq
Source: BMC Genomics. 2022 Feb 10;23:118. doi: 10.1186/s12864-022-08334-w (PMC8832786; doi:10.1186/s12864-022-08334-w)
Supplement: Supplementary file 7 — Additional file 7. [file 12864_2022_8334_MOESM7_ESM.docx]

**Table S4. The annotated NLRs in the removed 53 contigs**.

| **Contig** | **Classification of NLR annotation** | **Identify of the complete NLRs to the unique NLRs** | **Removed or presented** |
| --- | --- | --- | --- |
| Hv_Contig_1 | No NLR locus |  | removed |
| Hv_Contig_76 | No NLR locus |  | removed |
| Hv_Contig_680 | No NLR locus |  | removed |
| Hv_Contig_956 | No NLR locus |  | removed |
| Hv_Contig_1003 | No NLR locus |  | removed |
| Hv_Contig_1366 | No NLR locus |  | removed |
| Hv_Contig_301 | partial NLR |  | removed |
| Hv_Contig_375 | partial NLR |  | removed |
| Hv_Contig_416 | partial NLR |  | removed |
| Hv_Contig_427 | partial NLR |  | removed |
| Hv_Contig_1109 | partial NLR |  | removed |
| Hv_Contig_1496 | partial NLR |  | removed |
| Hv_Contig_1498 | partial NLR |  | removed |
| Hv_Contig_1506 | partial NLR |  | removed |
| Hv_Contig_91 | complete (pseudogene) |  | removed |
| Hv_Contig_278 | complete (pseudogene) |  | removed |
| Hv_Contig_635 | complete (pseudogene) |  | removed |
| Hv_Contig_915 | complete (pseudogene) |  | removed |
| Hv_Contig_960 | complete (pseudogene) |  | removed |
| Hv_Contig_961 | complete (pseudogene) |  | removed |
| Hv_Contig_1244 | complete (pseudogene) |  | removed |
| Hv_Contig_1270 | complete (pseudogene) |  | removed |
| Hv_Contig_164 | complete | 99.87% | removed |
| Hv_Contig_288 | complete | 99.06% | removed |
| Hv_Contig_344 | complete | 99.53% | removed |
| Hv_Contig_520 | complete | 99.59% | removed |
| Hv_Contig_562 | complete | 99.40% | removed |
| Hv_Contig_573 | complete | 99.14% | removed |
| Hv_Contig_608 | complete | 99.77% | removed |
| Hv_Contig_784 | complete | 99.47% | removed |
| Hv_Contig_839 | complete | 99.10% | removed |
| Hv_Contig_1074 | complete | 99.60% | removed |
| Hv_Contig_1348 | complete | 99.40% | removed |
| Hv_Contig_1369 | complete | 99.16% | removed |
| Hv_Contig_1382 | complete | 99.34% | removed |
| Hv_Contig_1405 | complete | 99.52% | removed |
| Hv_Contig_1449 | complete | 99.19% | removed |
| Hv_Contig_1477 | complete | 99.30% | removed |
| Hv_Contig_117 | complete | 98.05% | presented |
| Hv_Contig_201 | complete | 95.81% | presented |
| Hv_Contig_313 | complete | 95.09% | presented |
| Hv_Contig_346 | complete | 98.64% | presented |
| Hv_Contig_380 | complete | 95.09% | presented |
| Hv_Contig_607 | complete | 96.53% | presented |
| Hv_Contig_726 | complete | 98.72% | presented |
| Hv_Contig_748 | complete | 97.70% | presented |
| Hv_Contig_783 | complete | 98.94% | presented |
| Hv_Contig_838 | complete | 97.92% | presented |
| Hv_Contig_903 | complete | 95.21% | presented |
| Hv_Contig_986 | complete | 95.37% | presented |
| Hv_Contig_1068 | complete | 95.41% | presented |
| Hv_Contig_1105 | complete | 96.07% | presented |
| Hv_Contig_1282 | complete | 98.43% | presented |
