# Supplementary material for: Genome-wide identification of the NLR gene family in Haynaldia villosa by SMRT-RenSeq
Source: BMC Genomics. 2022 Feb 10;23:118. doi: 10.1186/s12864-022-08334-w (PMC8832786; doi:10.1186/s12864-022-08334-w)
Supplement: Supplementary file 8 — Additional file 8. [file 12864_2022_8334_MOESM8_ESM.pdf]

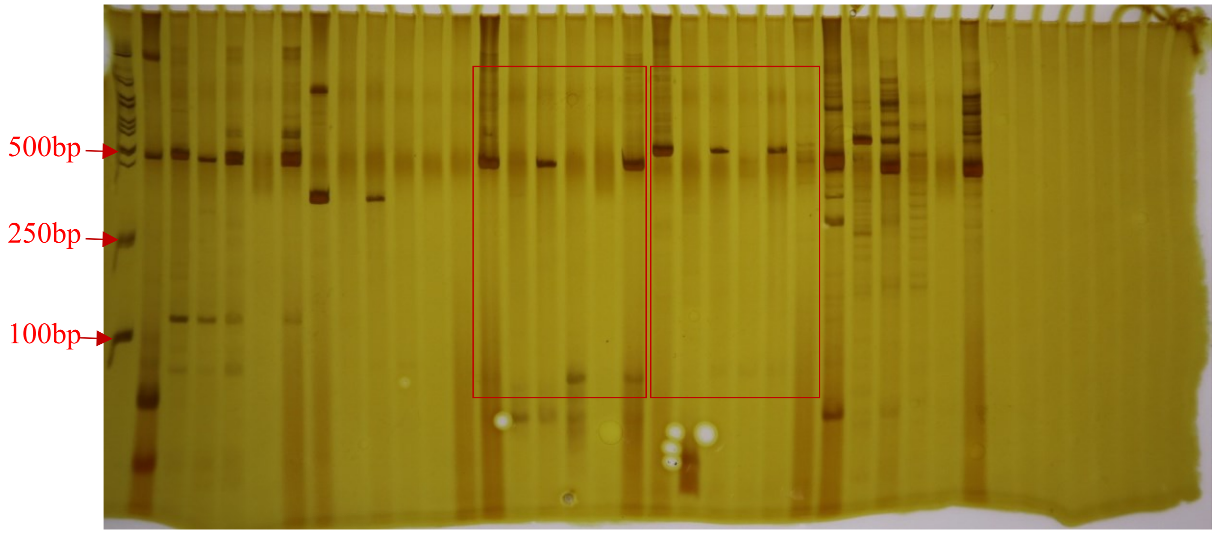

Hv_Contig_254_nlr_1 and Hv_Contig_140_nlr_1

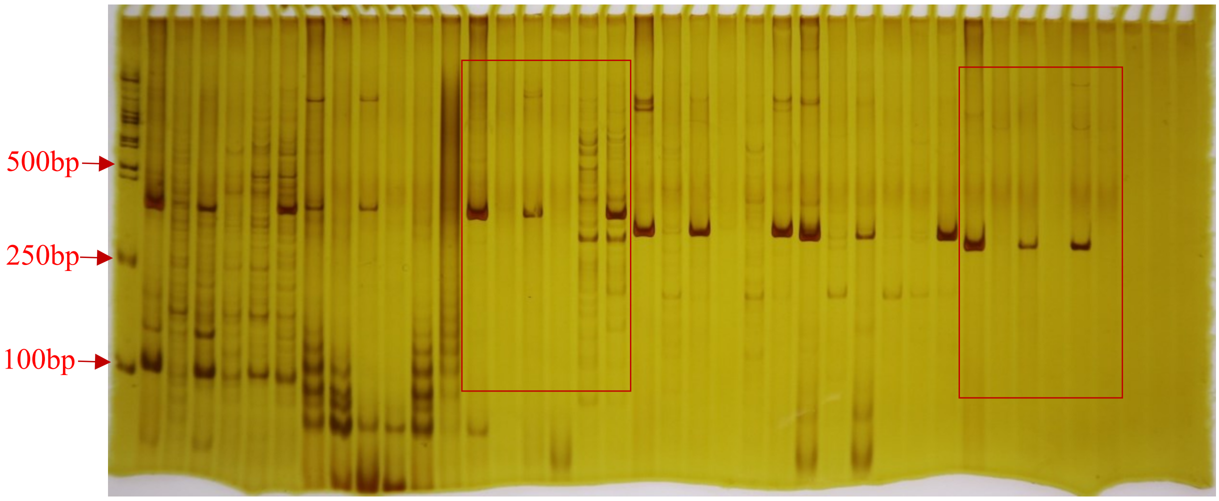

Hv_Contig_223_nlr_1 and Hv_Contig_512_nlr_1

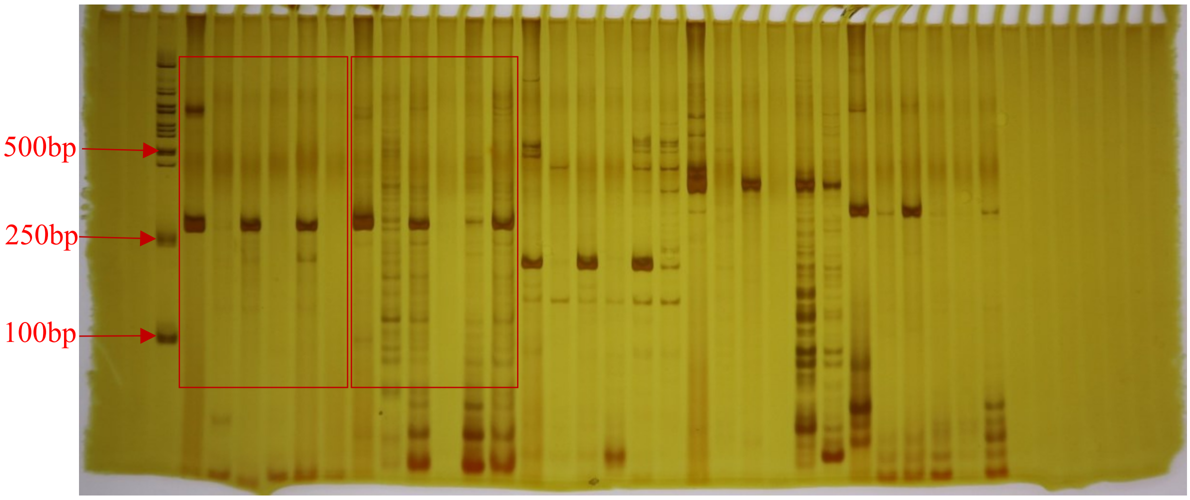

Hv_Contig_782_nlr_1 and Hv_Contig_79_nlr_1

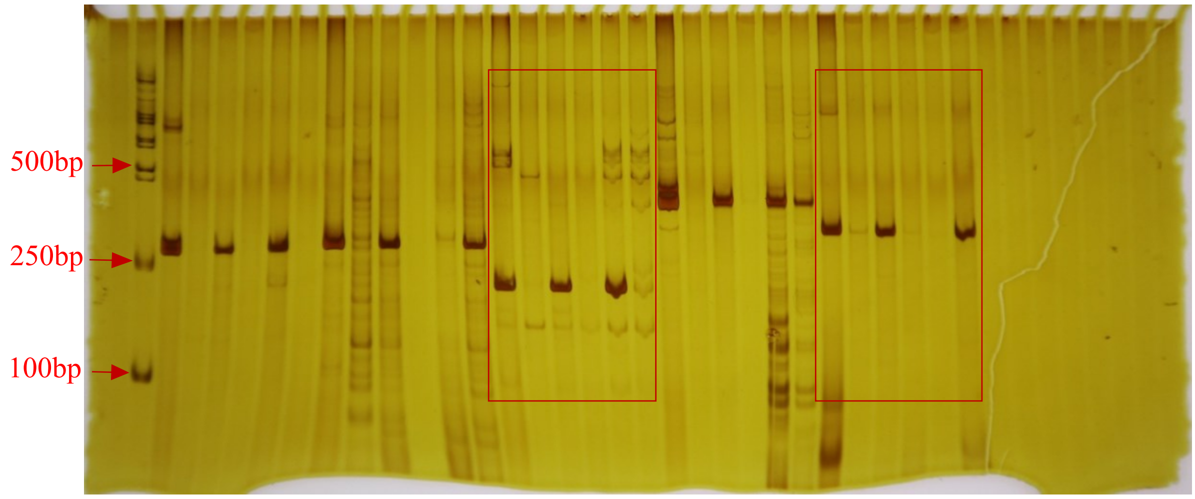

Hv_Contig_322_nlr_2 and Hv_Contig_648_nlr_1

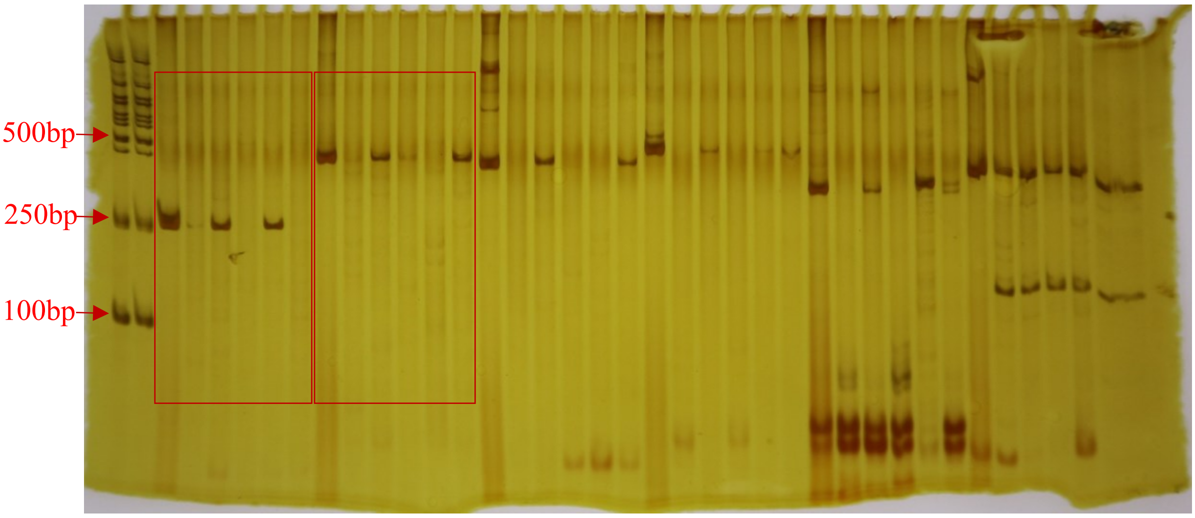

Hv_Contig_541_nlr_1 and Hv_Contig_950_nlr_1

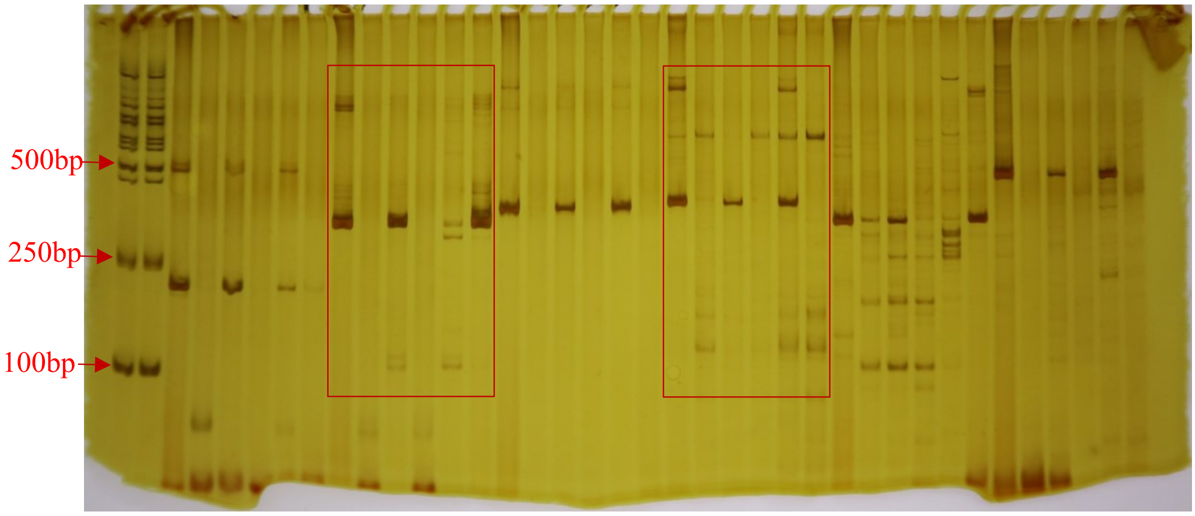

Hv_Contig_249_nlr_2 and Hv_Contig_514_nlr_1

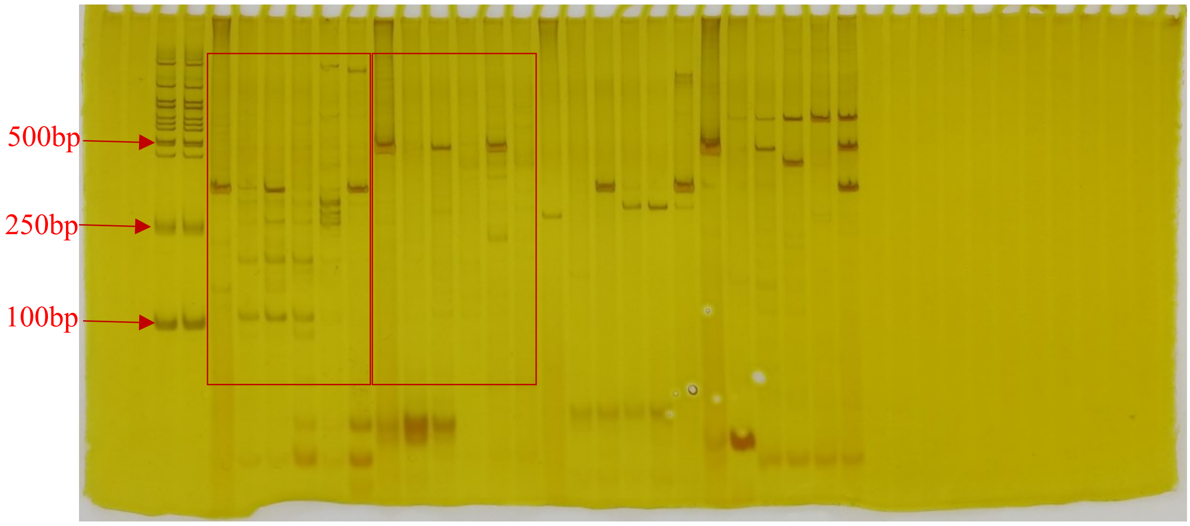

Hv_Contig_414 and Hv_Contig_632

Additional file 8. The full blots gels corresponding to the individual figures in Figure 3.

The six lanes framed in each red rectangle showed the PCR products generated by the specific primer corresponding to the contig indicated under the gel. In each gel, the PCR products corresponding to two contigs were showed, one from the short arm and the other from the long arm. The red arrow marks the position and size of the Marker.
